# Selective Co‐Encapsulation Inside an M_6_L_4_ Cage

**DOI:** 10.1002/chem.201603017

**Published:** 2016-09-14

**Authors:** Stefan H. A. M. Leenders, René Becker, Tatu Kumpulainen, Bas de Bruin, Tomohisa Sawada, Taito Kato, Makoto Fujita, Joost N. H. Reek

**Affiliations:** ^1^ Van't Hoff Institute for Molecular SciencesUniversity of AmsterdamScience Park 9041098 XHAmsterdamThe Netherlands; ^2^ Department of Applied ChemistrySchool of EngineeringThe University of Tokyo7-3-1 Hongo, Bunkyo-kuTokyo113-8656Japan

**Keywords:** charge-transfer complexes, co-encapsulation, host–guest systems, supramolecular chemistry, ternary structures

## Abstract

There is broad interest in molecular encapsulation as such systems can be utilized to stabilize guests, facilitate reactions inside a cavity, or give rise to energy‐transfer processes in a confined space. Detailed understanding of encapsulation events is required to facilitate functional molecular encapsulation. In this contribution, it is demonstrated that Ir and Rh‐Cp‐type metal complexes can be encapsulated inside a self‐assembled M_6_L_4_ metallocage only in the presence of an aromatic compound as a second guest. The individual guests are not encapsulated, suggesting that only the pair of guests can fill the void of the cage. Hence, selective co‐encapsulation is observed. This principle is demonstrated by co‐encapsulation of a variety of combinations of metal complexes and aromatic guests, leading to several ternary complexes. These experiments demonstrate that the efficiency of formation of the ternary complexes depends on the individual components. Moreover, selective exchange of the components is possible, leading to formation of the most favorable complex. Besides the obvious size effect, a charge‐transfer interaction may also contribute to this effect. Charge‐transfer bands are clearly observed by UV/Vis spectrophotometry. A change in the oxidation potential of the encapsulated electron donor also leads to a shift in the charge‐transfer energy bands. As expected, metal complexes with a higher oxidation potential give rise to a higher charge‐transfer energy and a larger hypsochromic shift in the UV/Vis spectrum. These subtle energy differences may potentially be used to control the binding and reactivity of the complexes bound in a confined space.

## Introduction

Supramolecular chemistry has evolved to a stage whereby a large variety of well‐defined structures have been prepared by assembly of small buildings blocks. Now that control over the shape and structure of these nano‐sized objects has been well established, the introduction and evaluation of functional properties is a more pressing concern. Self‐assembled cages typically function as hosts for small molecules that can bind in their interior. The binding of such guest molecules is generally based on a combination of noncovalent interactions, including hydrogen bonds, ionic or π‐π interactions, and hydrophobic effects. Shape complementarity is obviously important, and it has been established that the ideal size of the guest is about 55 % of the size of the interior of the cage.^1^ Typically, these self‐assembled hosts are interesting objects for exploring chemistry with the compounds in their confined spaces.^2^, ^3^, ^4^ Indeed, examples of capsule‐induced selectivity in organic reactions have been reported.^5^, ^6^, ^7^ Furthermore, when metal catalysts are captured inside a capsule, the cages resemble to some extent the second coordination sphere typical of enzymes, and as such new reactivity and selectivity may be displayed by the encapsulated catalysts.^8^, ^9^, ^10^, ^11^ Also in the photosynthetic apparatus in nature, the second coordination sphere is of importance, and in analogy chromophoric guests have been encapsulated and subjected to detailed studies. As a result, induced charge‐transfer (CT) complexes or exciplexes have been enforced by the close proximity of guests and hosts. While charge‐transfer complexes can be considered as involving only a weak interaction between the donor and acceptor, they can often be easily observed and characterized due their intense colors. It has been demonstrated that selective encapsulation of guests in preformed hosts, such as cyclodextrins,^12^ cucurbit[8]urils,^13^, ^14^ pillar[5]arenes,^15^ porous coordination polymers,^16^, ^17^ and metallocages,^18^ can be used to obtain new charge‐transfer complexes. Additionally, the formation of charge‐transfer complexes can be used as a driving force to form new supramolecular assemblies.^19^, ^20^ Whereas the encapsulation of a single guest, such as an organic or metal complex, has been reported frequently, the selective co‐ encapsulation of two different guests is not straightforward. Nevertheless, this type of assembly is of particular interest as it facilitates the occurrence of chemistry between two components, a prerequisite for metal‐catalyzed transformations. Herein, we report the selective encapsulation of two different guests inside an octahedral metallocage to form ternary complexes. The ternary complex consists of a Cp‐ligated metal complex that is in close proximity to a flat aromatic guest, which form a charge‐transfer complex as indicated by UV/Vis spectrophotometry. Different aromatic guests and metal complexes can be used to selectively form the ternary complexes, and the binding efficiency depends on the properties of the components. The resulting charge‐transfer complex varies according to the oxidation potential of the electron‐donor metal complex. Understanding selective co‐encapsulation of a metal complex and substrate in a confined space is important for future studies on catalysis in such environments.

## Results and Discussion

### Selective co‐encapsulation and characterization of the ternary complexes

**Figure 1:**
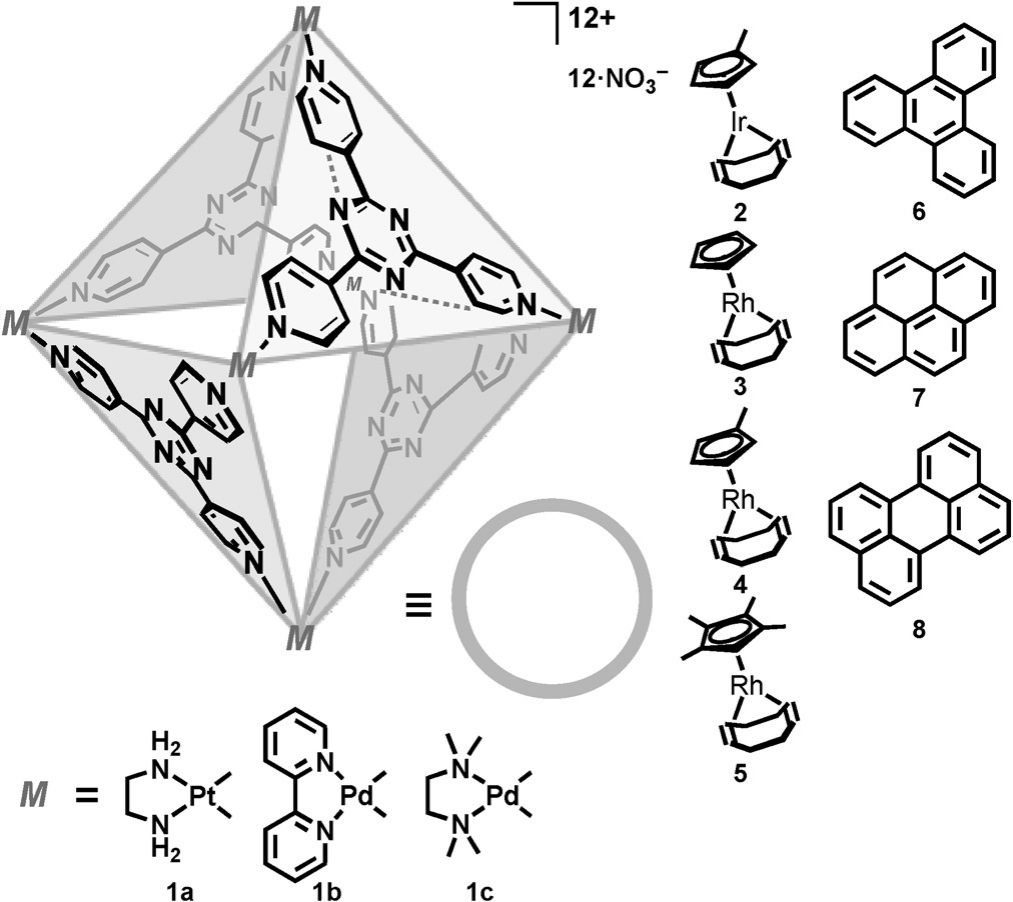
Octahedral‐shaped metallocage **1** and guests used for co‐encapsulation.

A self‐assembled metallocage, which has been reported previously (Figure 1), was used as the host for the current studies. This metallocage has recently been demonstrated to facilitate exciplex formation with different guests^21^, ^22^, ^23^ and to incorporate metal complexes, whereupon a charge‐transfer band was observed.^24^ The water‐soluble octahedral nanosphere **1** (Figure 1) can bind a large variety of guests, mostly on the basis of hydrophobic effects and interactions with the electron‐deficient sidewalls of its cavity. A broad array of guests have been accommodated in this molecular container, including metal complexes,^24^, ^25^, ^26^ substrates for organic reactions,^27^, ^28^ halogens,^29^, ^30^ and a variety of molecules with interesting UV/Vis spectrophotometric properties.^21^, ^22^, ^23^, ^31^ Generally, the host–guest complexes are formed by simply mixing the components.

**Scheme 1:**
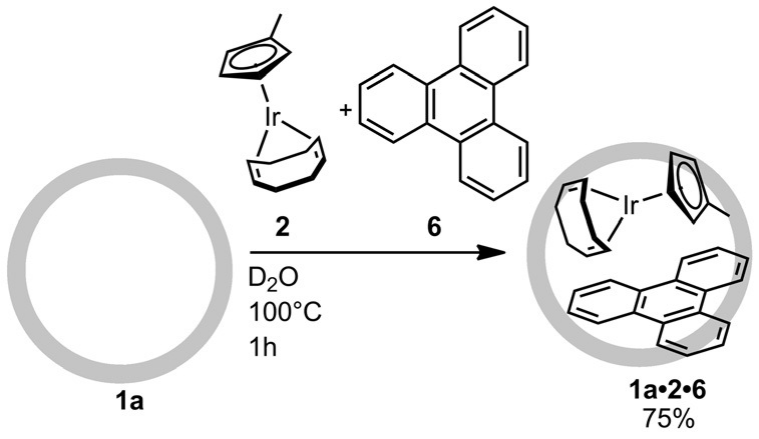
Co‐encapsulation of guests **2** and **6** in host **1 a**.

We investigated whether [(CpMe)Ir(cod)] **2** (Cp=cyclopentadiene, cod=1,5‐cyclooctadiene) could be encapsulated. Stirring a suspension of **2** and **1 a** in D_2_O at 100 °C for prolonged reaction times did not result in significant encapsulation of **2** as no new signals were observed in the NMR spectra. Cage **1** has been reported to bind some guests in the presence of an appropriate second molecule that is co‐encapsulated.^32^, ^33^, ^34^ In this context, we investigated binding of the metal complex in the presence of triphenylene (**6**) as a co‐guest. Heating a suspension of **1 a**, **2**, and **6** in D_2_O at 100 °C for 1 h resulted in a colored suspension. Interestingly, after cooling the solution and filtration of the excess guests, a purple solution was obtained indicating that the ternary complex **1 a⋅2⋅6** had been formed (Scheme 1).

**Figure 2:**
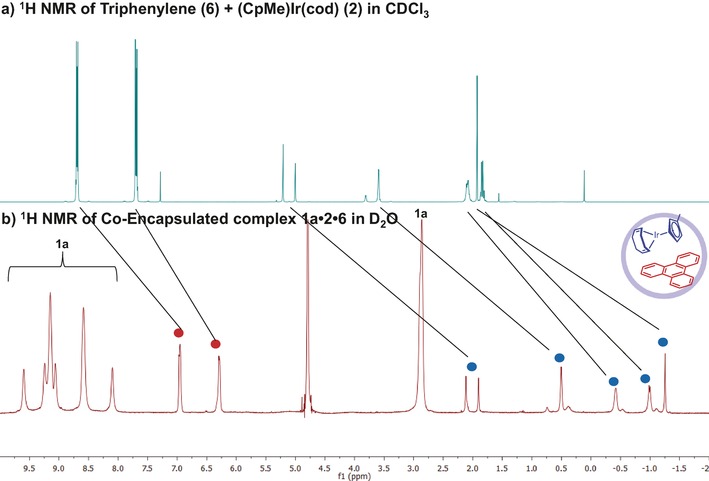
Clear upfield shifts observed in the ^1^H NMR spectra of guests **2** and **6**: spectrum of a mixture of guests in CDCl_3_ (a, top) and inside molecular container **1 a** in D_2_O (b, bottom).

Due to their low solubility in water, neither the iridium complex nor the triphenylene could be detected by ^1^H NMR spectroscopy in D_2_O solution. In contrast, in the presence of the cage, their signals were clearly visible. Binding was confirmed by the clear upfield shifts displayed by the guest molecules in the ^1^H NMR spectrum, as depicted in Figure 2. The shielding caused by the aromatic rings of the cage resulted in typical shifts of the triphenylene signals of 1.7 and 1.4 ppm, whereas the signals of the metal complex were shifted by between 2.5 and 3.2 ppm. Another indication of guest encapsulation was provided by the change of symmetry^34^ of the metallocage from *T*_d_ to effectively *C*_3_ (on the NMR timescale), as was clear from the ^1^H NMR spectra. This loss of symmetry occurs because each guest occupies one half of the capsule. The guests are closely packed against the triazine panel and on the NMR timescale the cage loses its *T*_d_ symmetry^35^ (see Supporting Information S4). For the empty cage, all pyridine rings are equivalent so that only two signals are observed for the pyridine protons. However, for the cage incorporating the two guests, eight sets of pyridine proton signals are observed. Importantly, the symmetry of the guest molecules was not affected by the encapsulation, indicating that these can freely rotate inside the void of the capsule. Additional support for guest encapsulation was provided by diffusion‐ordered NMR (DOSY, depicted in Supporting Information S8), which showed that the diffusion constant of the guests matched that of the capsule.^24^ Due to the enforced close proximity of the respective guests, NOE signals could be observed between them in the 2D NOESY spectrum (see Supporting Information S8). In addition, the guests also displayed NOE contacts with the capsule **1 a**, reflecting a tight fit of the compounds in the metallocage.

**Figure 3:**
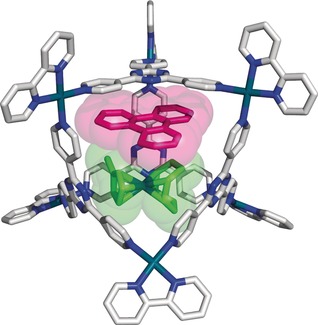
Solid‐state structure of ternary complex **1 b⋅2⋅6** (33.3 % occupancy). The metal complex **2** is shown in green and triphenylene **6** is shown in purple. Solvent molecules and nitrate anions have been omitted for clarity.

Attempts to crystallize the ternary product using metallocage **1 a** did not yield crystalline material suitable for X‐ray diffraction analysis. However, when we changed the nanosphere to its palladium analogue with a 2,2′‐bipyridine *cis*‐capping ligand (metallocage **1 b**),^36^ crystals suitable for X‐ray diffraction analysis were obtained. The data revealed a molecular structure in which the two guests occupy the void of the sphere (Figure 3), showing a tight fit of both guests inside the cage. The guest pair (**2⋅6**) was disordered over three positions, hence only the 33.3 % occupancy is displayed to clearly show the host–guest structure. Although the solid‐state structure cannot be directly compared with the structure in solution, the disorder over three positions corresponds to the effective *C*_3_ symmetry of the host–guest assembly on the NMR timescale, as derived from the NMR spectra in solution at room temperature.

The molecular structure shows that the triphenylene guest (**6**) resides close to the triazine panel. However, it is not stacked completely parallel to the panel. The distance from **6** (central ring) to the central ring of the triazine panel is 3.48 Å, and that from **6** to the iridium atom is 4.84 Å. The average angle between the pyridine moieties of the triazine panels that are connected to the palladium center is 86.4°. This is similar to the average of 86.6° previously reported for the crystal structure of the cage incorporating an adamantoid water molecule cluster.36b

### Scope of the co‐encapsulation

Following the observation that only the two guests together could be embedded inside the cavity of the metallocage and that no individual encapsulation occurs, the scope of the metal complexes and aromatic moieties that can be co‐encapsulated was explored. The ^1^H NMR spectrum of **1 c⋅2⋅6** showed incomplete encapsulation as empty cage **1 c** was still present. Integration of the pyridine proton signals and comparison of these with the aromatic signals allowed determination of the efficiency of the formation of the ternary complex, expressed in terms of percentage of occupied cage; this was 75 % for **1 c⋅2⋅6**. Next, the metal complex was changed to the sterically similar, but electronically distinct, rhodium complex **4**, which resulted in a similar amount of ternary complex (see Table 1). Steric influences on the amount of co‐encapsulation were studied by employing complexes **3** and **5**. A small increase in the formation of ternary complex **1 c⋅3⋅6** was observed (84 % vs. 78 % for **1 c⋅4⋅6**) employing the complex with no methyl groups on the cyclopentadiene ligand. In contrast, a large decrease in co‐encapsulation was observed when four methyl groups were present, resulting in 28 % of complex **1 c⋅5⋅6** (Table 1).

**Table 1:** Formation of the ternary complexes in metallocage **1 c** with metal donors **2**–**5** and aromatic guests **6**–**8**. All encapsulation studies were performed with the same numbers of equivalents of guests for 1 h at 100 °C. Percentages are based on integration of the pyridine ^1^H NMR signals.

	Co‐encapsulation [%]		
	Triphenylene (**6**)	Pyrene (**7**)	Perylene (**8**)
[(CpMe)Ir(cod)] (**2**)	75	98	32
[(Cp)Rh(cod)] (**3**)	84	97	43
[(CpMe)Rh(cod)] (**4**)	78	98	37
[(CpMe_4_)Rh(cod)] (**5**)	28	81	14

**Scheme 2:**
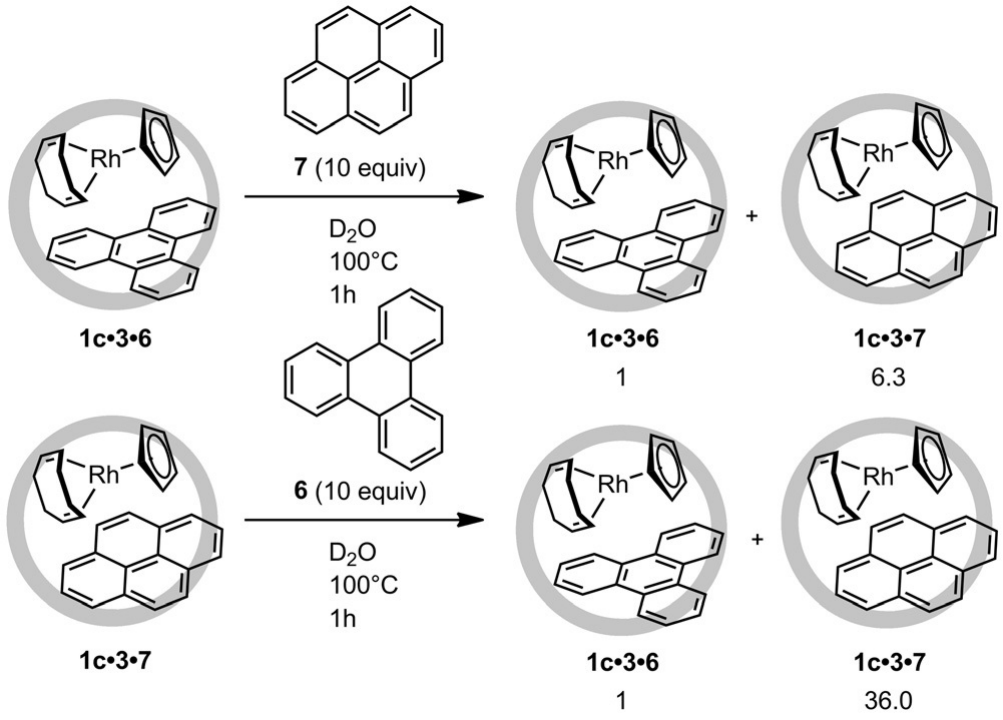
Exchange studies of preformed ternary complexes with pyrene (top) or triphenylene (bottom). These experiments showed the metallocage to have a preference for binding pyrene.

Interested by this difference in the formation of the co‐encapsulated species, we changed the aromatic guest to pyrene (**7**) or perylene (**8**). The ternary complexes formed with pyrene showed a higher amount of co‐encapsulation. With guest **7**, high formation of ternary complex was also observed with the sterically hindered [(CpMe_4_)Rh(cod)] (81 %). Conversely, co‐encapsulation with perylene resulted in lower amounts of formation of the ternary complexes, among which that of **1 c⋅5⋅8** was the lowest, amounting to a mere 14 %. The more efficient formation of ternary complexes with pyrene compared to triphenylene prompted us investigate whether the former guest could displace the latter. A solution of the triphenylene ternary complex **1 c⋅3⋅6** was therefore mixed with 10 equivalents of pyrene at 100 °C for 1 h (depicted in Scheme 2, top). After cooling and filtration of the excess guest, it was clear that exchange of the aromatic guest had taken place, and the ratio of **1 c⋅3⋅6** to **1 c⋅3⋅7** was determined as 1:6.3. Similarly, a solution of **1 c⋅3⋅7** was mixed with triphenylene (**6**) at 100 °C for 1 h. In this case, the ratio of **1 c⋅3⋅6** to **1 c⋅3⋅7** was 1:36.0. Thus, pyrene easily displaces triphenylene and has a higher affinity for the cavity of the metallocage.

To further prove that perylene is thermodynamically the least favored in the void of metallocage **1**, a mixture of excesses of all three aromatic guests (20 equivalents) was stirred with **3** and **1 c**. After 1 h at 100 °C, this resulted in a ratio of **1 c⋅3⋅6**:**1 c⋅3⋅7** of 1:9.1 and no perylene was encapsulated. Heating the suspension with the guests for a longer time (6 h) slightly influenced the ratio (1:10.3) but still did not result in the encapsulation of **8**. This demonstrates that the metallocage has different affinities for the aromatic guests and that this is represented in the amount of the ternary complex that is formed.

### Tuning of the charge‐transfer band through co‐encapsulation

**Figure 4:**
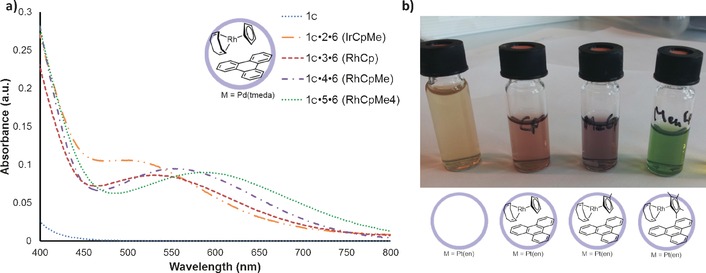
a) UV/Vis absorption spectra indicating the charge‐transfer complexes in capsule **1 c** with triphenylene (250 μm). b) Photograph of the different colored solutions of the ternary complexes, demonstrating that the metal complex changes the color of the solution (in metallocage **1 a**).

Intrigued by the distinct colors that were observed with the different ternary complexes, we proceeded to examine the co‐encapsulated species by UV/Vis spectrophotometry. Upon co‐encapsulation of rhodium complex **4** and triphenylene, the pale‐yellow solution of empty cage **1 c** turned purple and a new absorption band was observed at 555 nm (2.23 eV). This new band is indicative of the formation of a charge‐transfer complex (see Figure 4). To confirm that the cage elicits the new absorption band, we combined the compounds in chloroform. The absence of a charge‐transfer band in the UV/Vis spectrum of this solution demonstrated that pre‐organization in the metallocage is required for exciplex formation.

**Figure 5:**
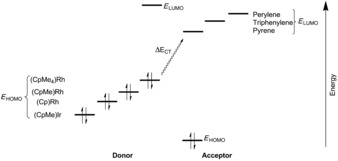
Schematic energy diagram of the charge‐transfer energies facilitated by cage **1 c**. The ordering of the donor and acceptor species is based on measured charge‐transfer energies.

The formation of charge‐transfer complexes of soluble molecules in supramolecular complexes based on cucurbit[8]uril has been reported previously.^13^, ^15^, ^37^ In the current system, formation of the host–guest system is based on extraction of the guests, which are essentially insoluble in water. The metal complexes that are co‐encapsulated by this metallocage make it possible to investigate the effect of the redox potential on the charge‐transfer complex. In this regard, various pairs of guests for the formation of ternary complexes were investigated. The donor in the charge‐transfer complex was expected to be the metal complex, hence various complexes with different steric and electronic properties were studied. The same steric structure was retained and only the electronic properties were changed by utilizing [(CpMe)Ir(cod)] (**2**) for the encapsulation studies. When **2** was co‐encapsulated with triphenylene, the charge‐transfer band was shifted to higher energy (513 nm, 2.42 eV; see Table 2) compared to that obtained with the rhodium complex (**4**). This blue‐shift of the charge‐transfer band was anticipated as the oxidation potential of **5** is higher than that of **3**, as indicated by the redox potentials of the complexes in dichloromethane determined by cyclic voltammetry measurements (see Table 2 and Supporting Information S6). When the electronic properties of the rhodium complex were further changed by substituting it with four methyl groups (complex **5**) as opposed to no methyl groups (complex **3**), it became apparent that metal complexes with a higher oxidation potential yielded a charge‐transfer band with triphenylene at lower wavelength. To the best of our knowledge, this is the first time that metal complexes have been used to fine‐tune charge‐transfer bands through the formation of ternary complexes, based on their redox potentials. To ascertain whether similar trends could be observed, we co‐encapsulated other aromatic guests. For this purpose, pyrene (**7**) and perylene (**8**) were used to form the ternary complexes. With perylene (**8**) as aromatic co‐guest, the charge‐transfer bands shifted to higher energies. A recurring trend in the energies was observed, with the exception of **1 c⋅2⋅8**, for which the energy was difficult to determine due to overlap with the UV band of perylene. The use of pyrene resulted in almost quantitative formation of the ternary complexes (see Table 1), including with the sterically demanding **5**. However, the location of the charge‐transfer band was difficult to determine precisely as it partially overlapped with the absorption of pyrene (see Supporting Information S5). Although the estimated energies were therefore less accurate, all CT bands shifted to lower energies as a consequence of the lower‐lying LUMO of pyrene. This was consistent with the metal complex being the electron donor and the aromatic compound the acceptor. The resulting charge‐transfer complex may be stabilized by the electron‐poor cavity of the molecular container, but the dominant effect is the charge‐transfer complex formation between the two guests as variation of either component leads to changes in the bands. Based on these findings, we constructed a schematic energy diagram for the charge‐transfer interactions, as depicted in Figure 5.

**Table 2:** Charge‐transfer energies (Δ*E*_CT_) of the ternary complexes facilitated by metallocage **1 c**. Energies are based on fitting of the charge‐transfer peaks in UV/Vis spectrophotometry (*λ*_CT_). The charge‐ transfer energies are in line with the redox properties of the metal complex (*E*_ox_), which were determined by cyclic voltammetry.

		Triphenylene (**6**)	Pyrene (**7**)	Perylene (**8**)
Metal complex	*E*_ox_ [V]^[a]^	Δ*E*_CT_ [eV]	Δ*E*_CT_ [eV]	Δ*E*_CT_ [eV]
[(CpMe_4_)Rh(cod)] (**5**)	−0.28	2.13	1.88	2.22
[(CpMe)Rh(cod)] (**4**)	0.02	2.23	2.03^[b]^	2.40
[(Cp)Rh(cod)] (**3**)	0.07	2.34	2.01^[b]^	2.50
[(CpMe)Ir(cod)] (**2**)	0.12	2.42	2.04^[b]^	2.38^[b]^

[a] Oxidation potentials were measured using 1 mm solutions of the metal complex in CH_2_Cl_2_ containing 0.1 m TBAPF_6_ at a glassy carbon working electrode. The potentials are referenced to ferrocene (Fc^0/+^) and based on simultaneous fitting of an irreversible wave at multiple scan rates (0.1/0.3/1.0 V s^−1^); see Experimental Section S6 for more details. [b] Due to overlap of the CT band with the UV band of the aromatic compound, the exact CT energy is difficult to determine and less accurate.

## Conclusion

We have demonstrated selective co‐encapsulation in metallocage **1**, the void of which is occupied by one metal complex and one flat aromatic guest. As the individual guests are not encapsulated under the applied conditions, this result represents an example of selective co‐encapsulation. The shapes of the respective guests complement each other and only together fill the void of the cage. The amount of co‐encapsulation depends on the guests, and varies with their steric properties. Exchange of the guests is possible, and offering multiple suitable guests shows a thermodynamic preference for certain ternary complexes. In the UV/Vis spectra of these complexes, clear charge‐transfer bands are observed, the energy of which is controlled by the electronic properties of the donor (metal complex) and the acceptor (aromatic guest). The void of the metallocage makes it possible to study the formation of various ternary complexes, showing the benefits of these host–guest systems. The possibility of selectively co‐encapsulating two guests is important in the future design of novel host–guest complexes, and could ultimately lead to catalytic transformations inside these metallocages.

## Supporting information

As a service to our authors and readers, this journal provides supporting information supplied by the authors. Such materials are peer reviewed and may be re‐organized for online delivery, but are not copy‐edited or typeset. Technical support issues arising from supporting information (other than missing files) should be addressed to the authors.

Supplementary
