# Supplementary material for: Selective Co‐Encapsulation Inside an M6L4 Cage
Source: Chemistry. 2016 Sep 14;22(43):15468–74. doi: 10.1002/chem.201603017 (PMC5096245; doi:10.1002/chem.201603017)
Supplement: Supplementary file 1 — Supplementary [file CHEM-22-15468-s001.pdf]

CHEMISTRY
A European Journal

Supporting Information

Selective Co-Encapsulation Inside an M_6L_4 Cage

Stefan H. A. M. Leenders,[a] René Becker,[a] Tatu Kumpulainen,[a] Bas de Bruin,[a] Tomohisa Sawada,[b] Taito Kato,[b] Makoto Fujita,[b] and Joost N. H. Reek*[a]

chem_201603017_sm_miscellaneous_information.pdf

Table of Contents

S1. MATERIALS AND METHODS 2

S2. SYNTHESIS OF BUILDING BLOCKS, CAGES AND GUESTS 3

S4. LOSS OF SYMMETRY OF THE METALLOCAGE 8

S5. UV-VIS STUDIES 9

S6. ELECTROCHEMISTRY 16

S7. X-RAY CRYSTAL STRUCTURE OF TERNARY COMPLEX 1B•5•6 19

S8. SPECTRAL DATA OF COMPOUNDS AND CO-ENCAPSULATION PRODUCTS 23

S9. REFERENCES 60

S1. Materials and methods

All reactions were carried out under an nitrogen atmosphere using standard Schlenk techniques when noted. Solvents were distilled prior utilization by conventional methods. NMR spectra were measured on a Bruker AMX 400, DRX 500 and DRX 300 spectrometer. ^{1}H NMR spectral data were referenced to solvent residual signal [7.26 ppm for $CDCl_3$, 4.79 ppm for D_2O], ^{13}C NMR chemical shifts are reported relative to deuterated solvents [77.1 ppm for $CDCl_3$]. 2D ^{1}H-DOSY were performed on DRX 300 spectrometer with temperature and gradient calibration prior to the measurements, and the temperature was controlled at 298 K during the measurements. The log D value of the solvent (D_2O) was consistent each time and in line with the literature.[1] Mass spectra were collected on an AccuToF LC, JMS-T100LP Mass spectrometer (JEOL, Japan) (for ESI) and AccuToF GC v 4g, JMS-T100GCV Mass spectrometer (JEOL, Japan) (for FD). UV-Vis spectra were measured on a Hewlett Packard 8453. Elemental analysis was performed by Microanalytisches Laboratorium Kolbe (Mulheim an der Ruhr, Germany). All reagents were purchased from commercial suppliers and used without further purification.

S2. Synthesis of building blocks, cages and guests

Pt(en)Cl$_2$[2]

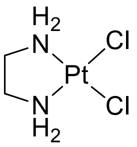

K_2PtCl_4 (2 g, 4.818 mmol, 1 equiv.) was dissolved in water (20 mL) and acidified to a pH of 3 with 1M HCl. Then a solution of ethylenediamine (320 μL, 288 mg, 4.787 mmol, 0.99 equiv.) in water (20 mL) was added. After stirring for 2 h at room temperature a yellow suspension was formed which was filtered. Further washing with water, ethanol and diethyl ether yielded pure product as a yellow solid (1297 mg, 3.978 mmol, 83%). Spectral data in correspondence with literature[2]: ^{1}**H NMR** (300 MHz, [D6]DMSO) δ 5.33 (s, 4H), 2.22 (s, 4H).

Pt(en)(NO$_3$)$_2$

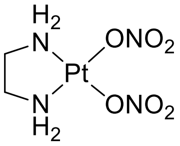

Pt(en)Cl$_2$ (1100 mg, 3.373 mmol, 1 equiv.) was suspended in water (200 mL) and $AgNO_3$ (1146 mg, 6.746 mmol, 2 equiv.) was added. The suspension was stirred with exclusion from light at room temperature for overnight. The resulting white suspension was filtered (or centrifuged: 4000 rpm, 30 min) and concentrated *in vacuo*. This gave 1206 mg of a pale yellow solid (3.180 mmol, 94%). Spectral data in correspondence with literature[3]: ^{1}**H NMR** (400 MHz, D_2O) δ 5.73 (s br, 4H), 2.51 (s, 4H).

Pd(tmeda)Cl$_2$[4]

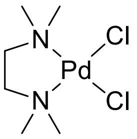

$PdCl_2$ (2g, 11,28 mmol, 1 equiv.) was suspended in acetone (50 mL) and 1681 μL tetramethylethylenediamine, tmeda (11.28 mmol, 1 equiv.) was added. The suspension was stirred at room temperature for 24 h, filtered and washed with water, acetone and diethyl ether to yield 2992 mg of yellow solid (90% yield, 10.19 mmol). Spectral data are in correspondence with literature[4]: ^{1}**H NMR** (300 MHz, [D6]DMSO) δ 2.72 (s, 1H), 2.64 (s, 3H).

Pd(tmeda)(NO$_3$)$_2$

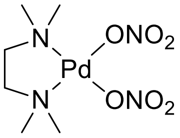

Pd(tmeda)Cl$_2$ (1.5g, 5.11 mmol, 1 equiv.) was suspended in 150 mL of water. With exclusion from light $AgNO_3$ (2 equiv. 10.22 mmol, 1736 mg) was added and stirred at room temperature for overnight. The resulting suspension was then filtered and the filtrate was concentrated *in vacuo* to yield 1.681 g of yellow solid (4.85 mmol, 95%). ^{1}**H NMR** (400 MHz, D_2O) δ 2.89 (s, 1H), 2.68 (s, 3H).

***p*-tpt (*para*-trispyridinetriazine)**

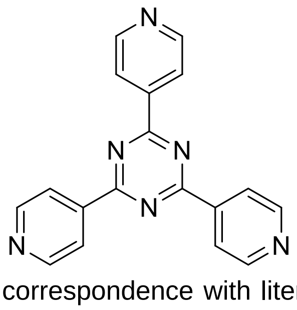

A flask was charged with 4-cyanopyridine (30 g, 288 mmol, 1 equiv.) and heated to 150 °C. Powdered NaOH (1152 mg, 28.8 mmol, 0.1 equiv.) was added to the resulting liquid and the resulting mixture was stirred at 150 °C for 24 h. The solid was washed with acetone (3x180 mL) and dissolved in 250 mL 2M HCl, then norit was added and sonicated for 30 min. The suspension was filtered over Celite and neutralized wit 5M NaOH (approx. 150 mL). The product was filtered and washed with water and acetone. After drying this yielded 17 g of white solid (57% yield).Spectral data are in correspondence with literature[5]: ^{1}**H NMR** (400 MHz, $CDCl_3$) δ 8.98 – 8.91 (m, 1H), 8.61 – 8.54 (m, 1H).

M_6L_4 cage from $Pt(en)(NO_3)_2$[6,7] (1a)

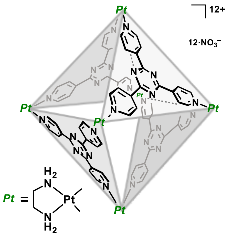

$Pt(en)(NO_3)_2$ (600 mg, 1.582 mmol, 6 equiv.) was dissolved in 30 mL of water in a high pressure tube and *para*-trispyridinetriazine (329 mg, 1.055 mmol, 4 equiv.) was added. The tube was sealed tight and heated to 150 °C for 3 days. The solution was then cooled down, filtered and concentrated *in vacuo* to yield 782 mg of white solid (84%). Spectral data are in correspondence with literature[6]: **^{1}H NMR** (300 MHz, D_2O) δ 9.10 (d, *J* = 5.1 Hz, 24H), 8.58 (d, *J* = 5.4 Hz, 24H), 2.85 (s, 24H).

M_6L_4 cage of $Pd(tmeda)(NO_3)_2$ (1c)

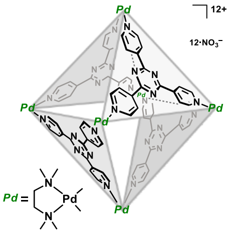

Following a literature procedure,[8] $Pd(tmeda)(NO_3)_2$ (1130 mg, 3.26 mmol, 6 equiv.) was dissolved in 54 mL of H_2O and *p*-tpt (678 mg, 2.17 mmol, 4 equiv.) was added. The resulting suspension was stirred at 80 °C for 1 h. After filtration and concentration *in vacuo* a yellow solid was obtained (1.788 g, 99%). Spectral date are in correspondence with literature[8]: **^{1}H NMR** (400 MHz, D_2O) δ 9.36 – 9.28 (m, 24H), 8.82 – 8.77 (m, 24H), 3.20 (s, 24H), 2.80 (s, 72H).

General procedure for the synthesis of rhodium cyclo-octadiene (cod) complexes
A flame dried schlenk was charged $[(cod)RhCl]_2$ (250 mg, 0.507 mmol, 1 equiv.) and Na_2CO_3 (250 mg, 2.359 mmol, 4.65 equiv.) and flushed with N_2/vacuum for 3 times. Then MeOH was added (25 mL) to the stirring solution followed by consecutive addition of freshly distilled (substituted) cyclopentadiene (9.46 mmol, 18.7 equiv.). The solution was stirred at rt or 60 °C for the indicated time after which it was filtered and concentrated *in vacuo*. The residue was dissolved in Et_2O, filtered and concentrated again. Pure product was obtained by sublimation with a bulb to bulb apparatus at the indicated temperature and under high vacuum (10^{-2} bar) to yield yellow crystals of the desired compound.

CpRh(cod)[9] (3)

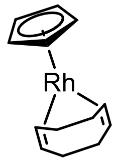

Synthesized according to general procedure: cyclopentadiene was added (625 mg, 795 *μ*L) and stirred for 5 h at 60 °C. Sublimed at 100 °C to give 185 mg of product (0.670 mmol, 66% based on Rh). Spectral data in correspondence with literature.[10] **^{1}H NMR** (400 MHz, C_6D_6) δ 4.95 (s, 5H), 3.97 (s, 4H), 2.34 – 2.12 (m, 4H), 1.95 (m, 4H). **HRMS** (FD+) calcd. for $C_{13}H_{17}Rh$ 276.03853 $[M]^+$, found 276.03768 (*Δ*ppm = 3.1).

(MeCp)Rh(cod) (4)

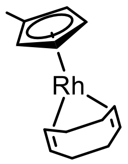

Synthesized according to general procedure: methyl-cyclopentadiene was added (760 mg, 808 *μ*L) and stirred for 4 h at 60 °C and at rt for overnight. Distilled at 140 °C to give 267 mg of product (0.920 mmol, 91% based on Rh) as a yellow liquid which solidifies upon cooling down. **^{1}H NMR** (400 MHz, $CDCl_3$) δ 5.18 (s br, 2H Cp-H), 4.92 (t, *J* = 1.9 Hz, 2H Cp-H), 3.73 (s, 4H COD-CH), 2.29 – 2.11 (m, 4H COD-CH_2), 1.98 – 1.86 (m, 4H COD-CH_2), 1.76 (s, 3H Cp-Me). **^{13}C NMR** (75 MHz, $CDCl_3$) δ 100.61 (d, *J* = 4.0 Hz Cp(C)-Me), 87.81 (d, *J* = 3.7 Hz Cp), 85.17 (d, *J* = 4.1 Hz Cp), 64.11 (d, *J* = 14.3 Hz COD-CH), 32.61 (COD-CH_2), 12.70 (Cp-Me). **HRMS** (FD+) calcd. for $C_{14}H_{19}Rh$ 290.05418 $[M]^+$, found 290.05383 (*Δ*ppm = 1.2). Anal.: found (calcd.) for $C_{14}H_{19}Rh$: C, 57.97 (57.94.73); H, 6.58 (6.60).

$(Me_4Cp)Rh(cod)$ (5)

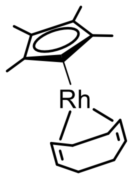

Synthesized according to general procedure: tetramethyl-cyclopentadiene was added (1158 mg, 1433 *μ*L) and stirred for overnight at rt. Sublimed at 130 °C to give 178 mg of product (0.536 mmol, 53% based on Rh). **^{1}H NMR** (500 MHz, $CDCl_3$) δ 5.04 (s, 1H Cp-H), 3.07 (s, 4H COD-CH), 2.27 – 2.12 (m, 4H COD-CH_2), 1.93 (q, *J* = 9.0, 8.1 Hz, 4H COD-CH_2), 1.85 (s, 6H Cp-Me), 1.62 (s, 6H Cp-Me). **^{13}C NMR** (126 MHz, $CDCl_3$) δ 99.51

(d, J = 3.8 Hz Cp-C), 95.72 (d, J = 4.3 Hz Cp-C), 84.96 (d, J = 4.3 Hz Cp-CH), 69.29 (d, J = 14.2 Hz COD-CH), 32.84 (COD-CH_2) , 10.94 (CpMe) , 9.78 (CpMe) . **HRMS** (ESI+) calcd. for $C_{17}H_{25}Rh$ 332.10113 $[M]^+$, found 332.10279 (Δppm = 5.00). Anal.: found (calcd.) for $C_{17}H_{25}Rh$: C, 62.32 (61.45); H, 7.51 (7.58).

S3. Co-encapsulation studies

Standard procedure for the co-encapsulation of CpRh(cod) analogues with aromatic molecules

A vial equipped with stirring bar was charged with cage (5 μmol, 1 equiv.), Rh complex (15 μmol, 3 equiv.) and aromatic molecule (15 μmol, 3 equiv.). The vial was then purged with N_2/vacuum and D_2O was added. The vial was heated to 100 °C for 1 h and filtered over a syringe filter. Yield of encapsulation was based on integration of the aromatic guest signals determining how much the full pyridine integration deviates from the expected 48 pyridine protons. The authors would further like to note that due to the low concentration of the guests, no ^{13}C NMR signals are reported as not all signals could be resolved.

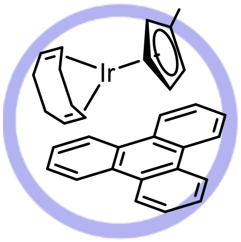

M = Pt(en)

1a•2•6

After encapsulation according to standard procedure, co-encapsulation was observed in 75% according to ^1H NMR. **^{1}H NMR** (400 MHz, D_2O) δ 9.46 (s, br, 7H), 9.11 (s, br, 6H, Py), 9.02 (s br, 6H, Py), 8.92 (s br, 12H, Py+empty **1a**), 8.45 (s, br, 6H, Py), 7.96 (s, br, 6H, Py), 6.82 (s, 6H, Ar), 6.15 (s, 6H, Ar), 2.74 (s, 40H, CH_2+empty **1a**), 1.97 (s, 2H, Cp), 1.76 (s, 2H, Cp), 0.36 (s, 4H, COD), -0.56 (s, 4H, COD), -1.13 (d, J = 7.8 Hz, 4H, COD), -1.39 (s, 3H, CH_3). **DOSY** (D_2O 298 K): log D = -9.73 m^2/s.

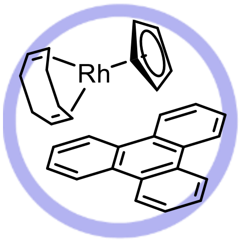

M = Pt(en)

1a•3•6

After encapsulation according to standard procedure, co-encapsulation was observed in 84% according to ^1H NMR. **^{1}H NMR** (300 MHz, D_2O) δ 9.62 (s br, 4H, Py), 9.17 (s br, 29H, Py+empty **1a**), 8.59 (s br, 18H, Py+empty **1a**), 8.12 (s br, 4H, Py), 6.98 (s, 6H, Ar), 6.35 (s, 6H, Ar), 2.90 (s, 40H, CH_2+empty **1a**), 2.09 (s, 5H, Cp), 0.92 (s, 4H, CH-COD), -0.36 (s, 4H, CH_2-COD), -0.94 (s, 4H, CH_2-COD). **DOSY** (D_2O, 298 K): log D = -9.735 m^2/s.

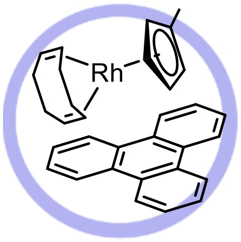

M = Pt(en)

1a•4•6

After encapsulation according to standard procedure, co-encapsulation was observed in 74% according to ^1H NMR. **^{1}H NMR** (500 MHz, D_2O) δ 9.50 (s, 6H, Py), 9.16 (s, 6H, Py), 9.05 (s br, 20H, Py+empty **1a**), 8.95 (s, 6H, Py), 8.50 (s, 6H, Py), 8.46 (s br, 15H, Py+empty **1a**), 8.00 (s, 6H, Py), 6.97 – 6.77 (m, 6H, Ar), 6.18 (s, 6H, Ar), 2.79 (d, J = 25.7 Hz, 32H, CH_2+empty **1a**), 1.93 (s, 2H, Cp), 1.86 (s, 2H, Cp), 0.54 (s, 4H, COD), -0.38 (s, 4H, COD), -0.98 (d, J = 7.4 Hz, 4H, COD), -1.52 (s, 3H, CH_3). **DOSY** (D_2O, 298 K): log D = -9.816 m^2/s.

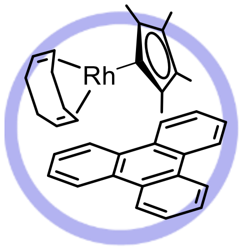

M = Pt(en)

1a•5•6

After encapsulation according to standard procedure, co-encapsulation was observed in 21% according to ^{1}H NMR. **^{1}H NMR** (500 MHz, D_2O) δ 9.65 (s, 6H Py), 9.25 (s, 8H Py), 9.21 (s, 16H Py), 9.14 (s, 11H Py), 8.68 (s, 14H Py+empty **1a**), 8.62 (s, 6H Py), 8.02 (s, 6H Py), 7.05 – 6.84 (m, 6H Ar), 6.13 (s, 6H Ar), 2.92 (s, 21H CH_2), 2.88 (s, 17H CH_2), 1.66 (s br, 4H COD-CH), 1.36 (s br, 4H COD-CH_2), 1.10 (s, 1H Cp-H), 0.88 (s, 4H COD-CH_2), -1.70 (s, 6H Cp-Me), -1.72 (s, 6H Cp-Me). **DOSY** (D_2O, 298 K): log D = -9.731 m^2/s.

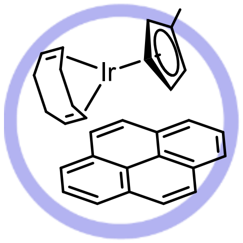

M = Pt(en)

1a•2•7

After encapsulation according to standard procedure, co-encapsulation was observed in 92% according to ^{1}H NMR. **^{1}H NMR** (400 MHz, D_2O) δ 10.26 – 7.66 (m, 52H), 6.60 (t, J = 7.7 Hz, 2H, Ar), 6.42 (d, J = 7.7 Hz, 4H, Ar), 6.12 (s, 4H, Ar), 2.88 (s, 25H, Pt(en)), 2.09 (s, 2H, Cp-H), 1.42 (s, 2H, Cp-H), 0.37 (s, 4H, COD), -0.53 (s, 4H, COD), -1.05 (d, J = 8.5 Hz, 4H, COD), -1.13 (s, 3H, Cp-CH_3). **DOSY** (D_2O, 298 K): log D = -9.756 m^2/s.

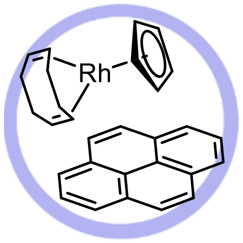

M = Pt(en)

1a•3•7

After encapsulation according to standard procedure, co-encapsulation was observed in 94% according to ^{1}H NMR. **^{1}H NMR** (400 MHz, D_2O) δ 9.91 – 7.76 (m, 51H, broad Py peaks), 6.64 (t, J = 7.6 Hz, 2H, Ar), 6.41 (d, J = 7.6 Hz, 2H, Ar), 6.16 (s, 2H, Ar), 2.88 (s, 25H, Pt(en)), 1.97 (s, 5H, Cp), 0.76 (s, 4H, COD), -0.58 (s, 4H, COD), -1.11 (d, J = 8.8 Hz, 2H, COD). **DOSY** (D_2O, 298 K): log D = -9.724 m^2/s.

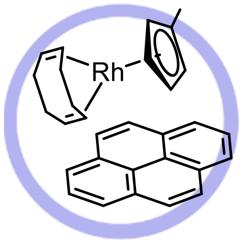

M = Pt(en)

1a•4•7

After encapsulation according to standard procedure, co-encapsulation was observed in 89% according to ^{1}H NMR. **^{1}H NMR** (400 MHz, D_2O) δ 10.12 – 7.74 (m, 54H), 6.61 (t, J = 7.5 Hz, 2H, Ar), 6.41 (d, J = 7.7 Hz, 4H, Ar), 6.13 (s, 4H, Ar), 2.88 (s, 27H, Pt(en)), 2.02 (s, 2H, Cp-H), 1.52 (s, 2H, Cp-H), 0.51 (s, 4H, COD), -0.41 (s, 4H, COD), -0.95 (d, J = 8.5 Hz, 4H, COD), -1.30 (s, 3H, Cp-CH_3). **DOSY** (D_2O, 298 K): log D = -9.749 m^2/s.

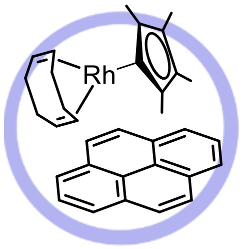

M = Pt(en)

1a•5•7

After encapsulation according to standard procedure, co-encapsulation was observed in 37% according to ^{1}H NMR. **^{1}H NMR** (300 MHz, D_2O) δ 10.23 – 7.59 (m, 74H, Py+empty **1a**), 6.51 (d, J = 7.7 Hz, 2H, Ar), 6.29 (d, J = 7.7 Hz, 4H, Ar), 5.95 (s, 4H, Ar), 3.18 – 2.65 (m, 33H, Pt(en)), 1.62 (d, J = 8.7 Hz, 4H, cod), 1.31 (s, 4H, cod), 0.80 (s, 4H, cod), 0.75 (s, 1H, Cp), -1.57 (s, 6H, CH_3), -1.91 (s, 6H, CH_3). **DOSY** (D_2O, 298 K): log D = -9.763 m^2/s.

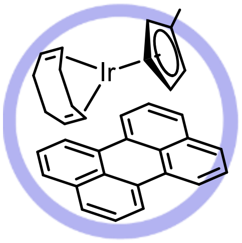

M = Pt(en)

1a•2•8

After encapsulation according to standard procedure, co-encapsulation was observed in 47% according to ^{1}H NMR. **^{1}H NMR** (300 MHz, D_2O) δ 9.91 – 7.80 (m, 103H, Py+empty **1a**), 6.62 (d, *J* = 8.2 Hz, 4H, Ar), 6.14 (d, *J* = 7.7 Hz, 4H, Ar), 5.77 (t, *J* = 7.7 Hz, 4H, Ar), 2.91 (s, 53H, Pt(en)+empty **1a**), 2.29 (s, 2H, Cp), 1.96 (s, 2H, Cp), 0.75 (s, 4H cod), -0.31 (s, 4H, cod), -0.91 (s, 4H, cod), -1.15 (s, 3H, CH_3). **DOSY** (D_2O, 298 K): log D = -9.529 m^2/s.

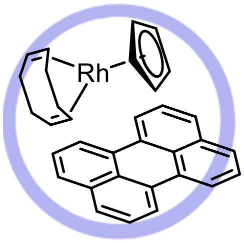

M = Pt(en)

1a•3•8

After encapsulation according to standard procedure, co-encapsulation was observed in 40% according to ^{1}H NMR. **^{1}H NMR** (400 MHz, D_2O) δ 9.75 – 7.84 (m, 121H), 6.61 (d, *J* = 8.1 Hz, 4H, Ar), 6.12 (d, *J* = 7.6 Hz, 4H, Ar), 5.74 (t, *J* = 7.8 Hz, 4H, Ar), 2.86 (s, 67H, Pt(en)), 2.12 (s, 5H, Cp-H), 1.10 (s, 4H, COD), -0.27 (s, 4H, COD), -0.89 (s, 4H, COD). **DOSY** (D_2O, 298 K): log D = -9.717 m^2/s.

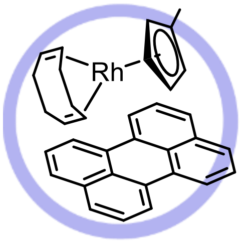

M = Pt(en)

1a•4•8

After encapsulation according to standard procedure, co-encapsulation was observed in 61% according to ^{1}H NMR. **^{1}H NMR** (400 MHz, D_2O) δ 9.95 – 7.72 (m, 78H, Py+empty **1a**), 6.63 (d, *J* = 8.3 Hz, 4H, Ar), 6.14 (d, *J* = 7.7 Hz, 4H, Ar), 5.77 (s, 4H, Ar), 2.91 (s, 39H, Pt(en)+ empty **1a**), 2.23 (s, 2H, Cp), 2.04 (s, 2H, Cp), 0.90 (s, 4H, cod), -0.16 (s, 4H, cod), -0.79 (s, 4H, cod), -1.32 (s, 3H, CH_3). **DOSY** (D_2O, 298 K): log D = -9.717 m^2/s.

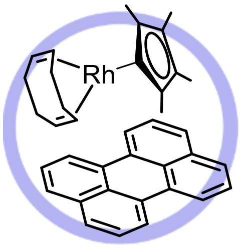

M = Pt(en)

1a•5•8

After encapsulation according to standard procedure, co-encapsulation was observed in 36% according to ^{1}H NMR. **^{1}H NMR** (400 MHz, D_2O) δ 10.14 – 7.65 (m, 134H, Py+empty **1a**), 6.50 (d, *J* = 7.9 Hz, 4H, Ar), 5.95 (d, *J* = 6.6 Hz, 4H, Ar), 5.72 (s, 4H, Ar), 2.91 (s, 69H, Pt(en)+empty **1a**), 1.16 (s, 4H, cod), 1.10 (s, 1H, Cp), 0.64 (s, 4H, cod), 0.06 (s, 4H, cod), -0.46 (s, 4H, cod), -1.69 (d, *J* = 9.5 Hz, 12H, CH_3). **DOSY** (D_2O, 298 K): log D = -9.742 m^2/s.

S4. Loss of symmetry of the metallocage

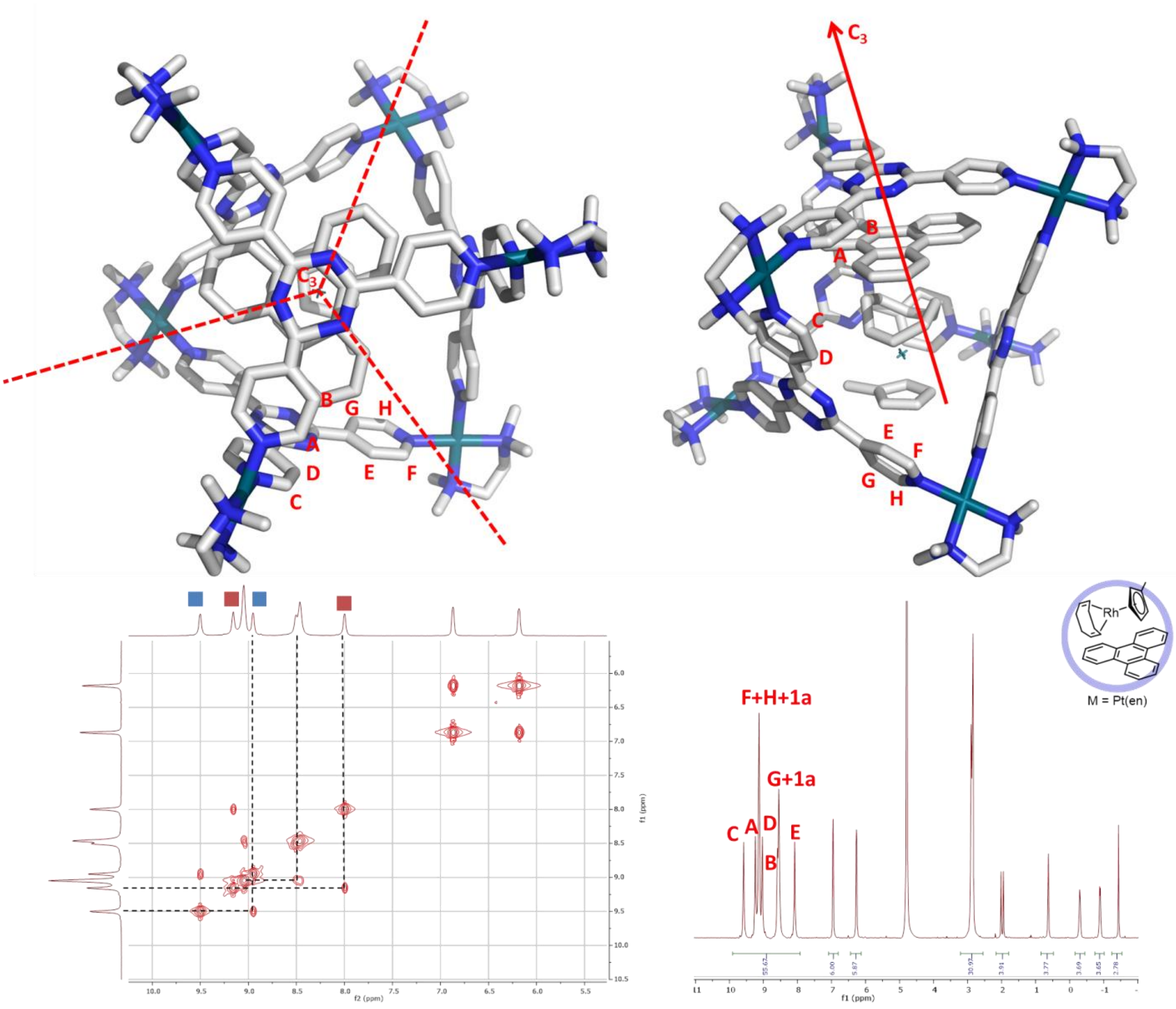

Figure 1: Modelled structure of **1c•4•6** (top) and the occurring loss of symmetry of the metallocage. Bottom figures indicate the annotation of the protons by $^{1}H^{1}H$-COSY (left) and ^{1}H NMR (right). For this, cage **1a** was used to prevent overlap of the Cp signals with the ethylendiamine ligand. This loss of symmetry is already observed previously in literature.[11]

S5. UV-Vis studies

a)

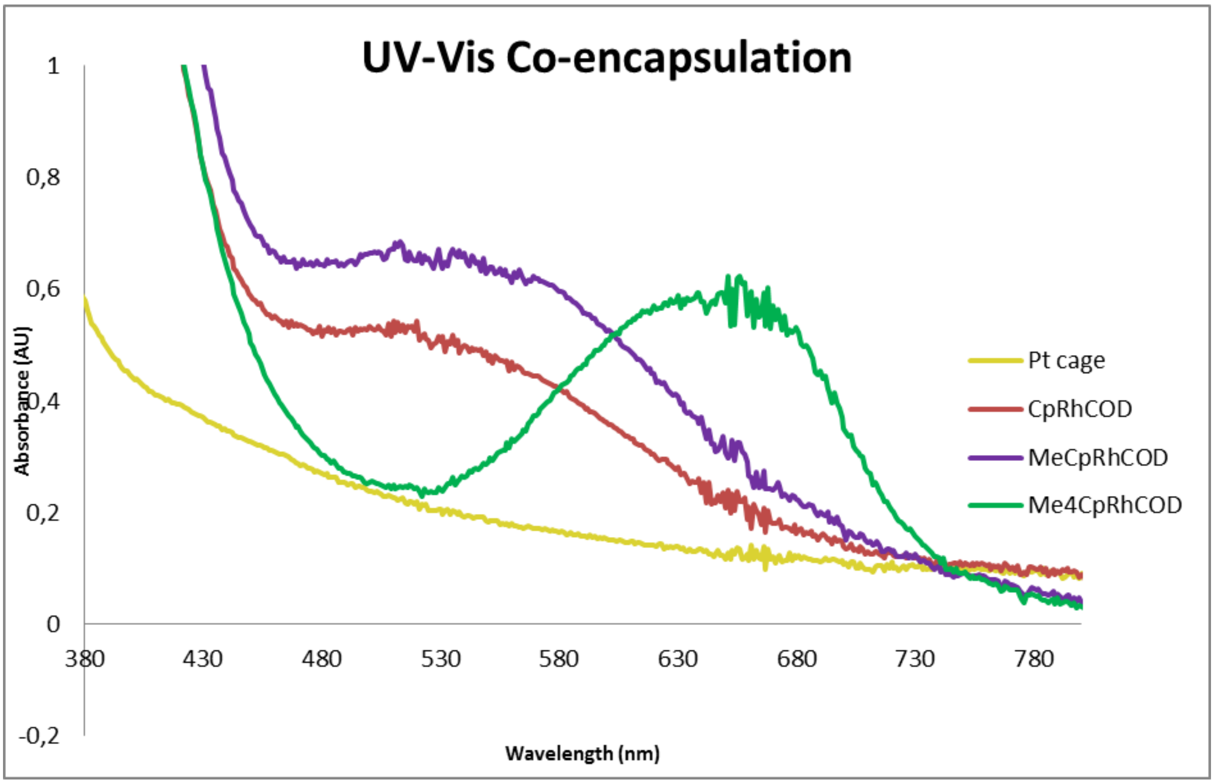

b)

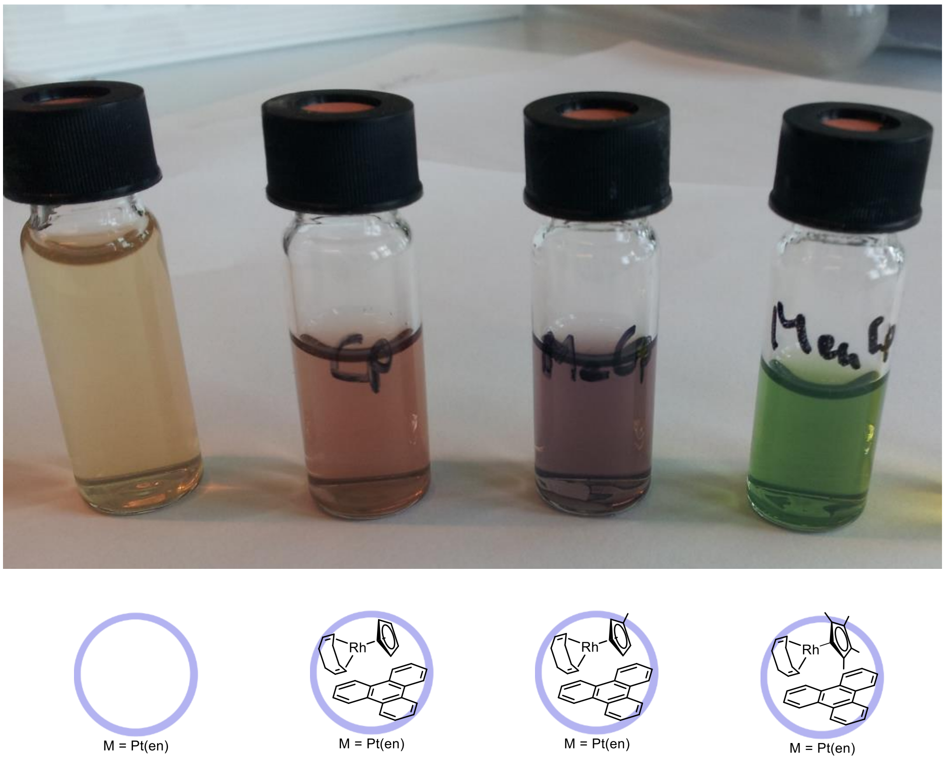

Figure 2: a) UV-Vis spectra (not normalized) of co-encapsulation inside the Pt_6L_4 sphere **1a** indicating the charge transfer band. b) photo visualizing the different colors of the charge transfer complexes

a)

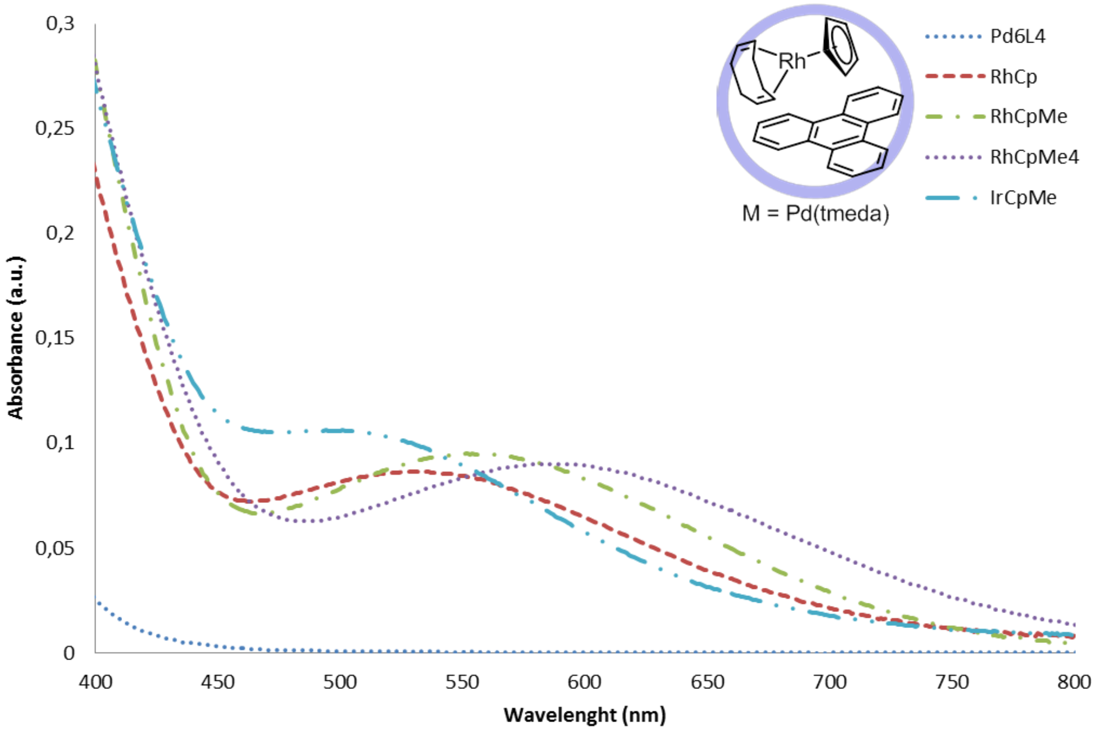

0,3
0,25
0,2
0,15
0,1
0,05
0
Absorbance (a.u.)
400
450
500
550
600
650
700
750
800
Wavelenght (nm)
Rh
M = Pd(tmeda)
Pd6L4
RhCp
RhCpMe
RhCpMe4
IrCpMe

b)

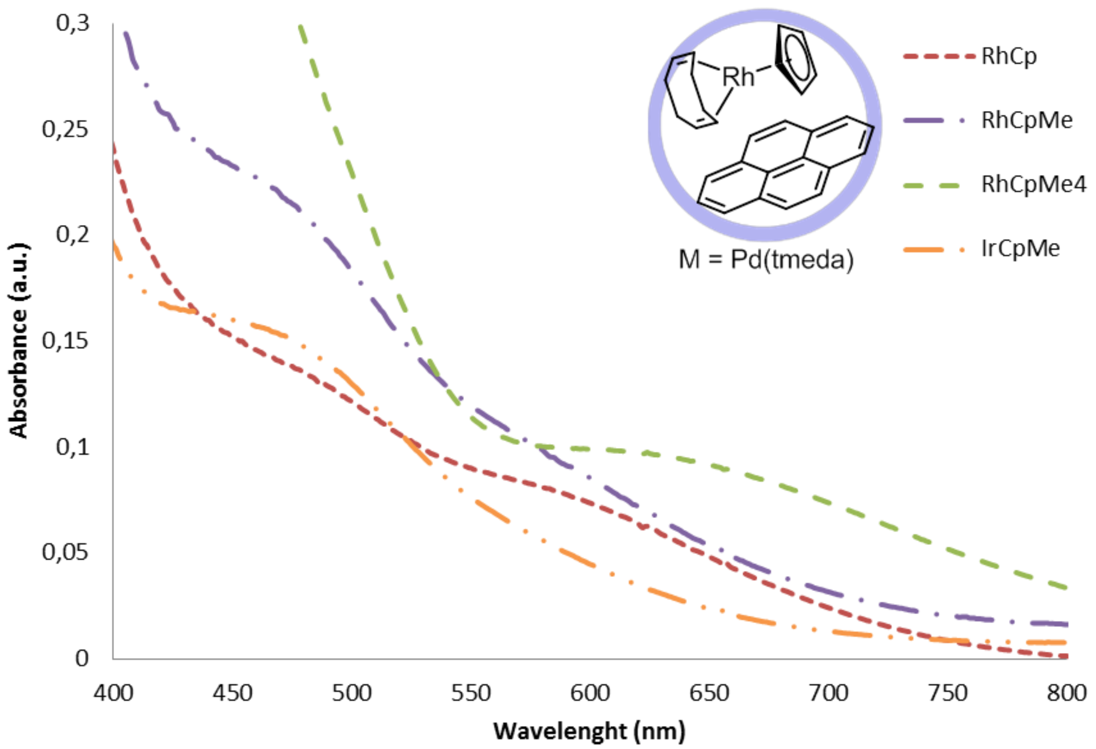

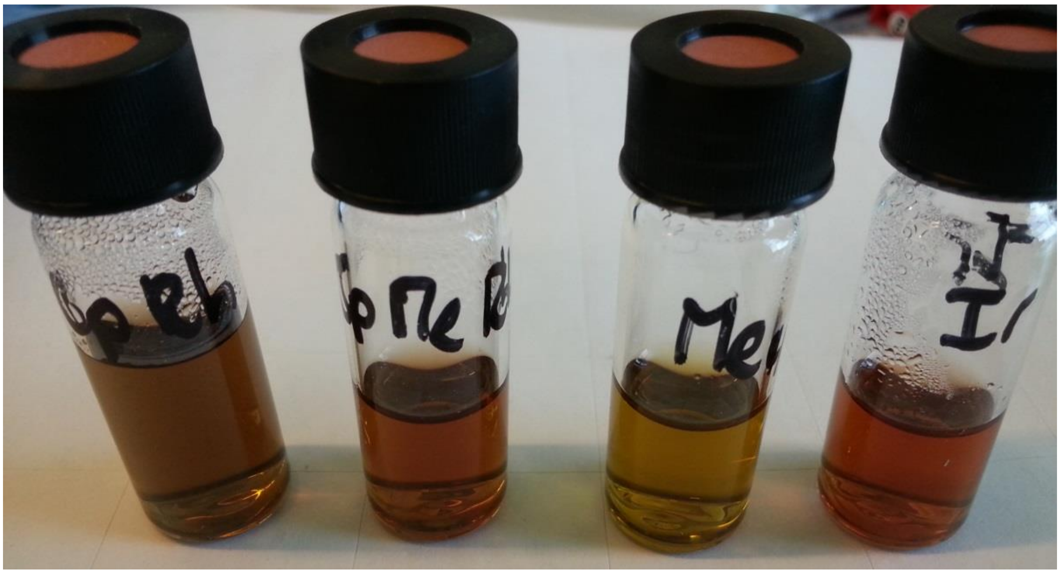

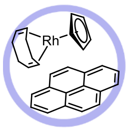

M = Pd(tmeda)

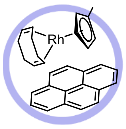

M = Pd(tmeda)

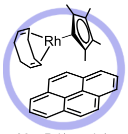

M = Pd(tmeda)

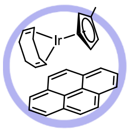

M = Pd(tmeda)

c)

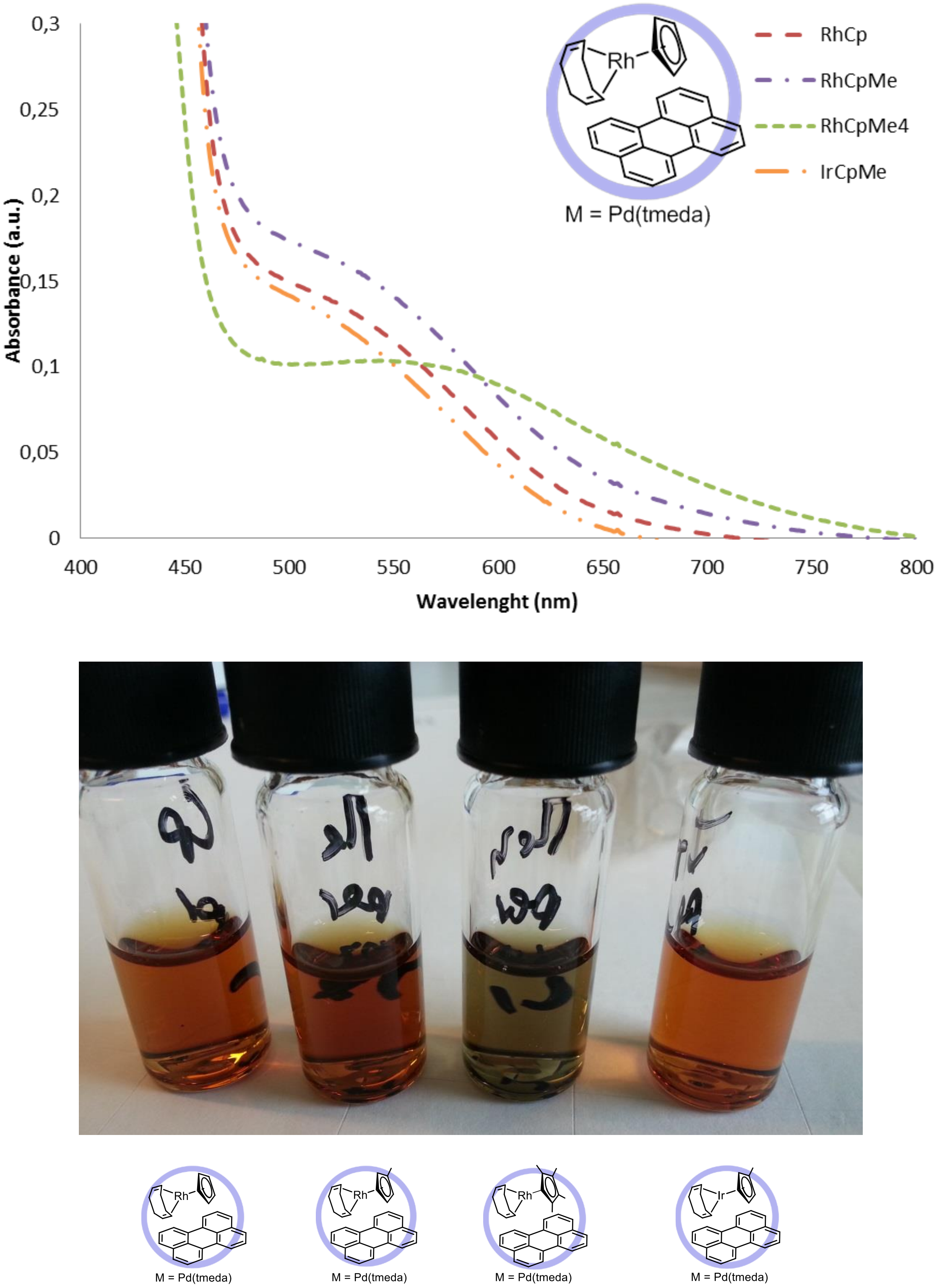

Figure 3: a) UV-Vis spectra of triphenylene co-encapsulated with Rh complexes. b) UV-Vis spectra of pyrene co-encapsulated with Rh complexes. c) UV-Vis spectra of perylene co-encapsulated with Rh complexes.

a)

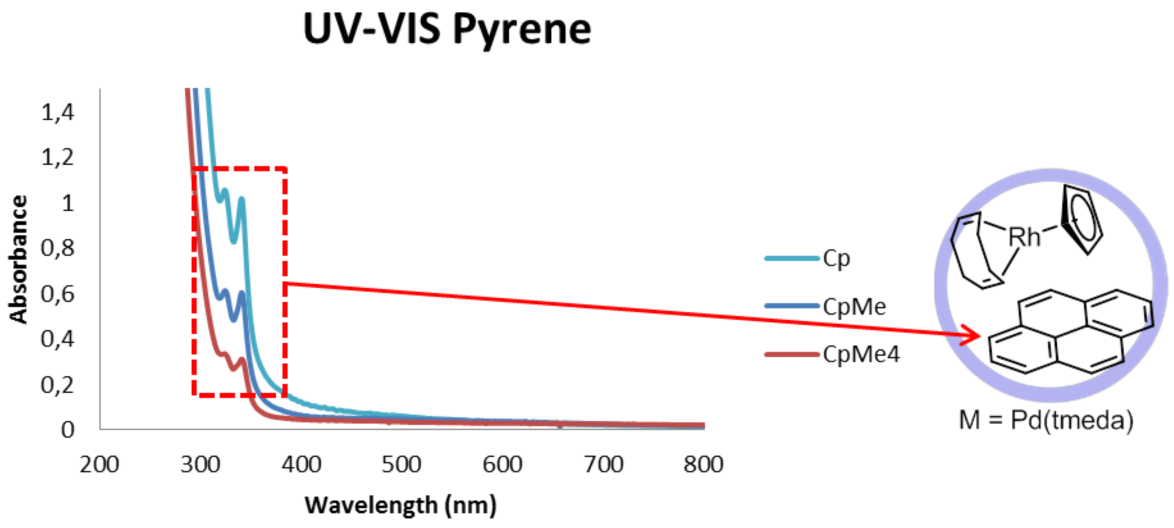

b)

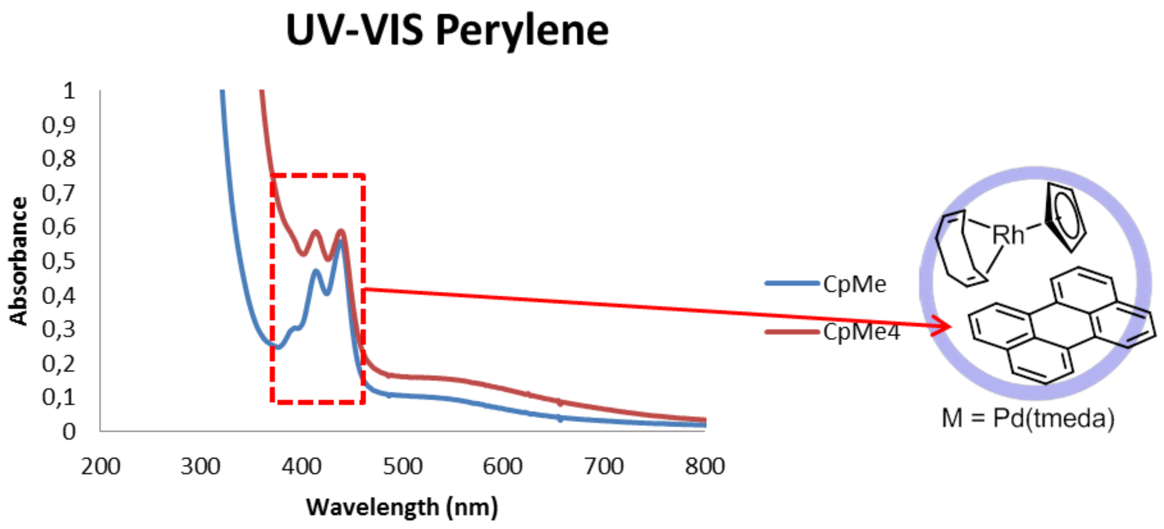

Figure 4: a) diluted UV-Vis spectra of pyrene co-encapsulated with Rh complexes highlighting the UV bands for pyrene corresponding to literature.[12] b) diluted UV-Vis spectra of perylene co-encapsulated with Rh complexes highlighting the UV bands for perylene.

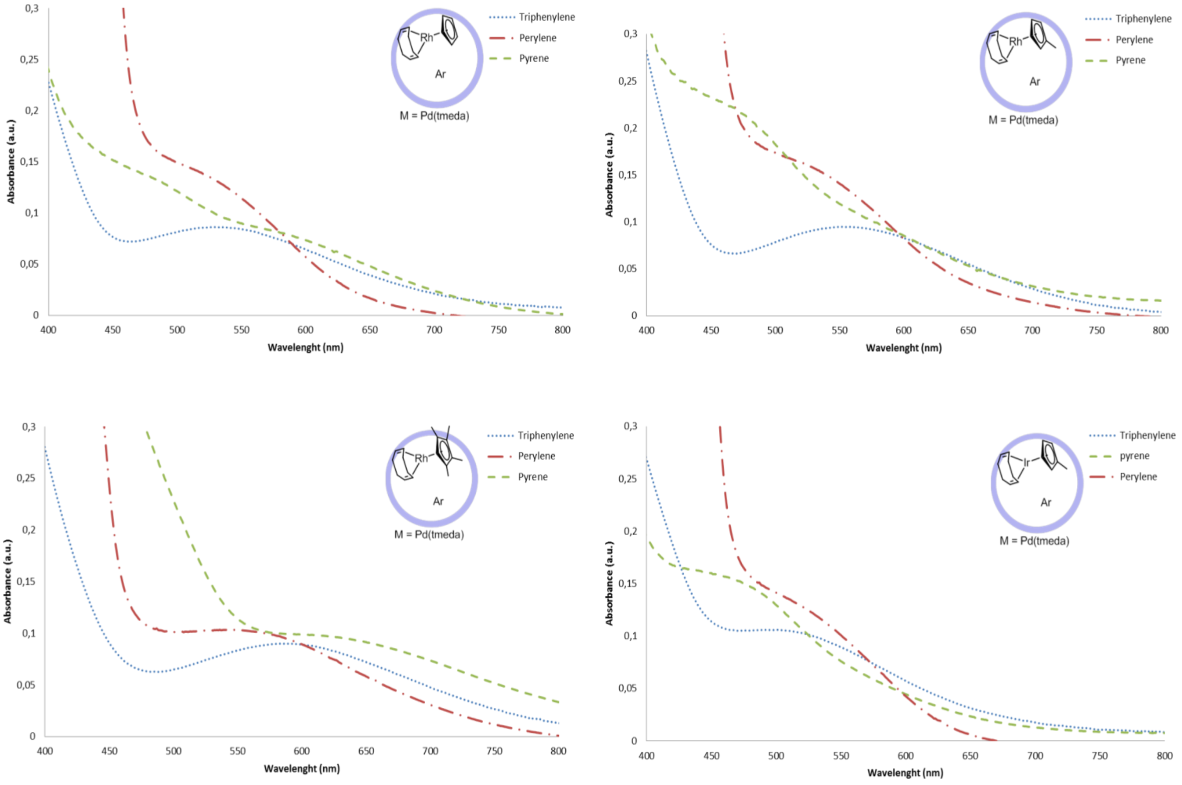

Figure 5: UV-vis spectra in which the same metal complex is plotted with different aromatic compound. This demonstrates that the different aromatic compounds shift the charge transfer band.

Determination of the CT band energies

The charge-transfer band energies were determined by a spectral line-shape analysis of the absorption spectra using Multipeak fitting package in IgorPro v6.36. The absorption spectra were converted from nanometer to wavenumber domain and the different absorption bands were approximated with Gaussian functions. Due to the large overlap of the different absorption bands we had to use up to three Gaussian functions to increase the accuracy of the determination of the CT band energy.

The fittings were most reliable in the case of triphenylene (**6**) where the CT band is well separated from the main absorption band. In the case of pyrene (**7**), the long wavelength absorption consists of two distinct bands, one located at ~450 nm and a weaker band at >600 nm. The band at ~450 nm did not exhibit significant changes in the peak position (variation < 15 nm) with the different metal complexes. Therefore the weaker lowest energy band was attributed to the CT absorption band. In the case of perylene (**8**), the main absorption of the chromophore overlaps with the CT band especially with RhCp, RhCpMe, and IrCpMe complexes. Therefore, it was necessary to fit part of the perylene spectrum to increase the accuracy in determination of the peak maxima of the CT band.

Table 1: Wavelengths (nm) and corresponding energies (eV) of the charge transfer (CT) bands obtained from fitting the curves.

Aromatic Guest / Metal donor	Pyrene (**7**)		Triphenylene (**6**)		Perylene (**8**)	
	λ_{CT} (nm)	E_{CT} (eV)	λ_{CT} (nm)	E_{CT} (eV)	λ_{CT} (nm)	E_{CT} (eV)
(CpMe)Ir (**2**)	609	2.04	496	2.50	520	2.38
$(CpMe_4)$Rh (**5**)	658	1.88	586	2.13	557	2.22
(CpMe)Rh (**4**)	612	2.03	555	2.23	517	2.40
(Cp)Rh (**3**)	617	2.01	530	2.34	497	2.50

S6. Electrochemistry

Cyclic voltammetry was performed on (close to) 1 mM solutions of the analyte in dichloromethane containing 0.1 M $n\mathrm{Bu_4NPF_6}$ as the supporting electrolyte. The voltammograms were recorded using a PGSTAT302N potentiostat (Metrohm/Autolab), a glassy carbon disk (1 mm diameter) as a working electrode, a glassy carbon rod as an auxiliary electrode and a leakless $Ag^{0/+}$ reference electrode (eDAQ ET069). To convert the potential values of the $Ag^{0/+}$ reference to $Fc^{0/+}$ a correction factor of –0.57 V was used as determined by cyclic voltammetry of 1 mM ferrocene in dichloromethane using the same reference electrode. Solution resistance was compensated to 90-95%.

Cyclic voltammetric data was fitted using DigiElch 7 (ElchSoft). For each species, voltammograms from 3 different scan rates (0.1/0.3/1.0 V/s) were fitted simultaneously.

Redox properties by digital simulation

The only model that could be properly fitted to all of the voltammograms is a quasi-reversible redox process followed by a reversible chemical transformation:

Redox: A ←→ B (E$_{ox}$; k$_s$; α) with $\alpha = 0.5$

Chemical: B ←→ C (k$_f$; k$_b$)

	E_{ox} [V] vs $Fc^{0/+}$	k_s [cm/s]	k_f	k_b
Ir(CpMe)(cod)	0.12	0.030	24000	7.2
Rh(Cp)(cod)	0.07	0.032	28000	9.2
Rh(CpMe)(cod)	0.02	0.035	25000	12
Rh($CpMe_4$)(cod)	-0.28	0.0051	0.52	0.055

Since the chemical follow-up reaction is almost non-existent for the $CpMe_4$ species, we ascribe the transformation to a change in Cp coordination, which is in this case most probably slowed down by increased $CpMe_4$ bulk compared to the Cp and CpMe species.

Cyclic voltammograms of Metal complexes

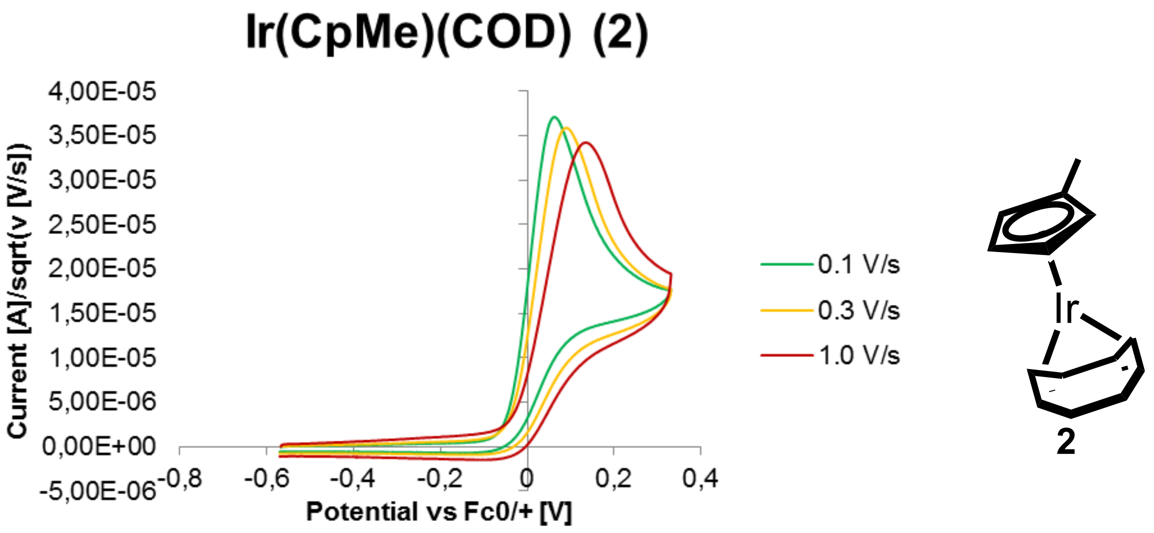

Concentration: 1.6 mM

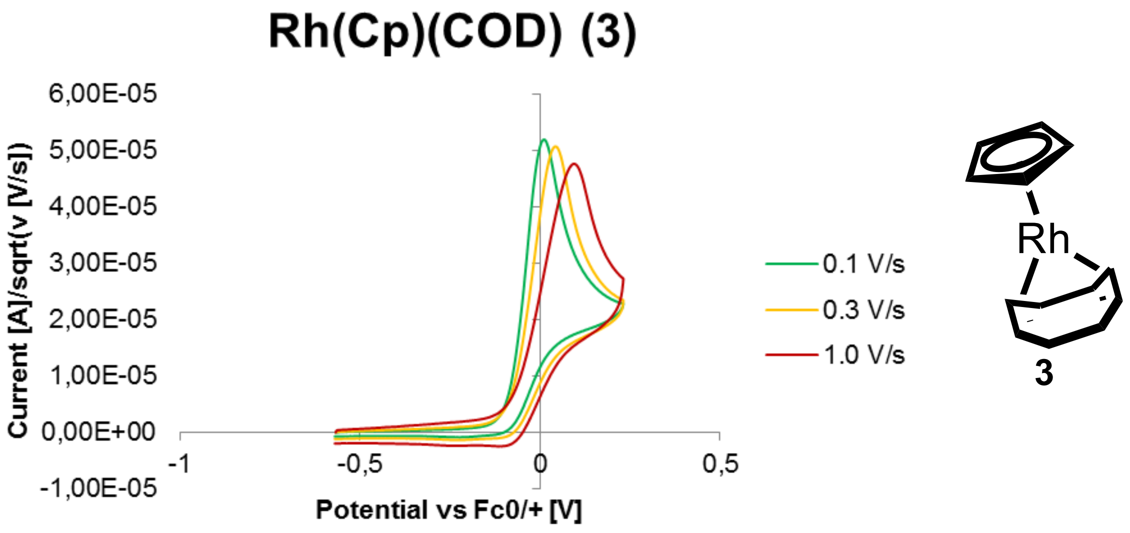

Concentration: 0.85 mM

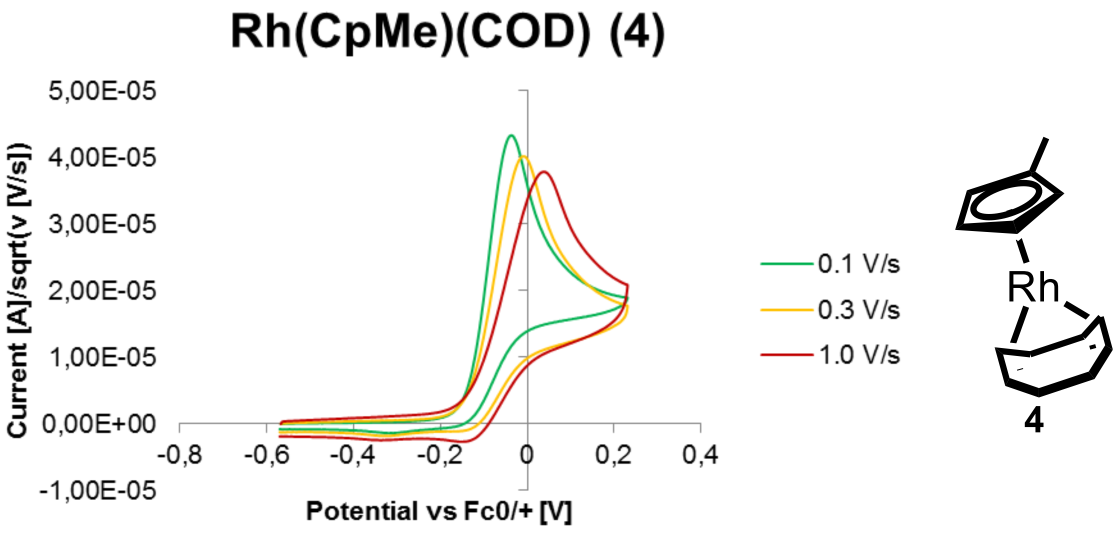

Concentration: 1.7 mM

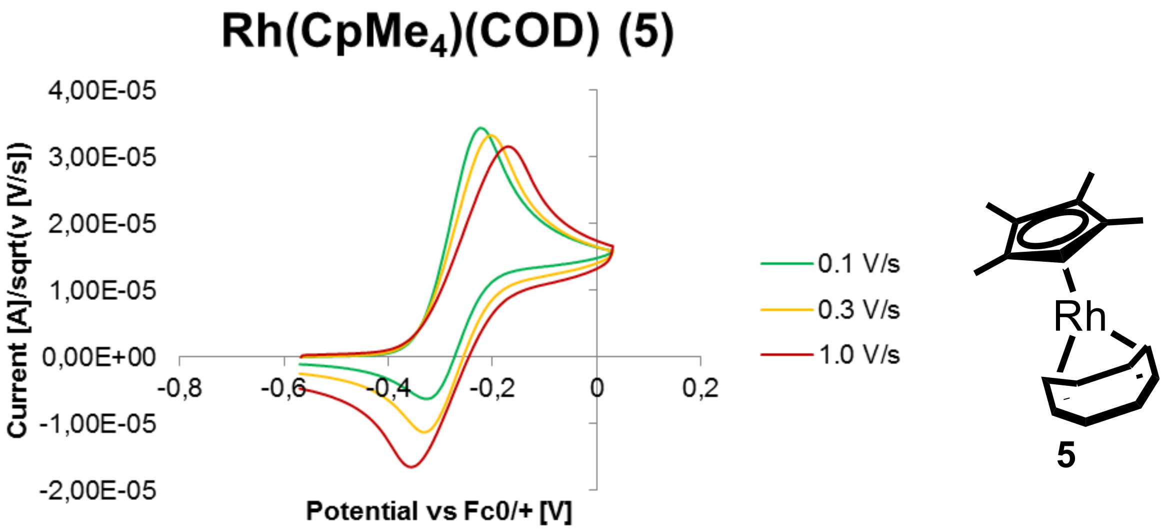

Concentration: 1.3 mM

S7. X-ray Crystal Structure of ternary complex 1b•5•6

Crystallographic diffraction data were measured on a Bruker APEX-II/CCD diffractometer equipped with a focusing mirror (Mo K*α* radiation *λ* = 0.71073 Å) with a cryostat system equipped with a N_2 generator (Japan Thermal Eng.). The crystals were removed from the solution, quickly attached to a loop of nylon fiber with antifreeze reagent (PVP, Hampton research), and mounted on a goniometer. The data collection was performed at 90 K. The structures were solved by direct methods (SHELXS-2014) and refined by full-matrix least-squares calculations (SHELXL-2014) on F^2. Hydrogen atoms were fixed at calculated positions and refined using a riding model.

Single crystals of **1b•2•6** were obtained by combining **1b** (15 *µ*mol, 53 mg), **2** (45 *µ*mol, 17 mg) and **6** (45 *µ*mol, 11 mg) in a vial with D_2O (1 mL). The suspension was then heated for 1 hour at 100 °C, cooled down and filtered over a syringe filter. The clear solution was left to stand in a NMR tube over a week to obtain single crystals.

Various needle crystals were tested, good diffraction data was obtained from a needle crystal of a (CpMe)Ir(cod)•triphenylene co-encapsulation complex. Although the Ir complex (**2**) and triphenylene (**6**) pair was disordered in three positions, the host-guest structure was clearly observed.

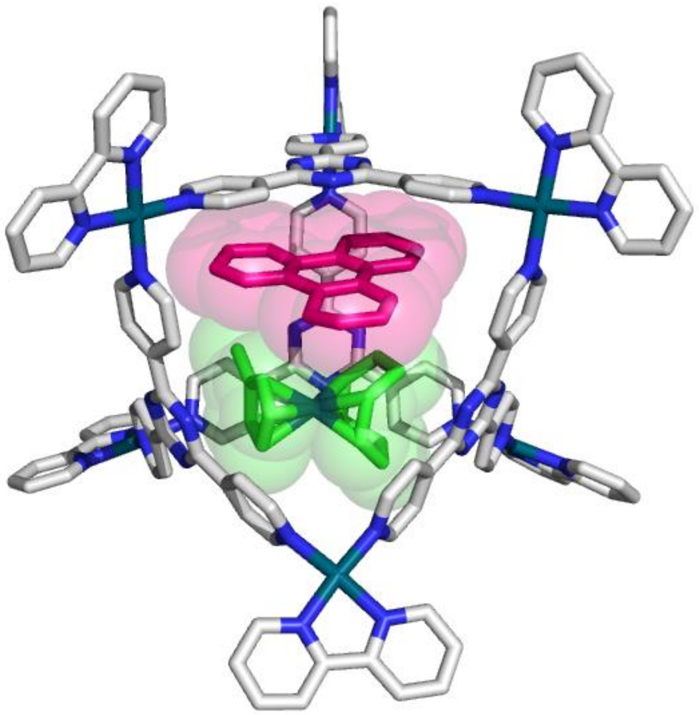

Figure 6: Crystal structure of ternary complex **1b•2•6**, (CpMe)Ir(cod)•triphenylene within the M_6L_4 cage.

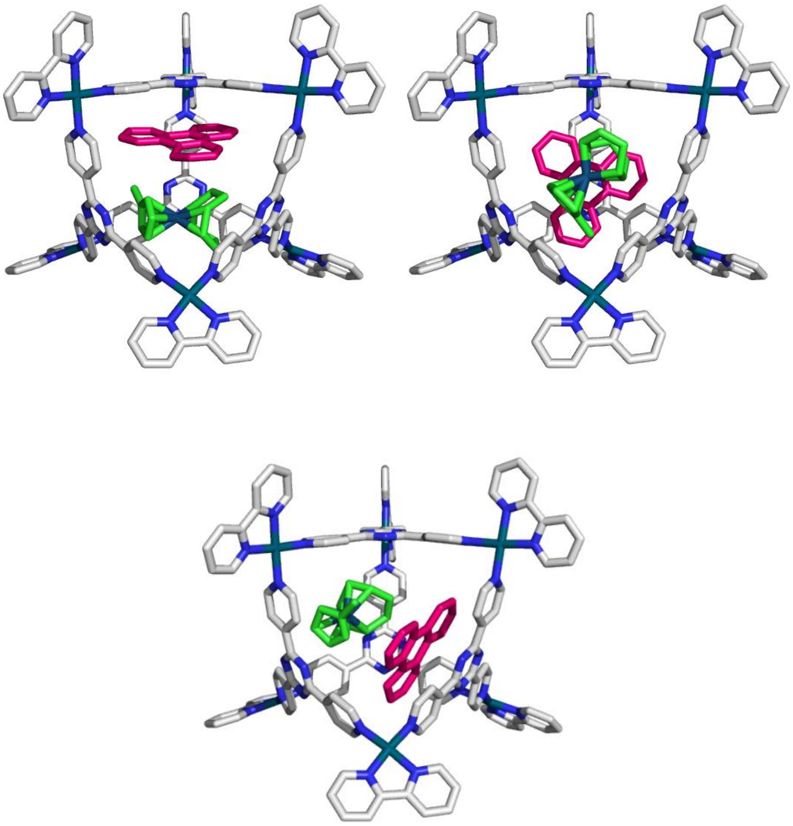

Figure 7: Disorder of guest pair in three positions (33.3% occupancy respectively).

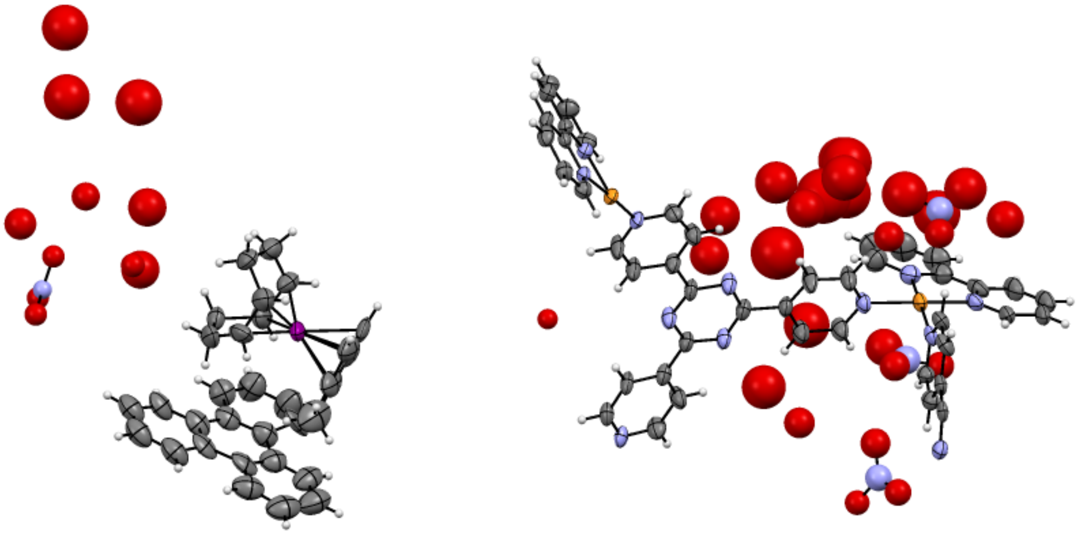

Figure 8: Thermal ellipsoids (50% probability) drawing of the crystal structure.

Table 2: Crystal data and structure refinement for p-3c1. (ternary complex **1b•2•6**)

CCDC reference number	1423147	
Identification code	p-3c1	
Empirical formula	C_{164} $H_{160.67}$ Ir $N_{46.50}$ O_{82} Pd_6	
Formula weight	4925.66	
Temperature	90(2) K	
Wavelength	0.71073 Å	
Crystal system	Trigonal	
Space group	$\bar{P}$;3c1	
Unit cell dimensions	a = 28.008(15) Å	α = 90 °
	b = 28.008(15) Å	β = 90 °
	c = 34.374(19) Å	γ = 120 °
Volume	23352(29) Å^3	
Z	4	
Density (calculated)	1.401 Mg•m^{-3}	
Absorption coefficient	1.110 mm^{-1}	
F_{000}	9917	
Crystal size	0.25 × 0.04 × 0.04 mm^3	
Theta range for data collection	1.68 to 28.45 °	
Index ranges	–37<=h<=36, –37<=k<=36, –43<=l<=44	
Reflections collected	242340	
Independent reflections	19049 [R_{int} = 0.1294]	
Completeness to θ = 28.45 °	96.8%	
Max. and min. transmission	0.9569 and 0.7688	
Refinement method	Full-matrix least-squares on F^2	
Data / restraints / parameters	19049 / 338 / 980	
Goodness-of-fit on F^2	1.141	
Final R indices [I>2sigma(I)]	R_1 = 0.1103, ωR_2 = 0.2619	

R indices (all data)	R_1 = 0.1805, ωR_2 = 0.3045
Largest diff. peak and hole	1.717 and –0.845 e.Å^{-3}

S8. Spectral data of compounds and co-encapsulation products

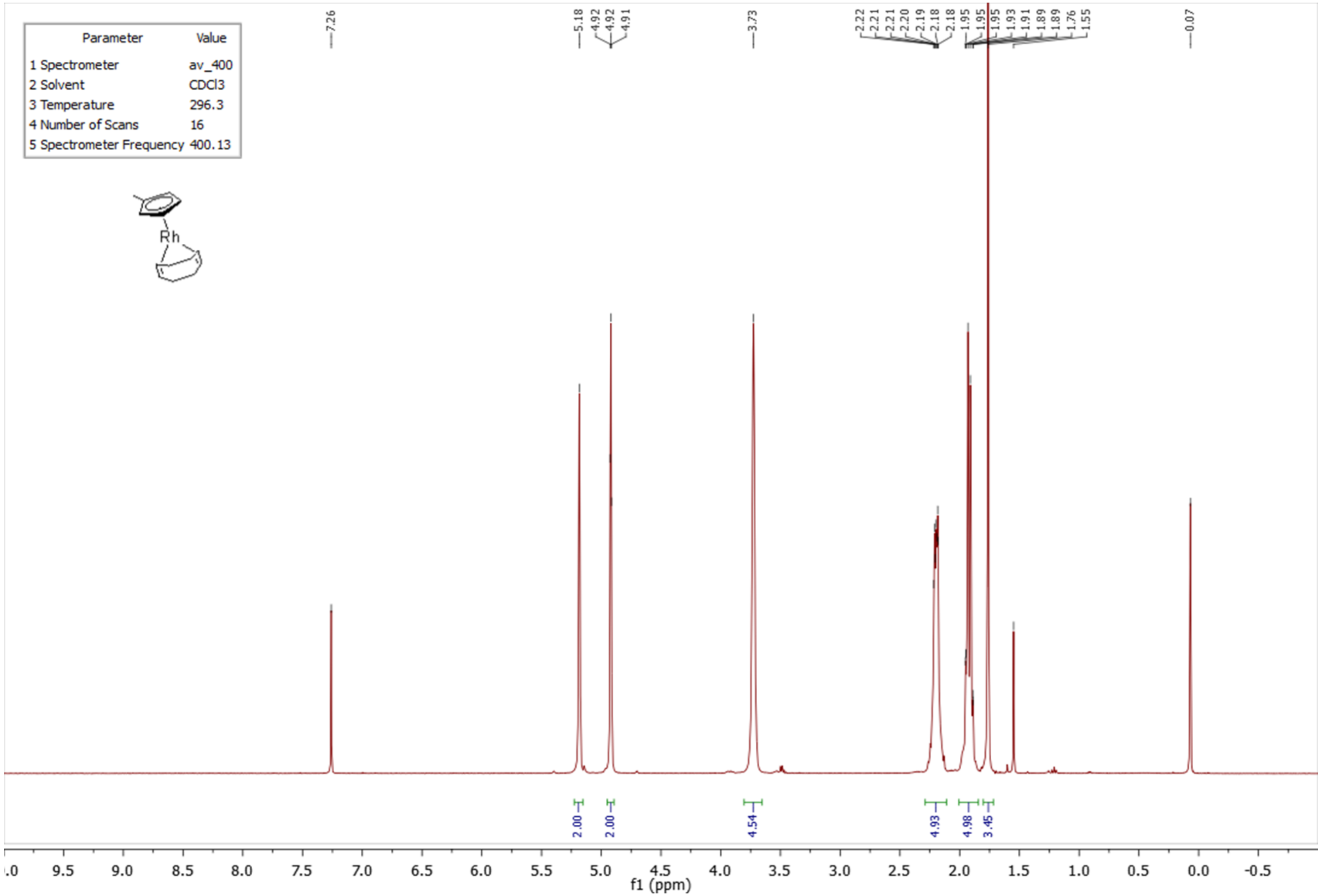

Figure 9: ^{1}H NMR spectrum (400 MHz) of (CpMe)Rh(cod) (**4**)

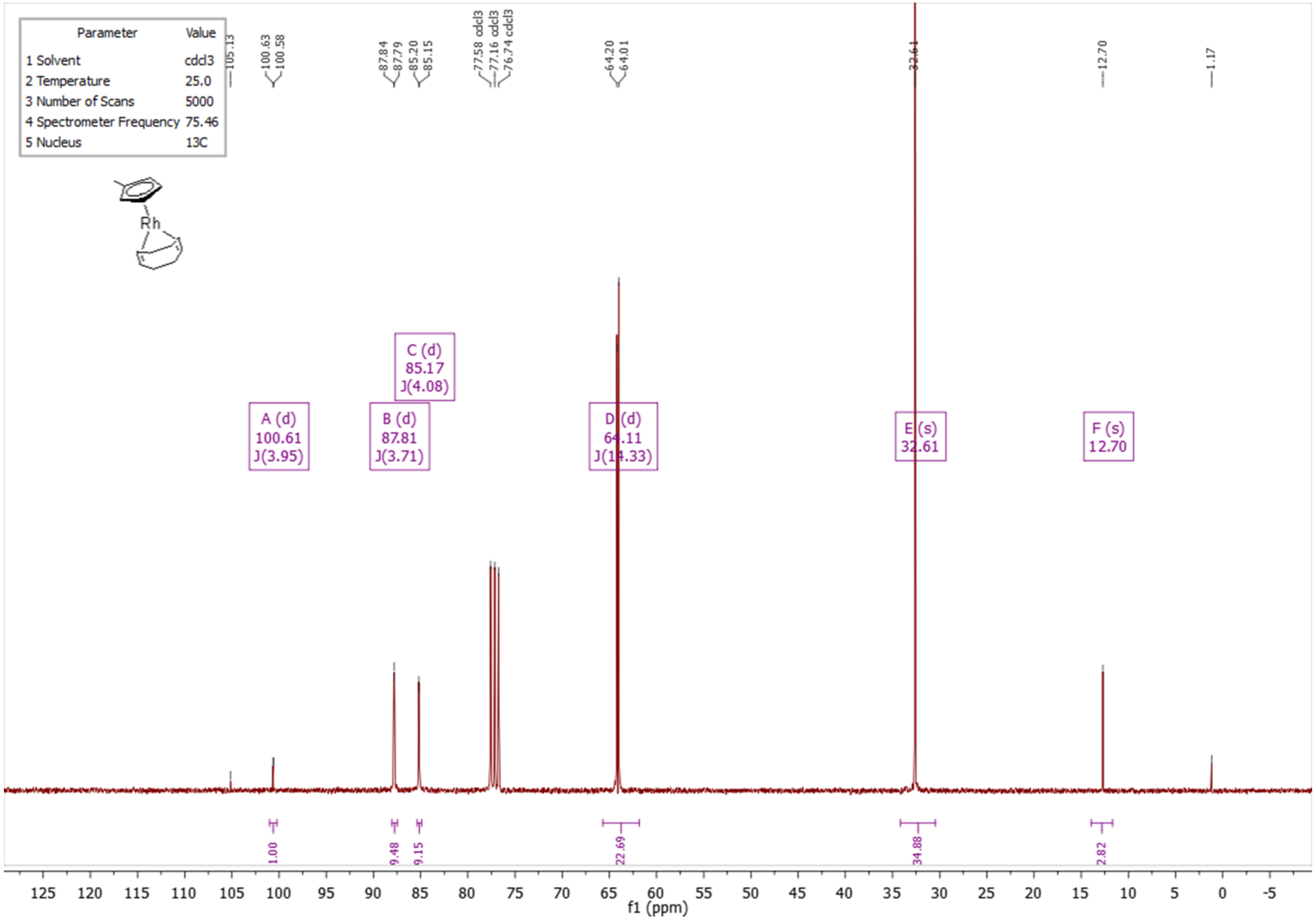

Figure 10: ^{13}C NMR spectrum (75 MHz) of (CpMe)Rh(cod) (**4**)

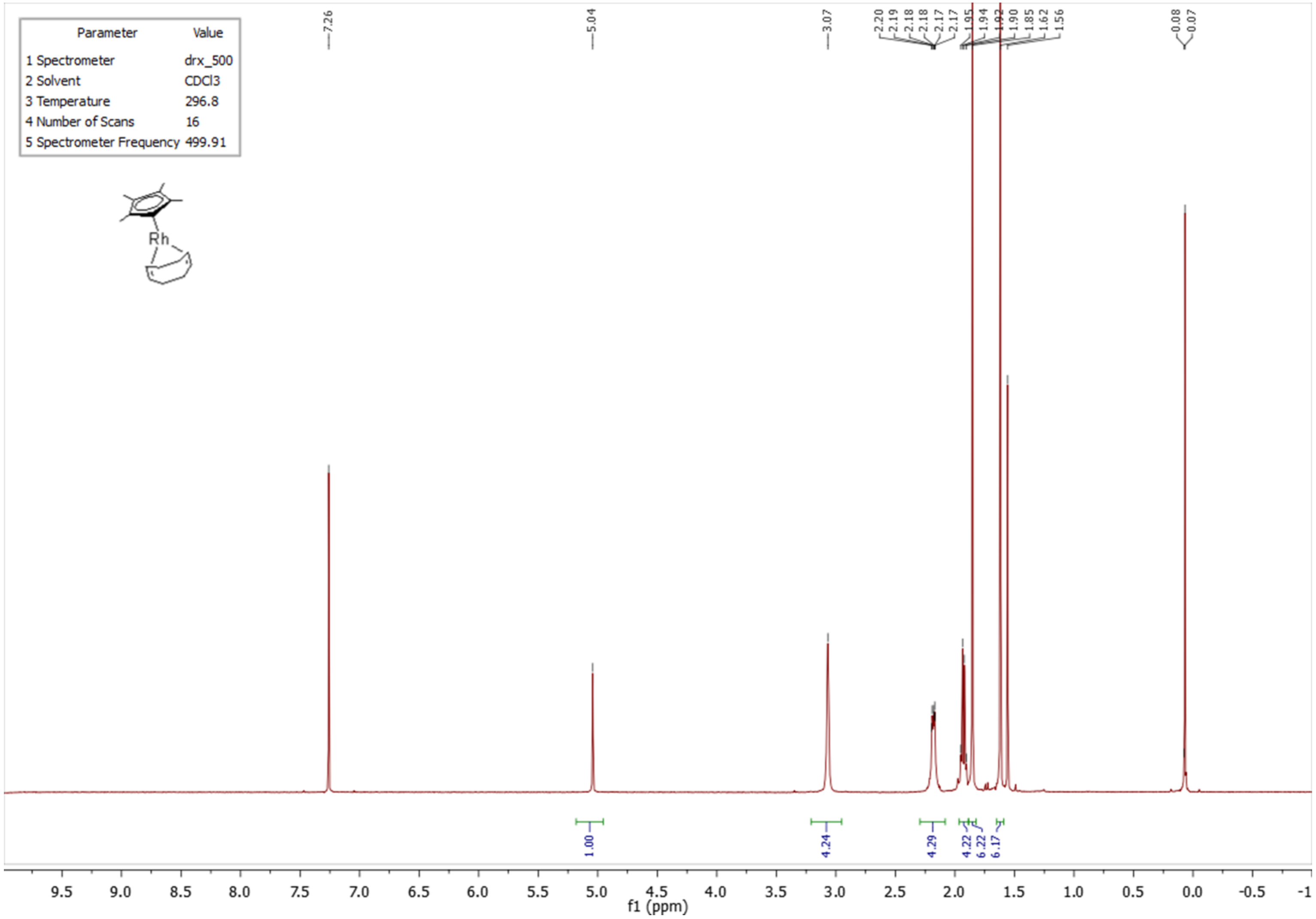

Figure 11: ^{1}H NMR spectrum (500 MHz) of (CpMe$_4$)Rh(cod) (**5**)

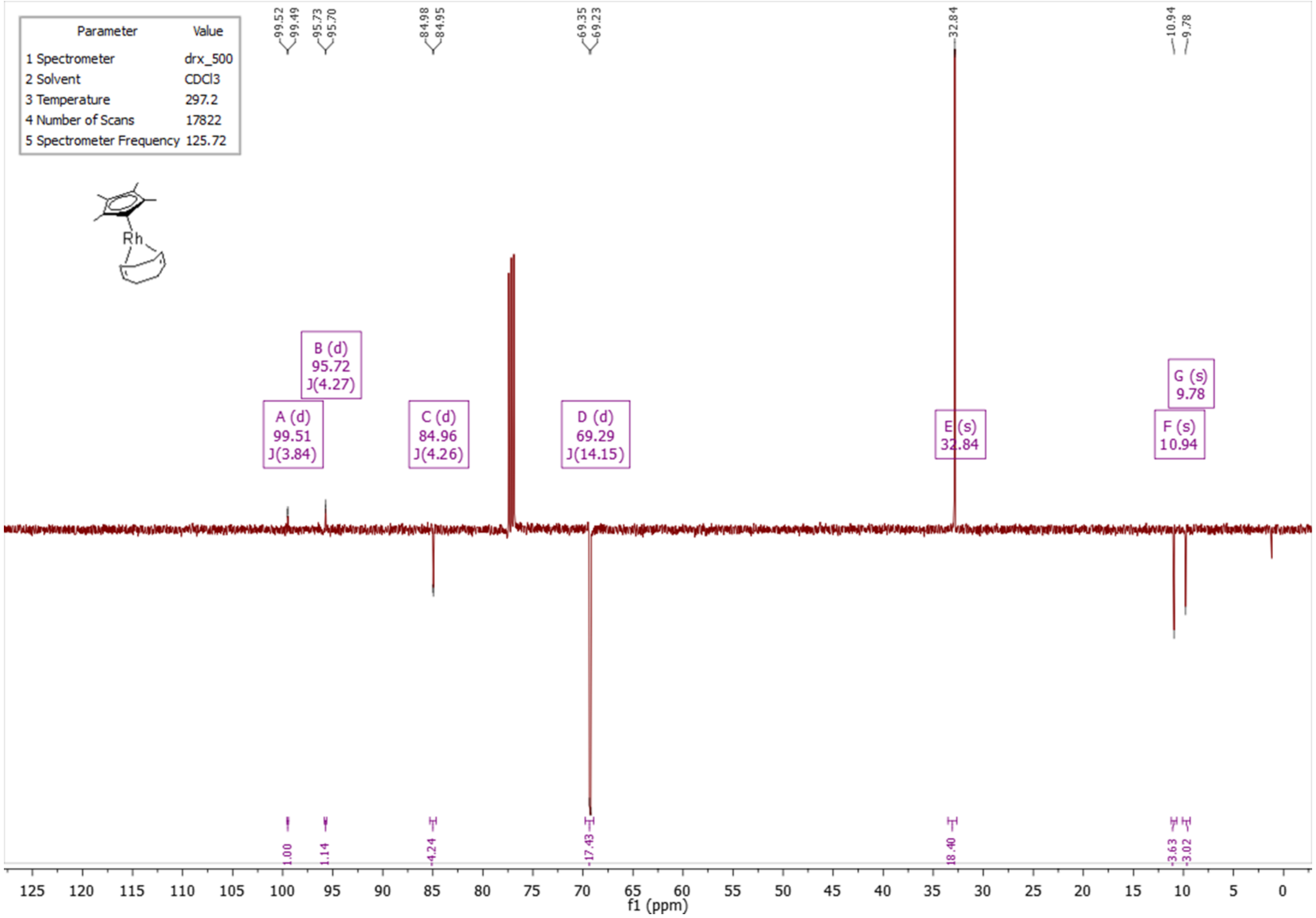

Figure 12: ^{13}C NMR (ATP) spectrum (125 MHz) of $(CpMe_4)Rh(cod)$ (**5**)

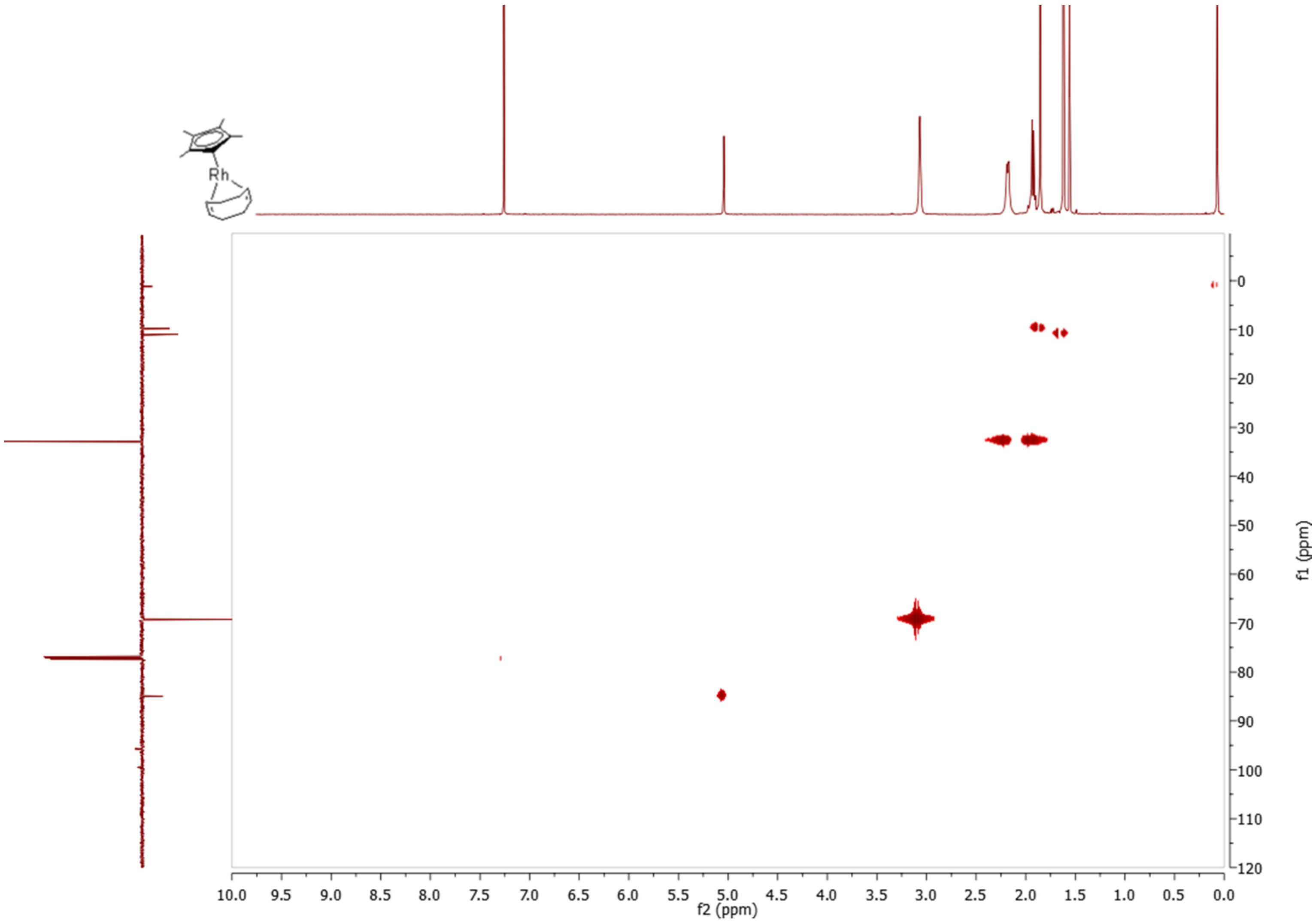

Figure 13: HSQC spectrum of $(CpMe_4)Rh(cod)$ (**5**)

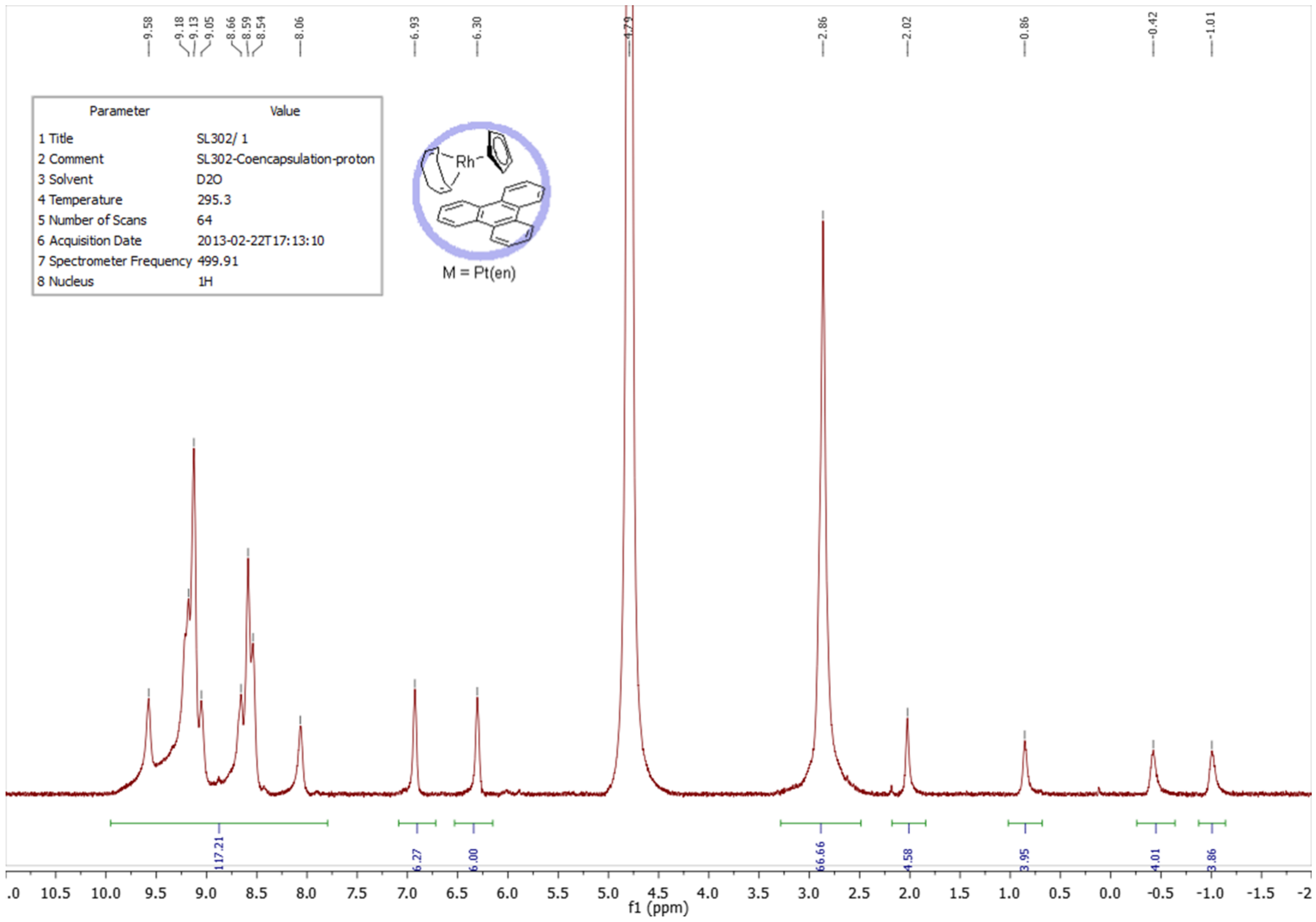

Figure 14: ^{1}H NMR spectrum (500 MHz) of **1a•3•6**

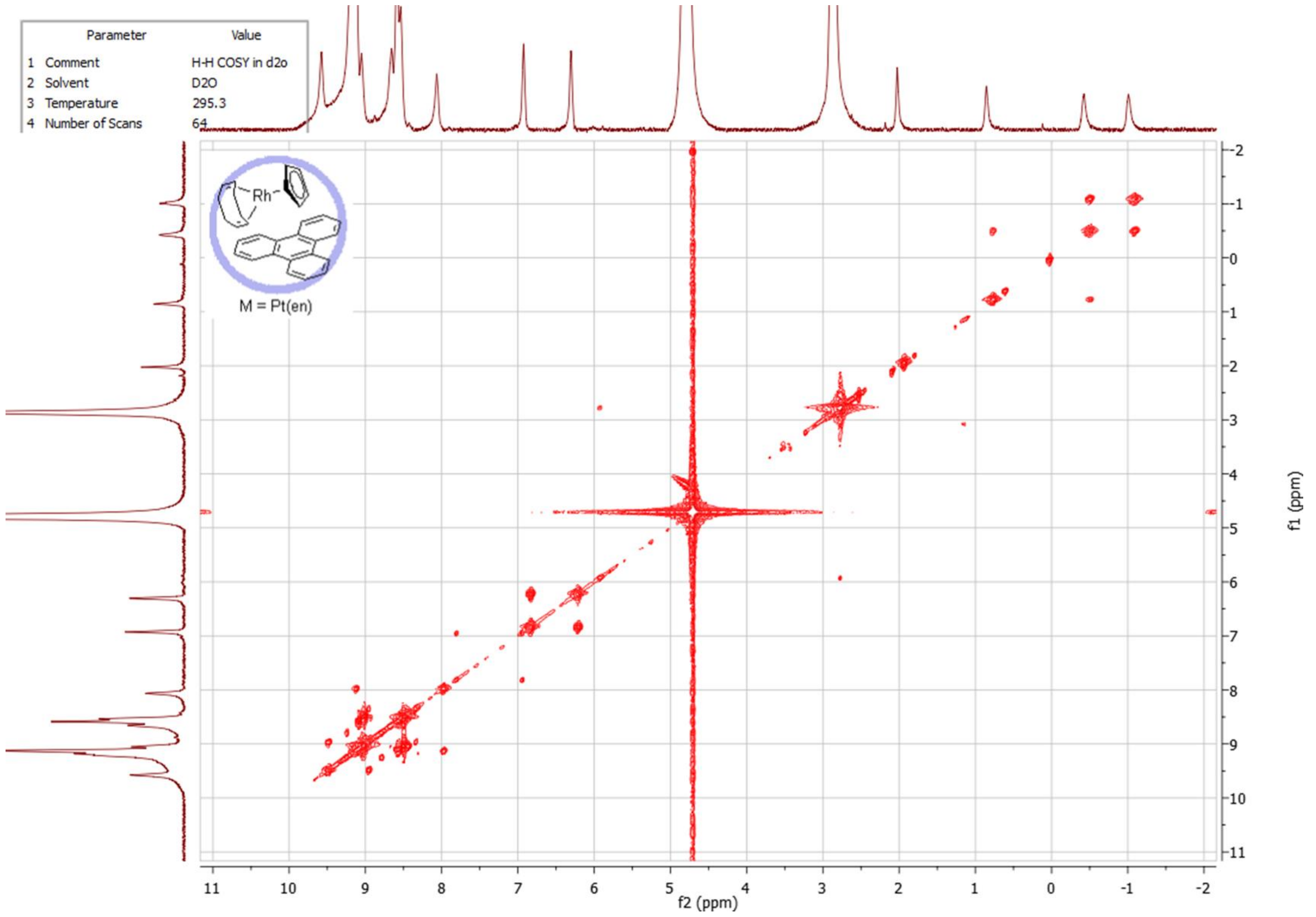

Figure 15: ^{1}H-^{1}H COSY spectrum of **1a•3•6**

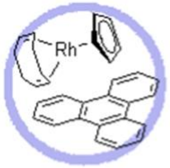

M = Pt(en)

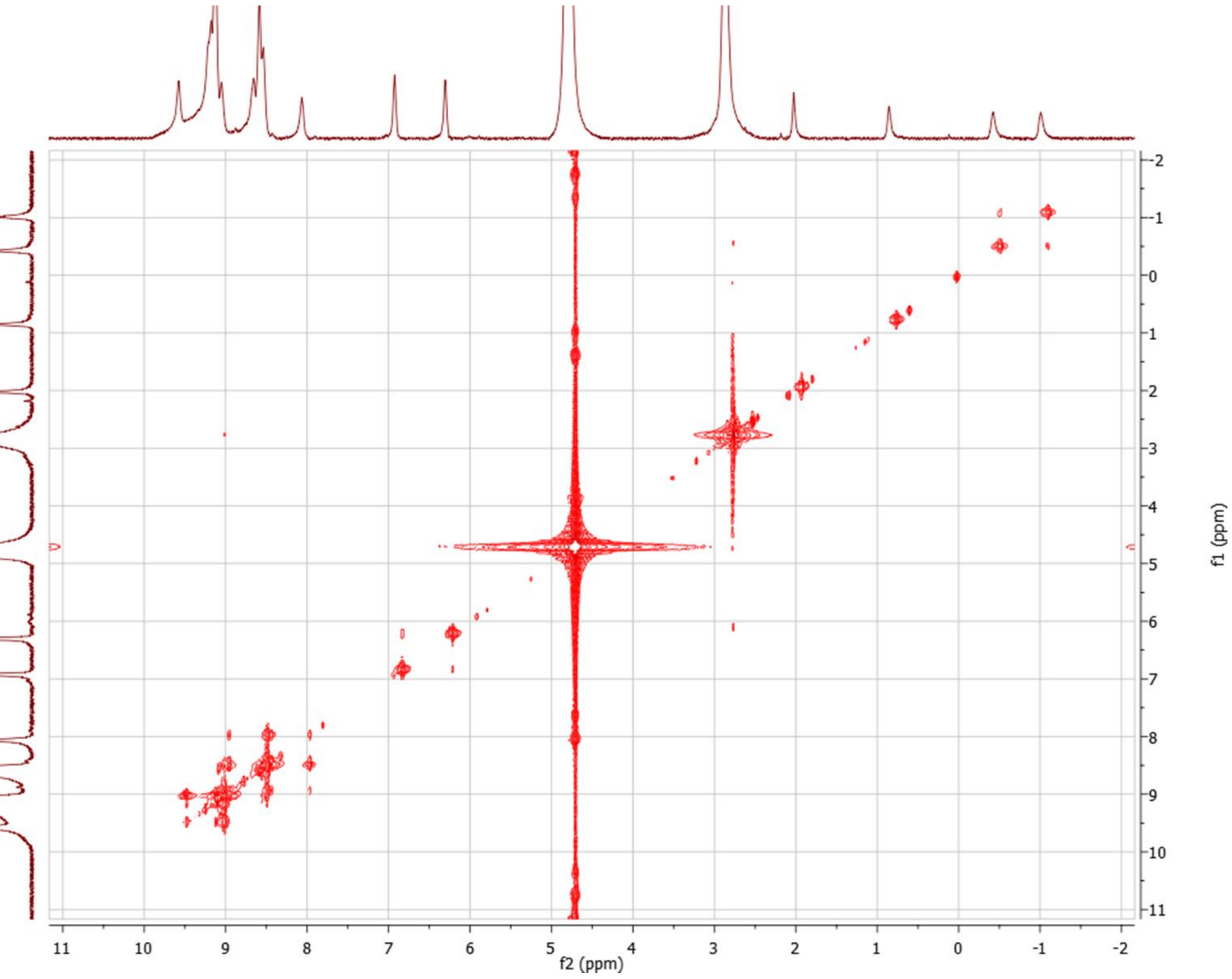

Figure 16: NOESY spectrum of **1a•3•6**

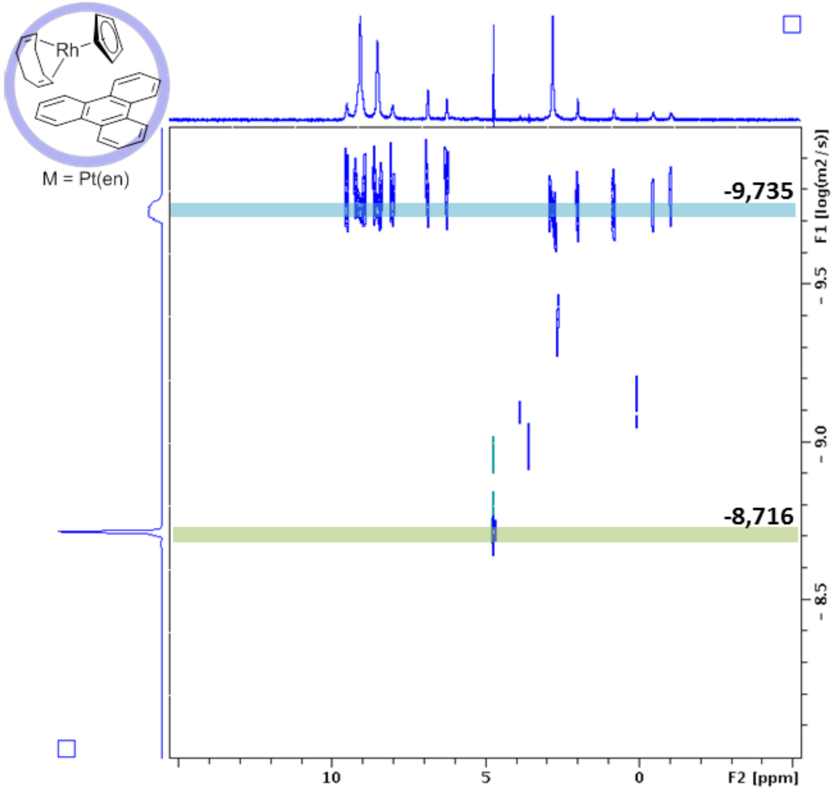

Figure 17: DOSY of **1a•3•6** at 25 °C

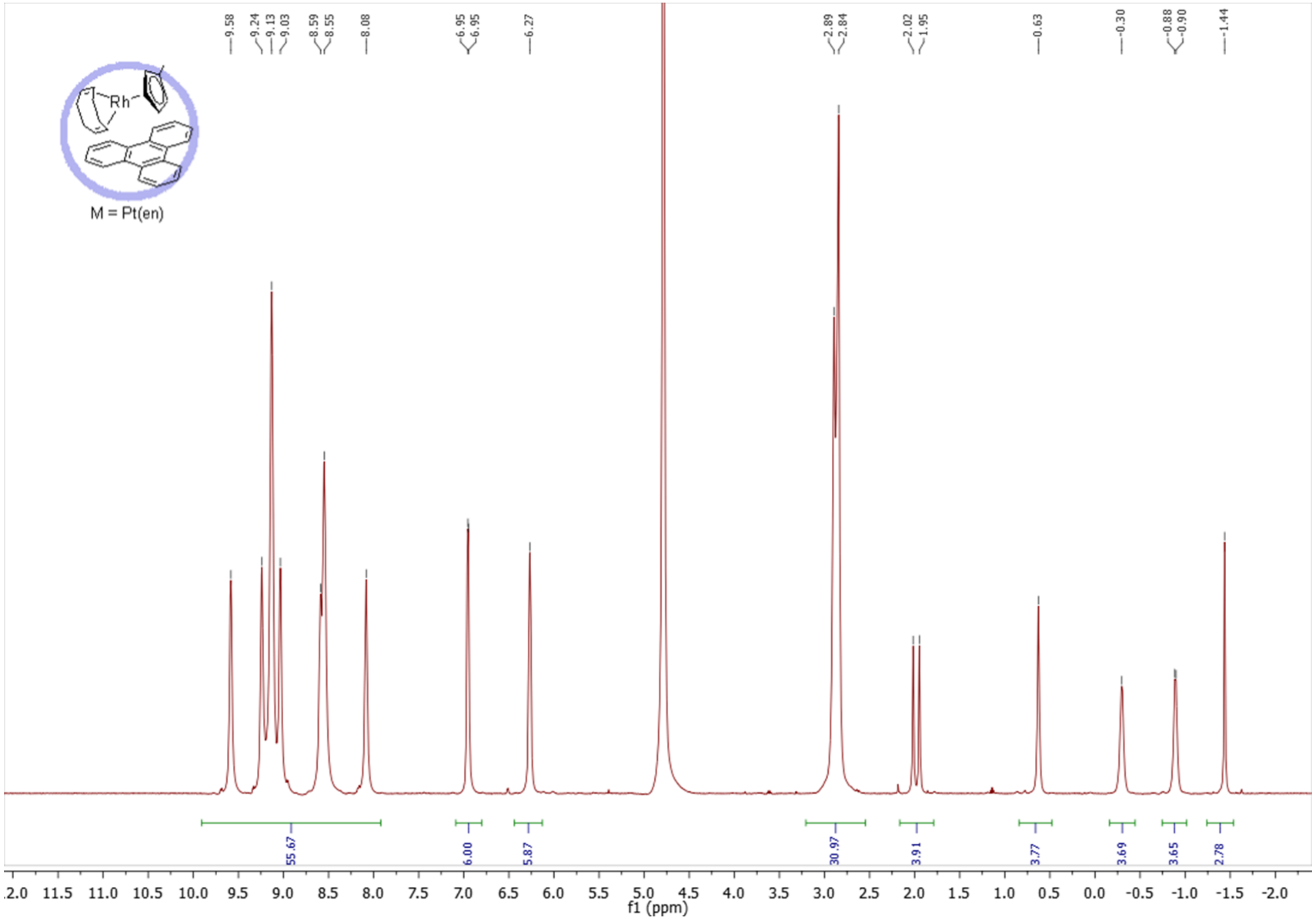

Figure 18: ^{1}H NMR spectrum (500 MHz) of **1a•4•6**

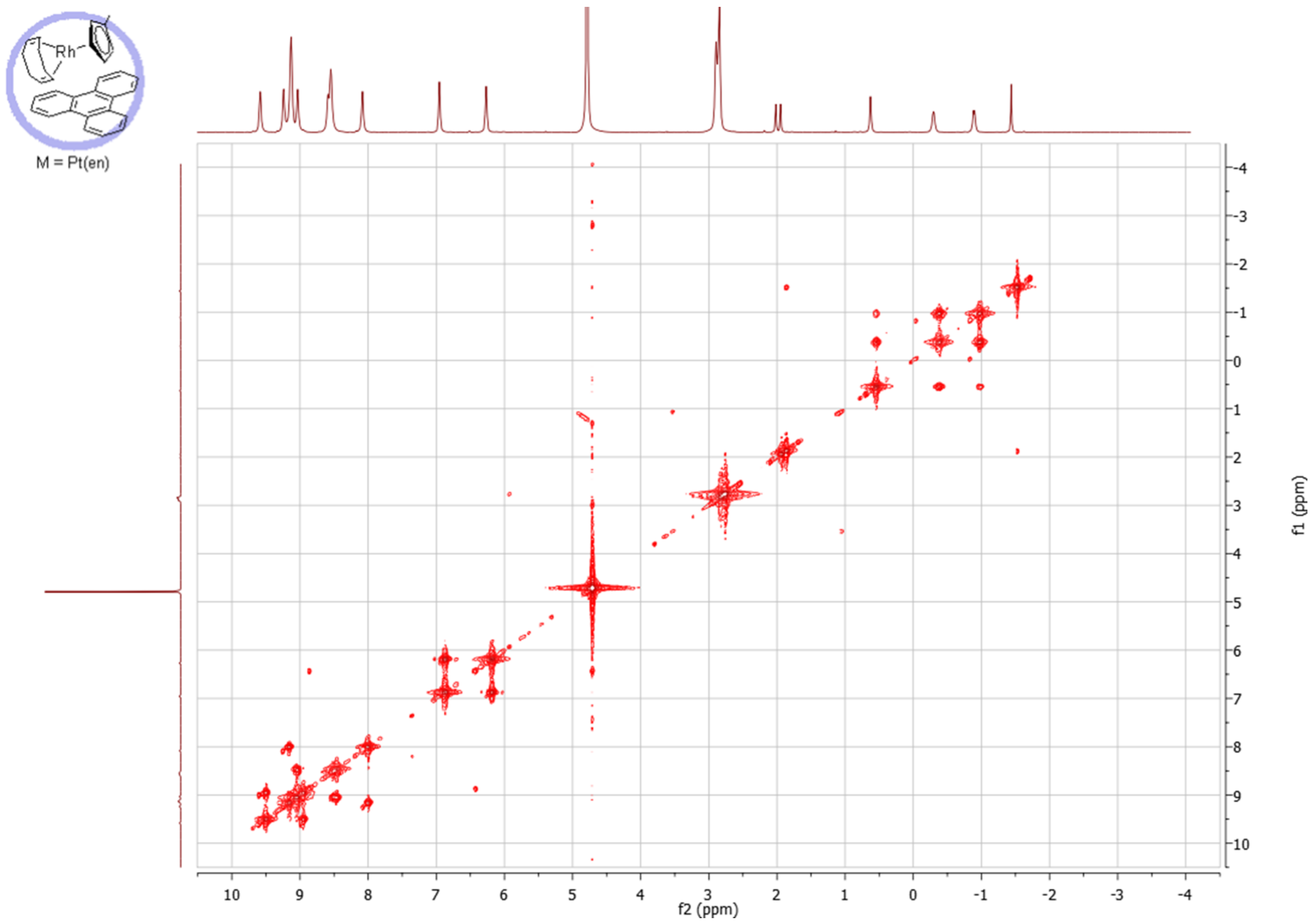

Figure 19: H-H-COSY spectrum of **1a•4•6**

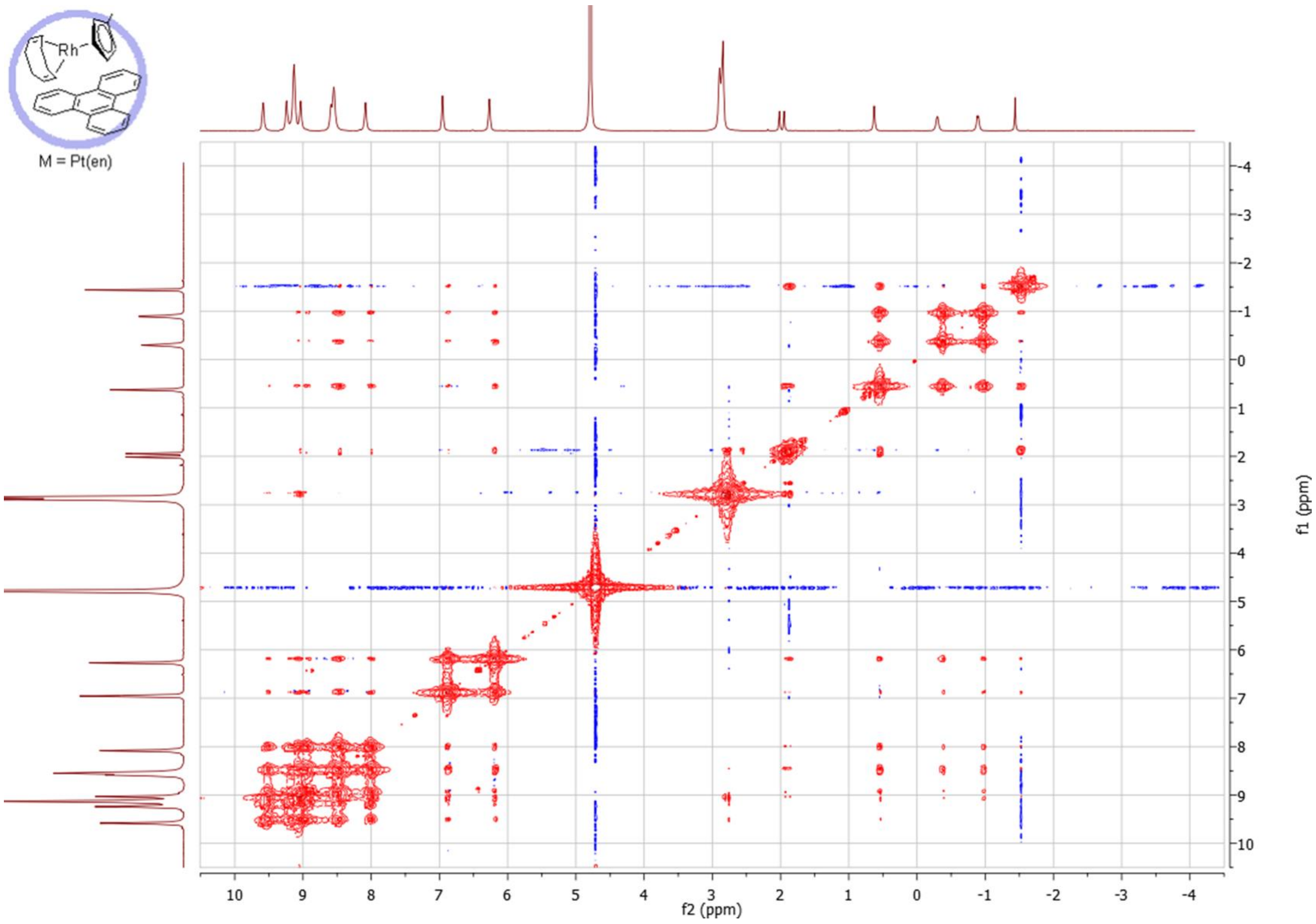

Figure 20: NOESY spectrum of **1a•4•6**

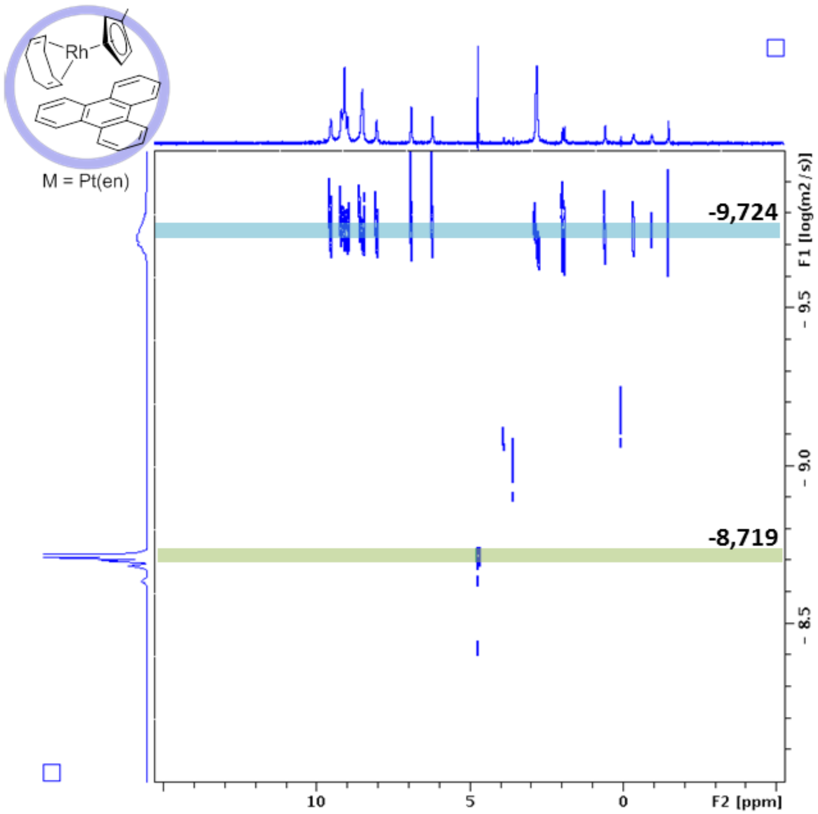

Figure 21: DOSY of **1a•4•6** at 25 °C

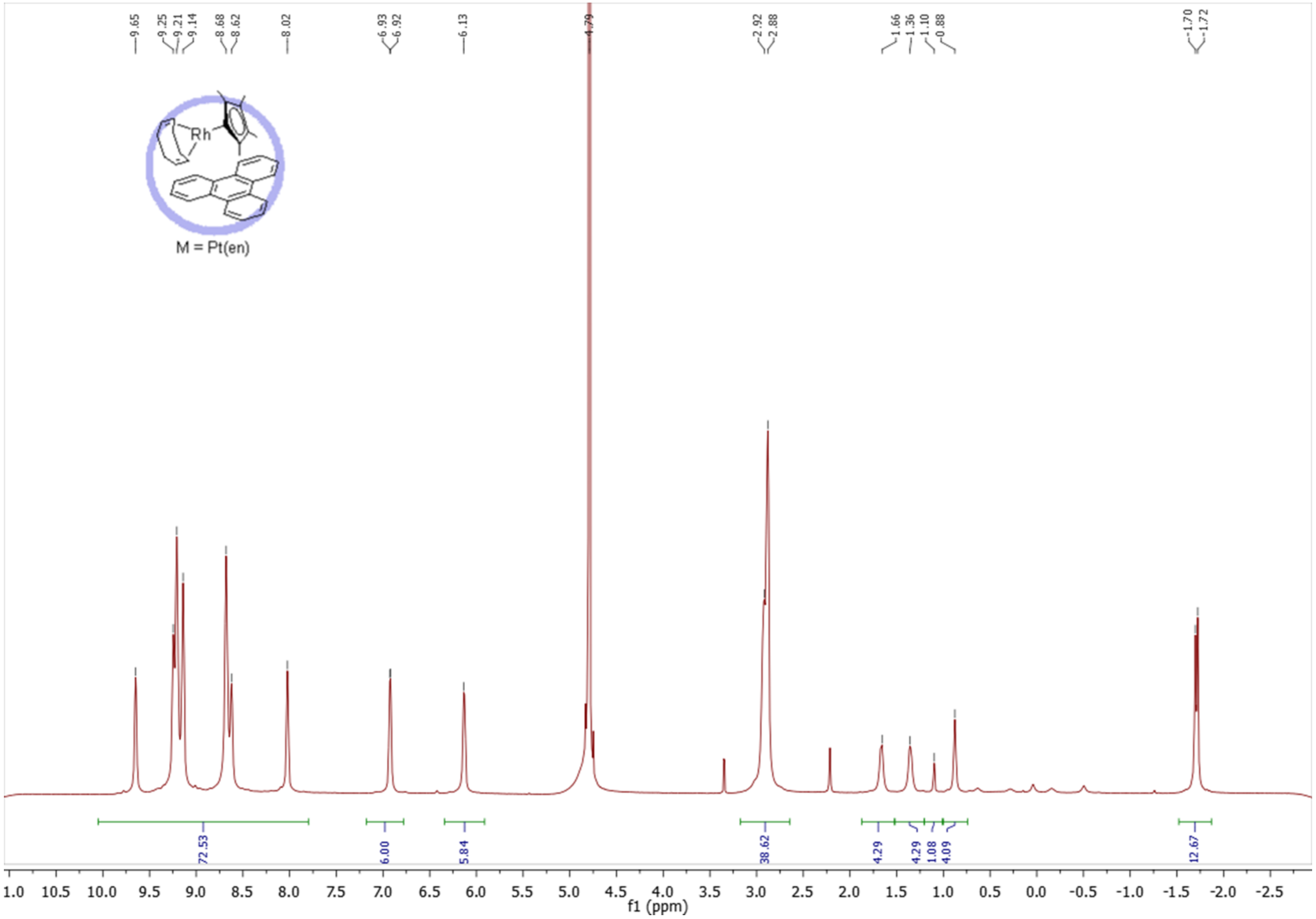

Figure 22: ^{1}H NMR spectrum (500 MHz) of **1a•5•6**

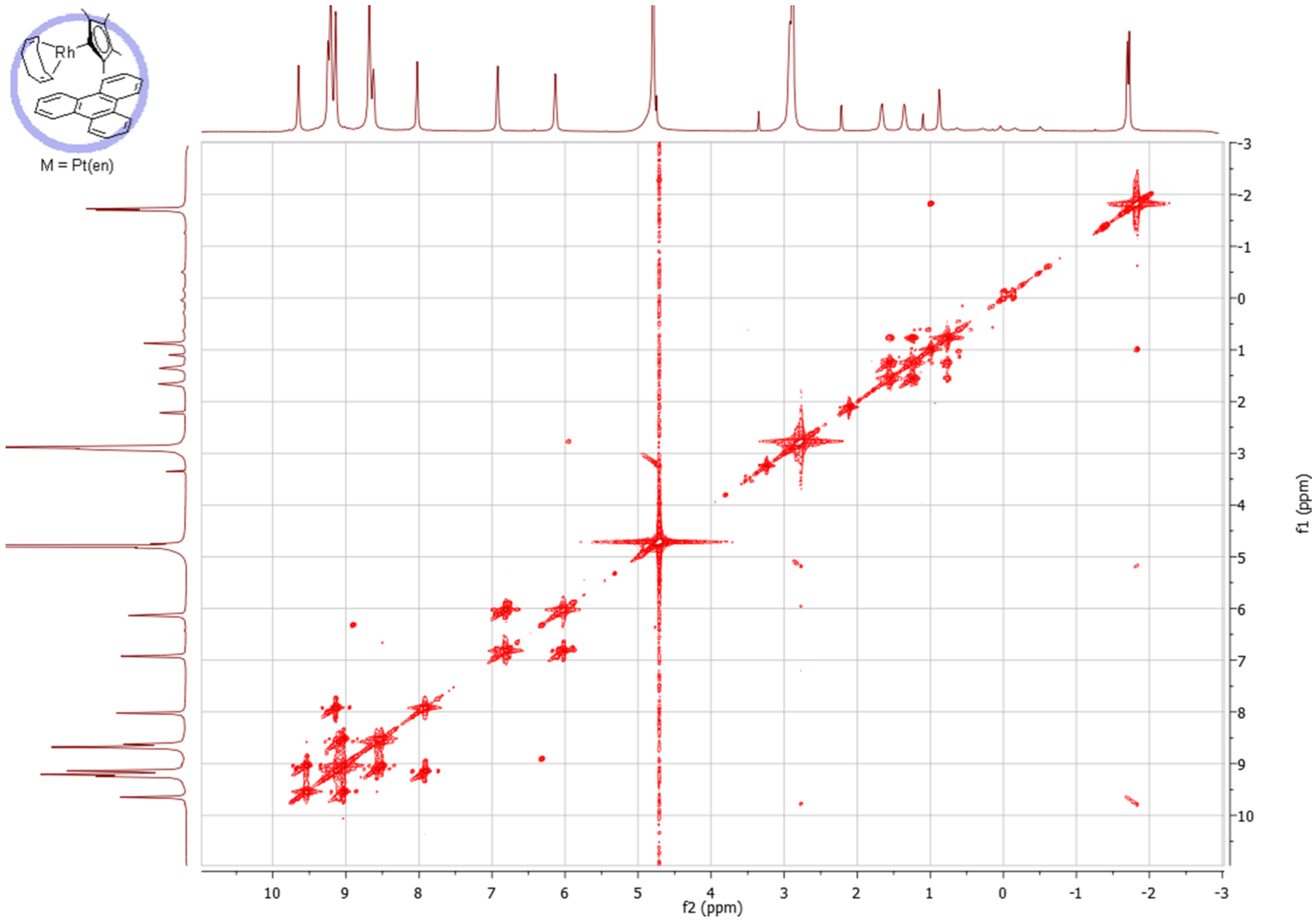

Figure 23: H-H-COSY spectrum of **1a•5•6**

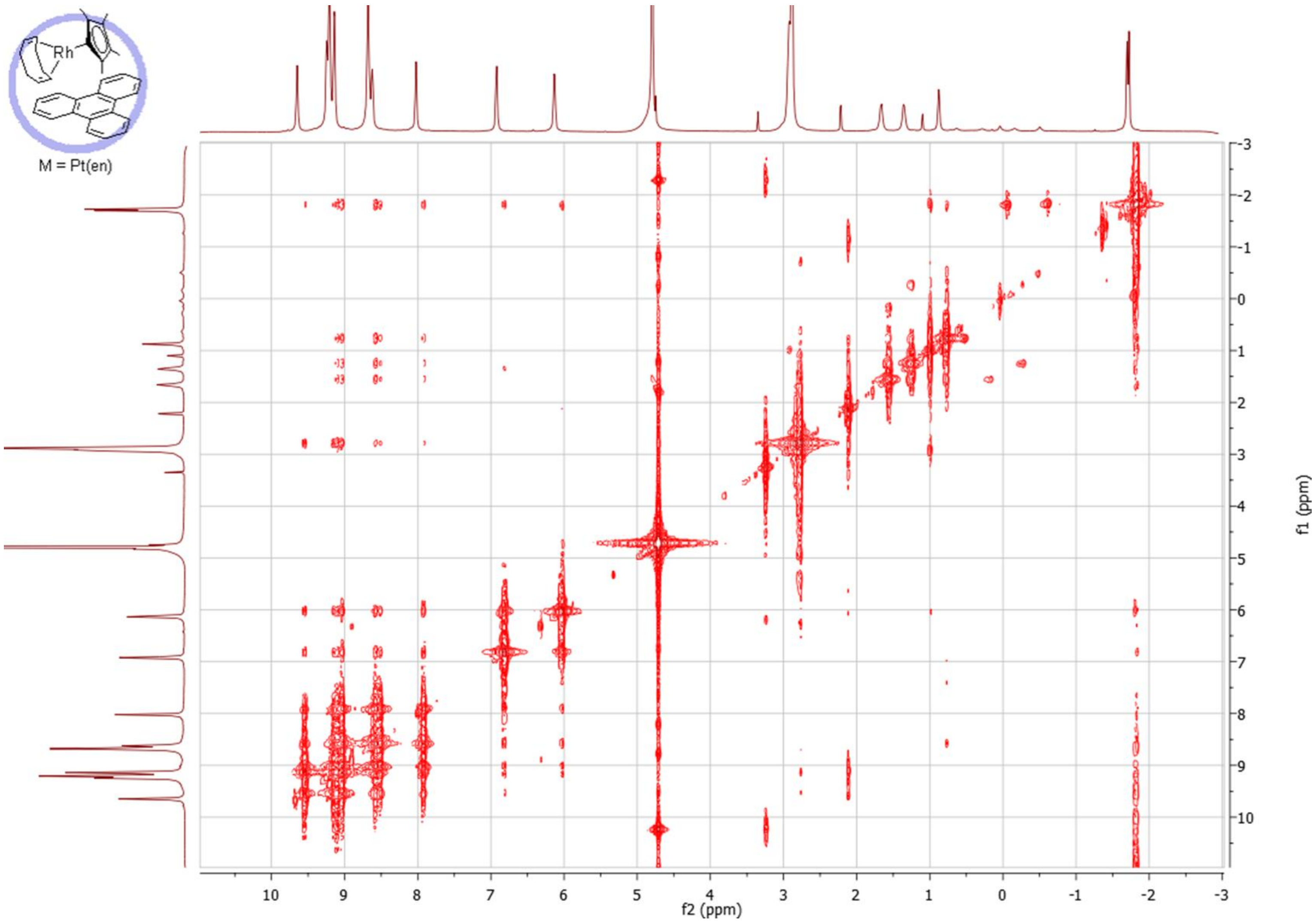

Figure 24: NOESY spectrum of **1a•5•6**

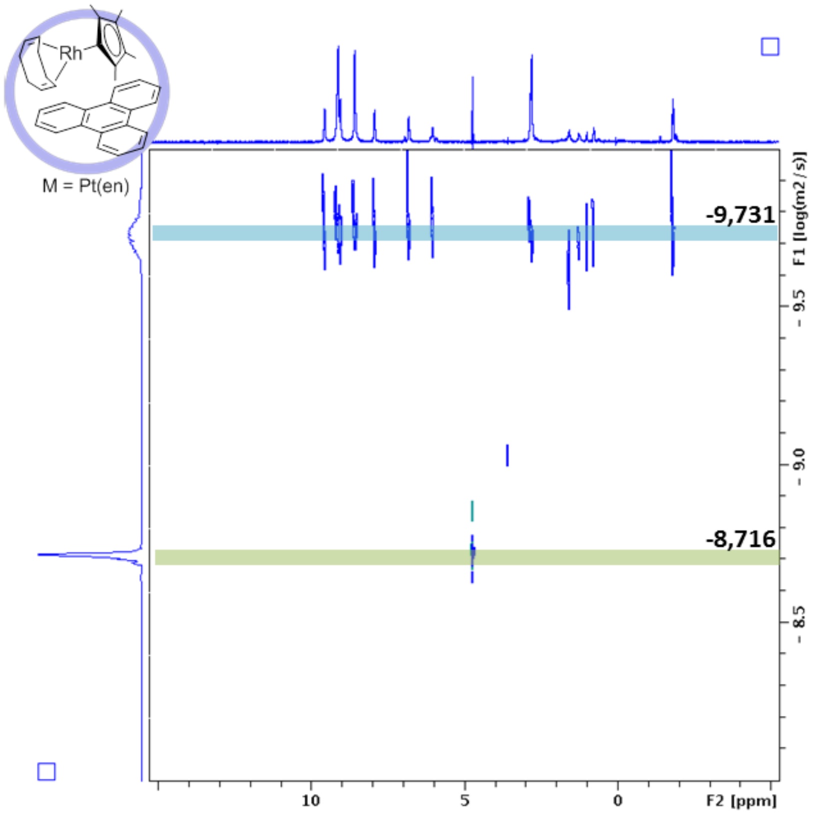

Figure 25: DOSY of **1a•5•6** at 25 °C

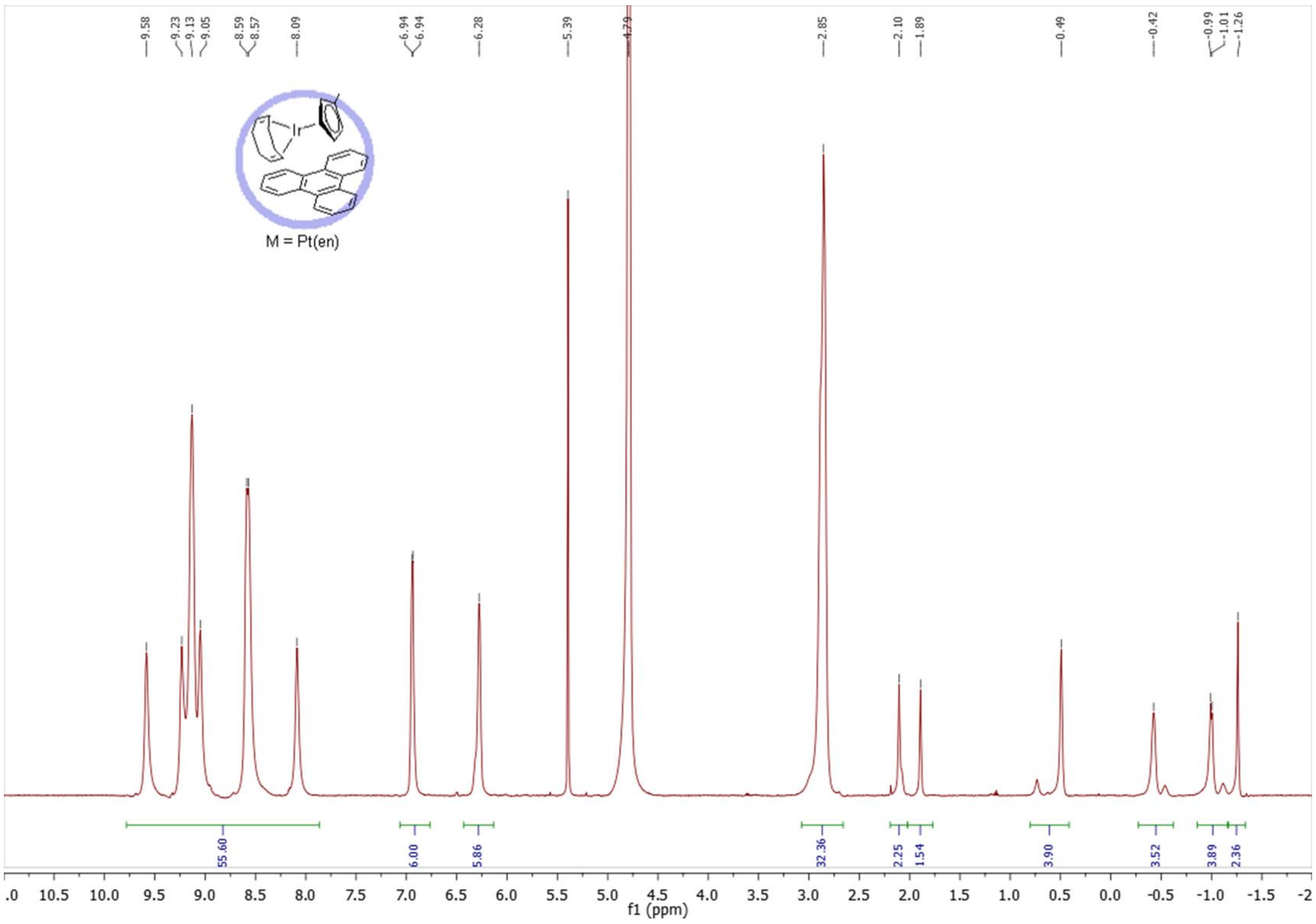

Figure 26: ^{1}H NMR spectrum (500 MHz) of **1a•2•6**

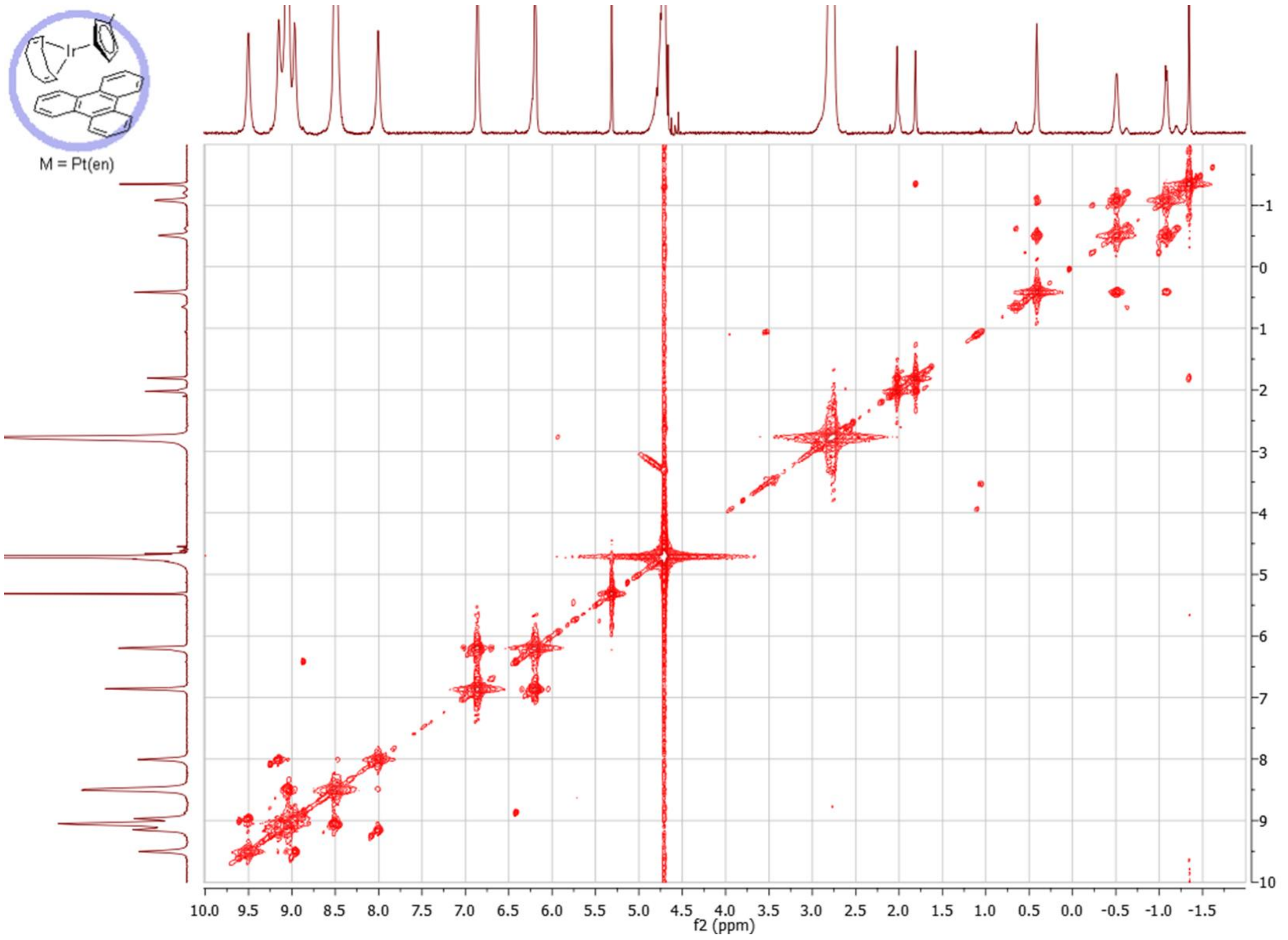

Figure 27: H-H-COSY spectrum of **1a•2•6**

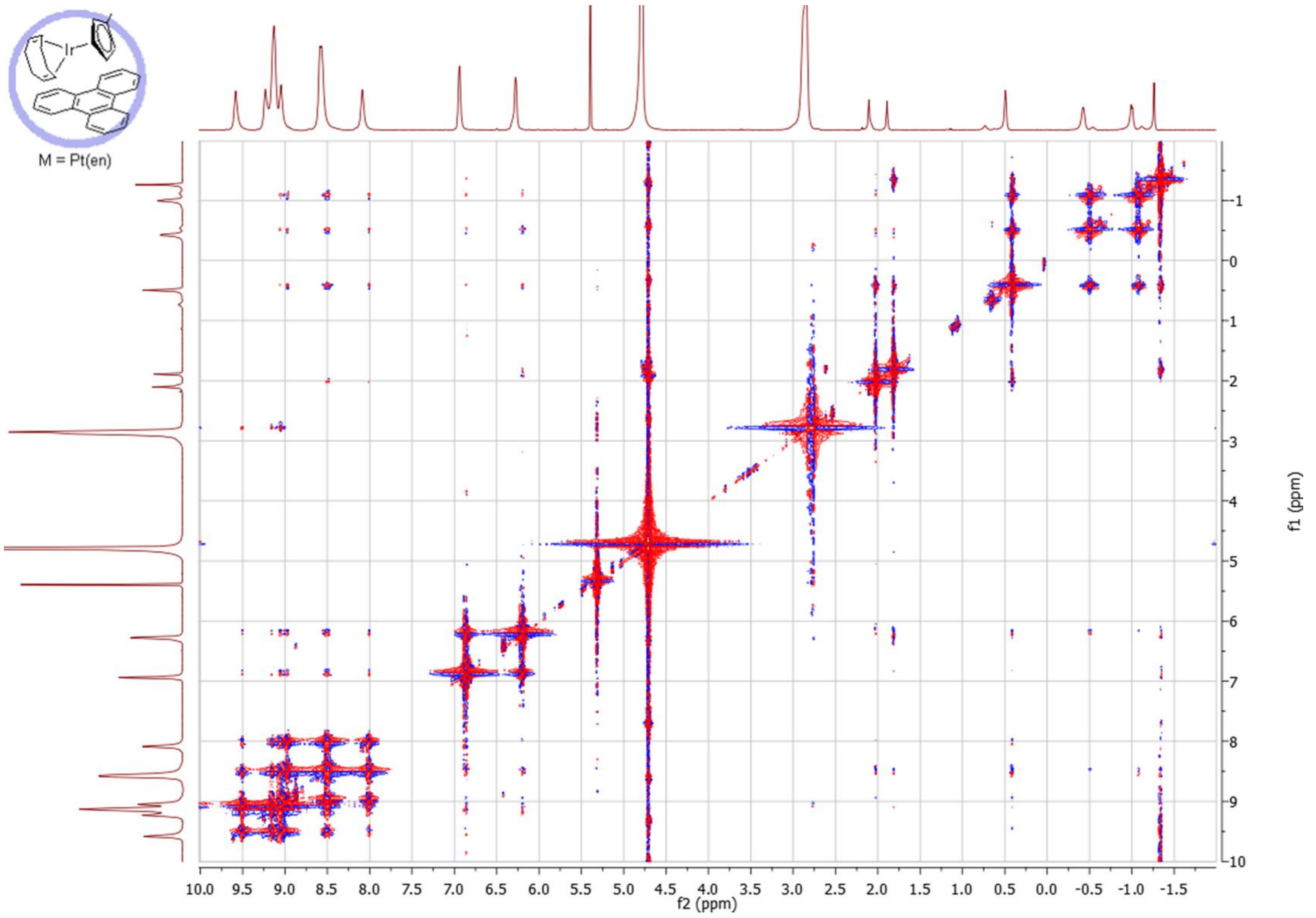

Figure 28: NOESY spectrum of **1a•2•6**

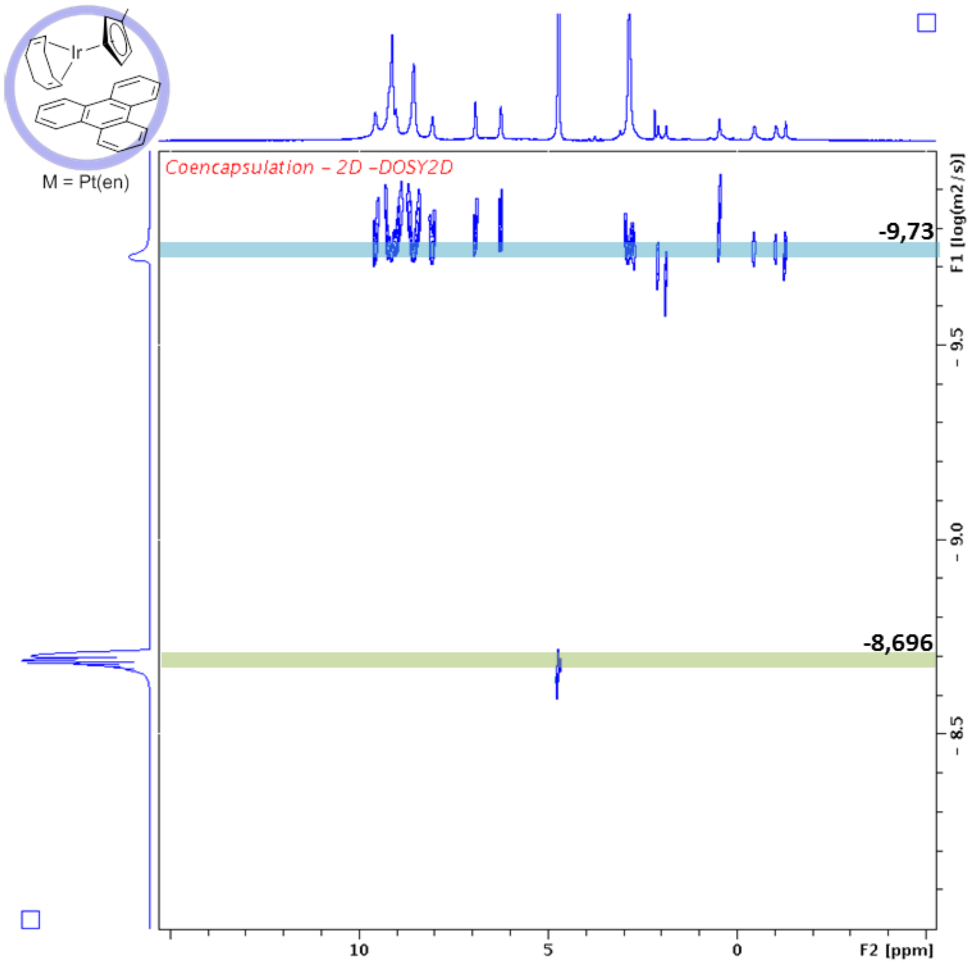

Figure 29: DOSY of **1a•2•6** at 25 °C

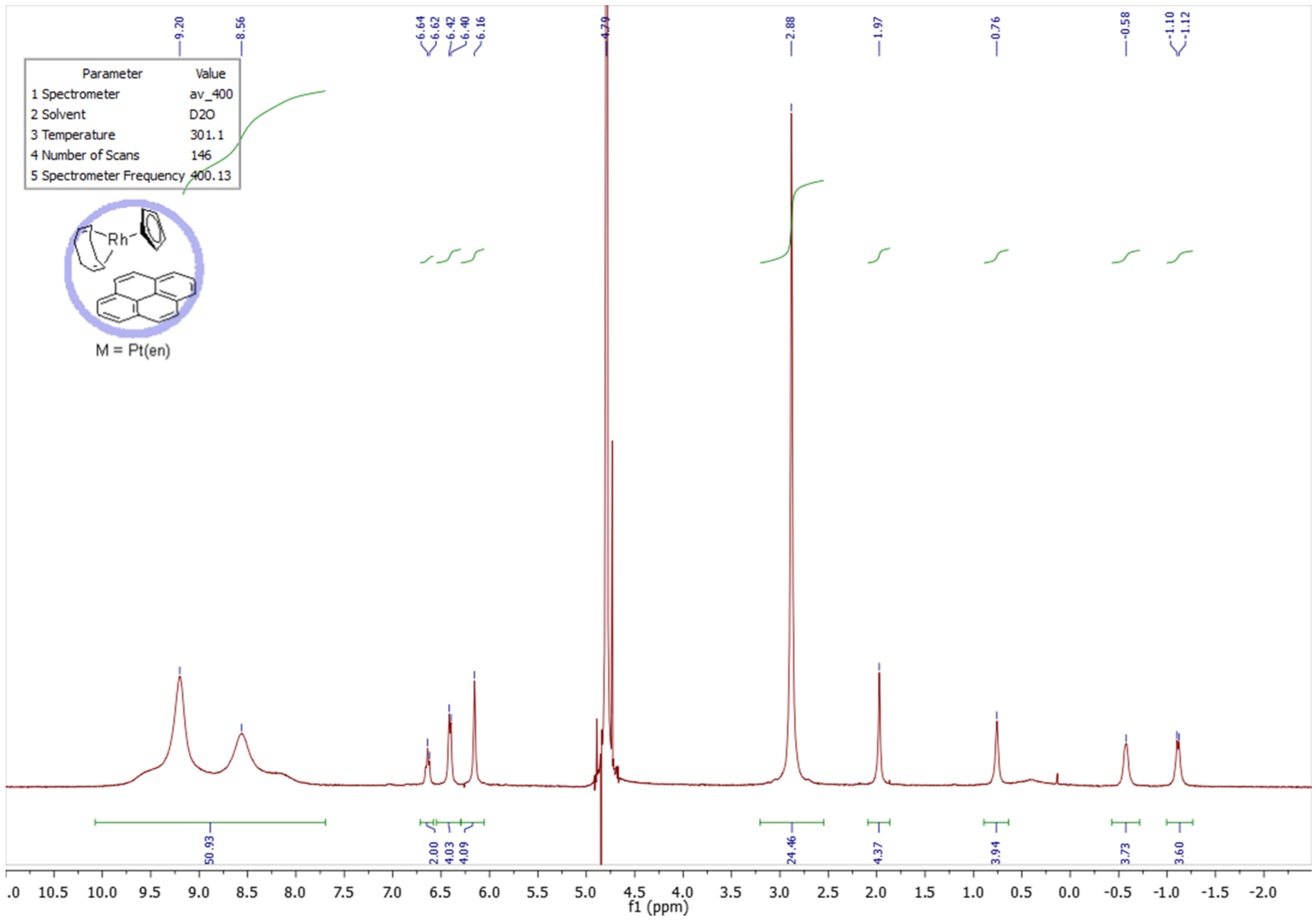

Figure 30: ^{1}H NMR spectrum (400 MHz) of **1a•3•7**

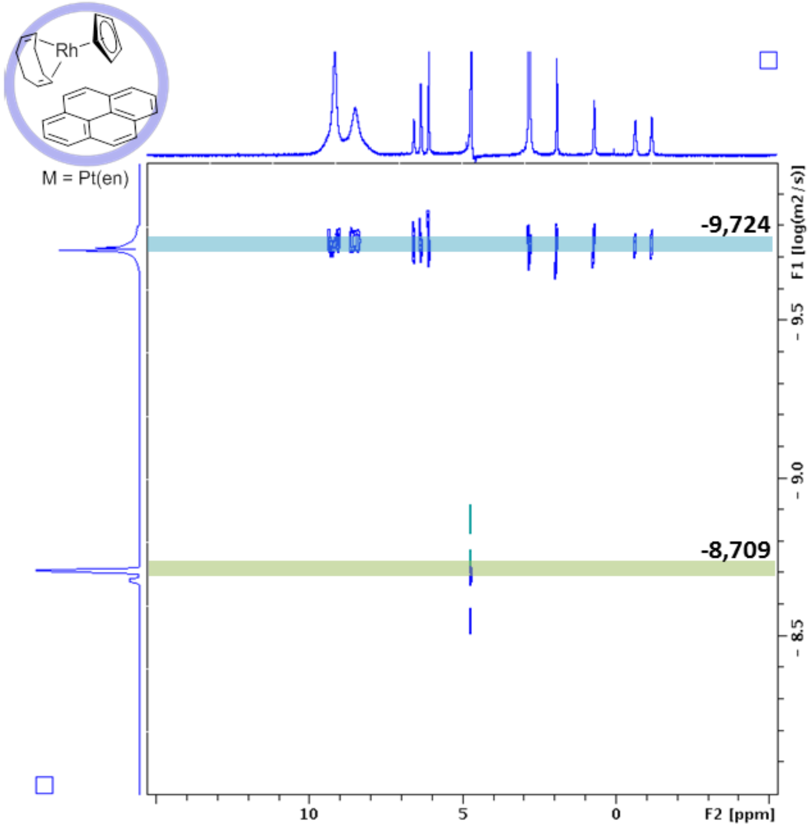

Figure 31: DOSY of **1a•3•7** at 25°C

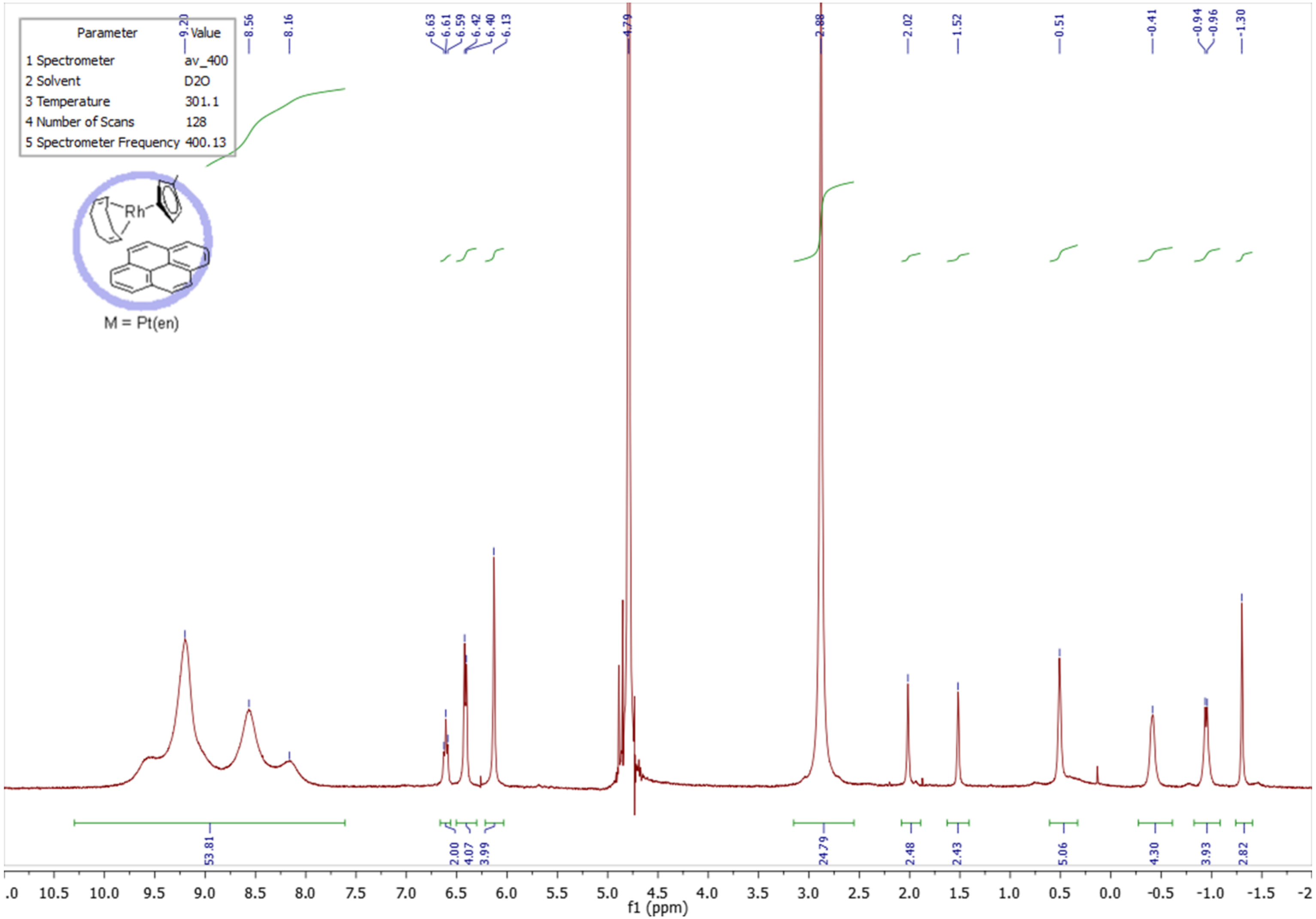

Figure 32: ^{1}H NMR spectrum (400 MHz) of **1a•4•7**

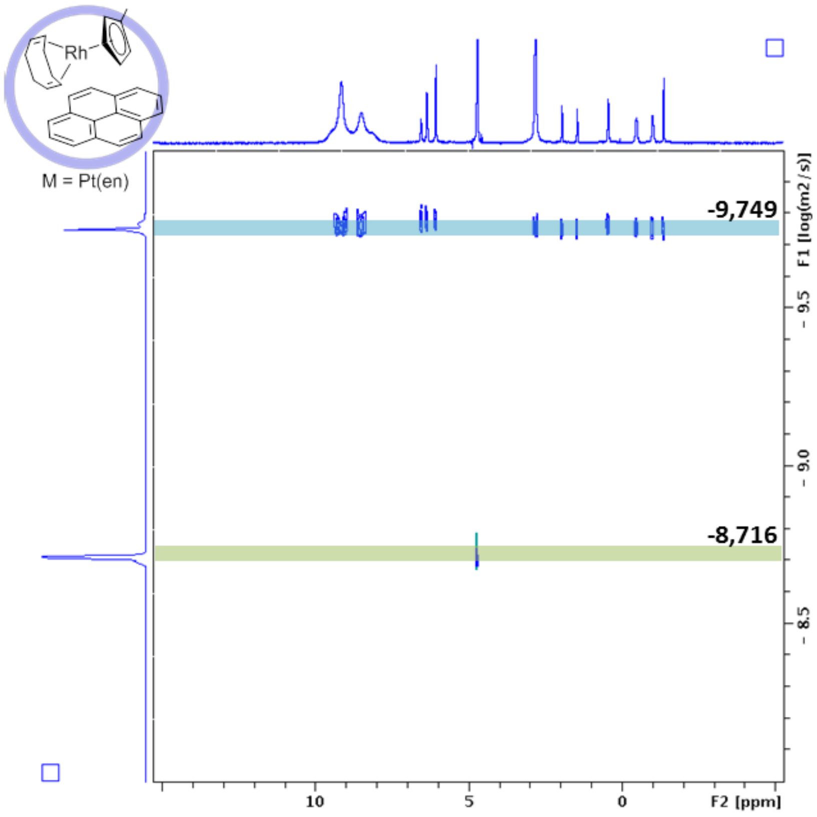

Figure 33: DOSY of **1a•4•7** at 25 °C

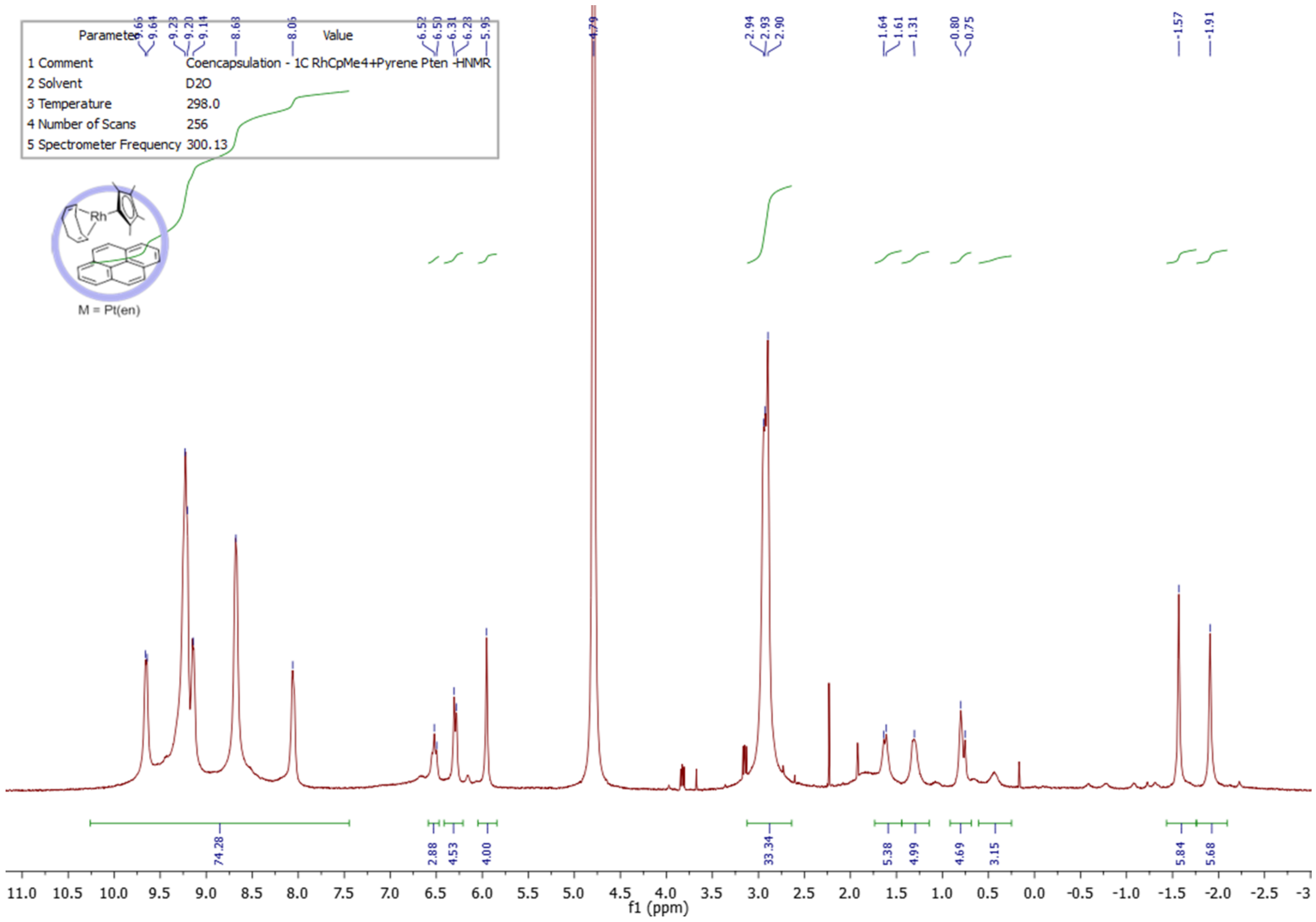

Figure 34: ^{1}H NMR spectrum (300 MHz) of **1a•5•7**

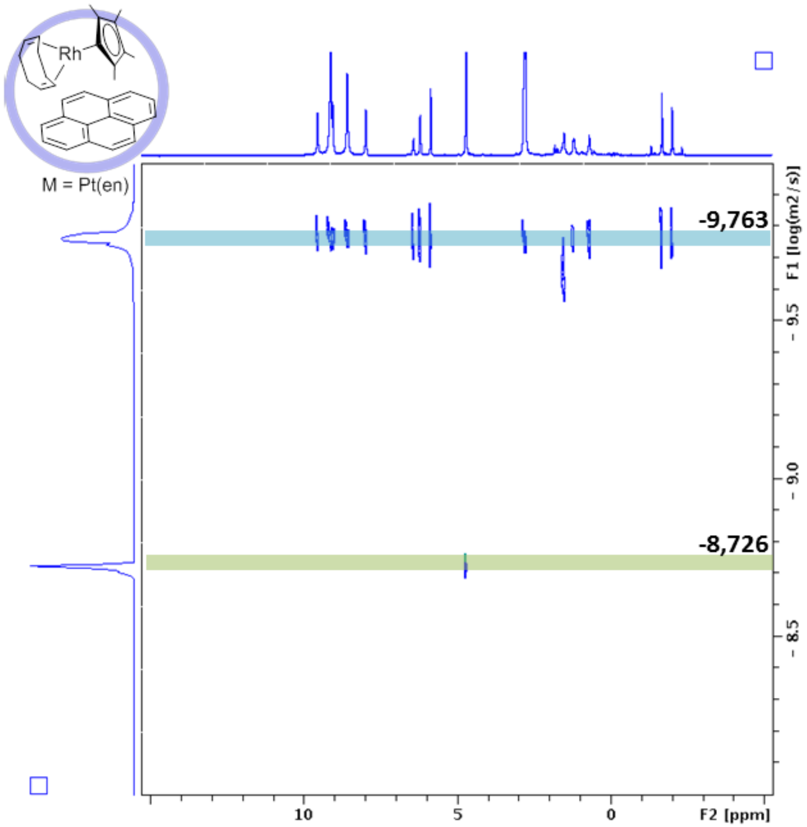

Figure 35: DOSY of **1a•5•7** at 25 °C

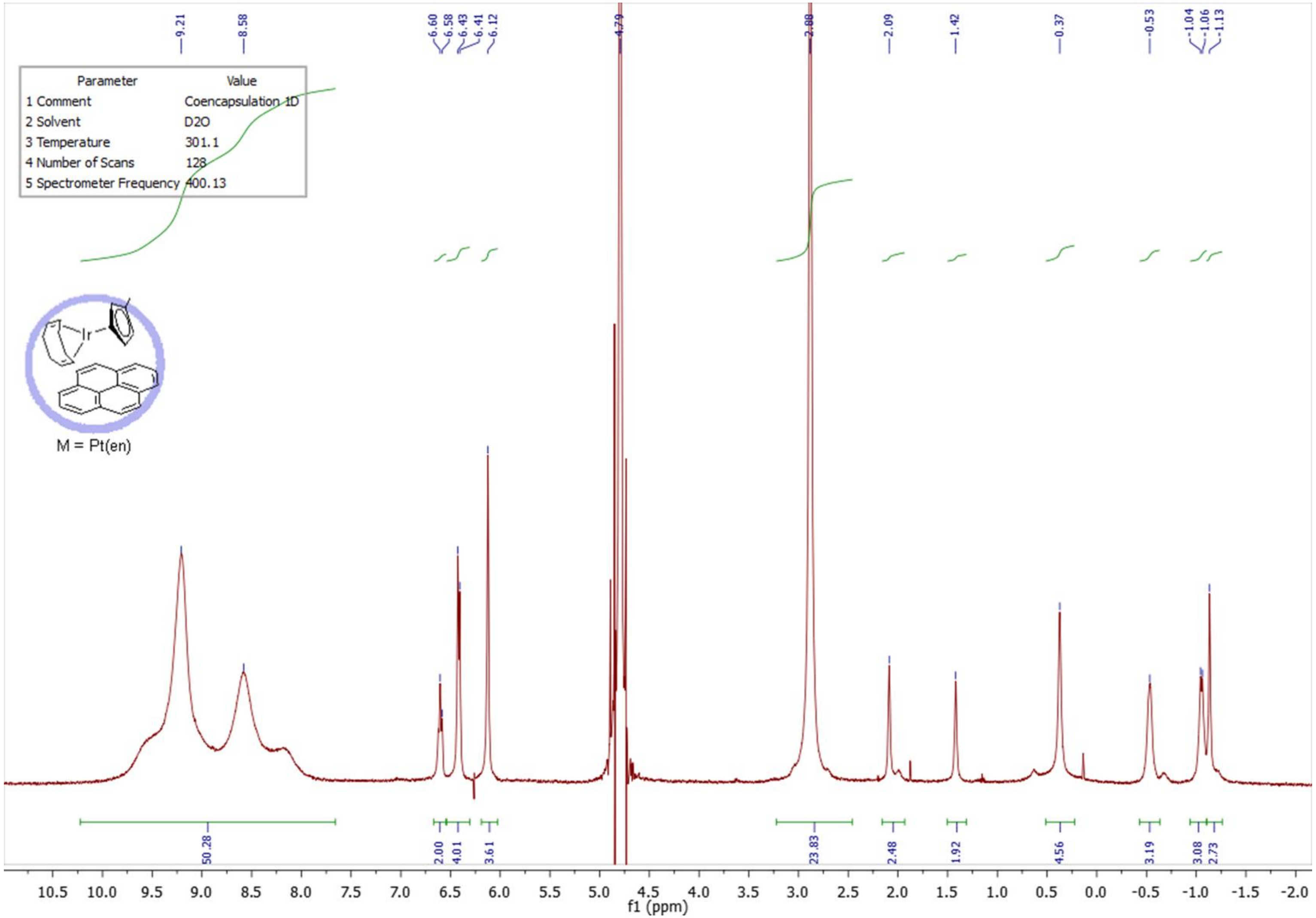

Figure 36: ^{1}H NMR spectrum (400 MHz) of **1a•2•7**

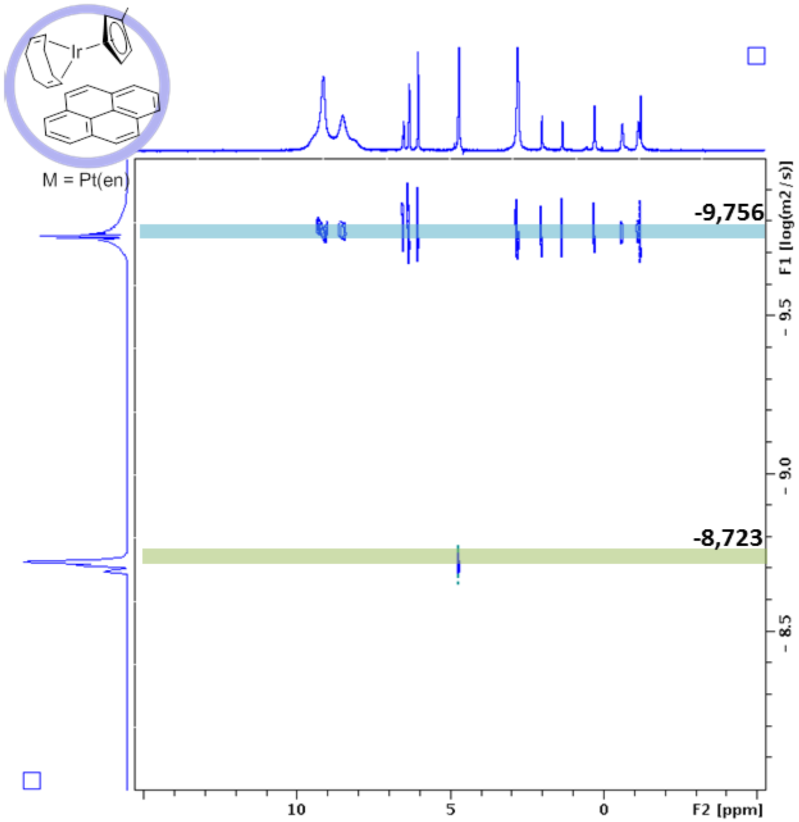

Figure 37: DOSY NMR of **1a•2•7** at 25 °C

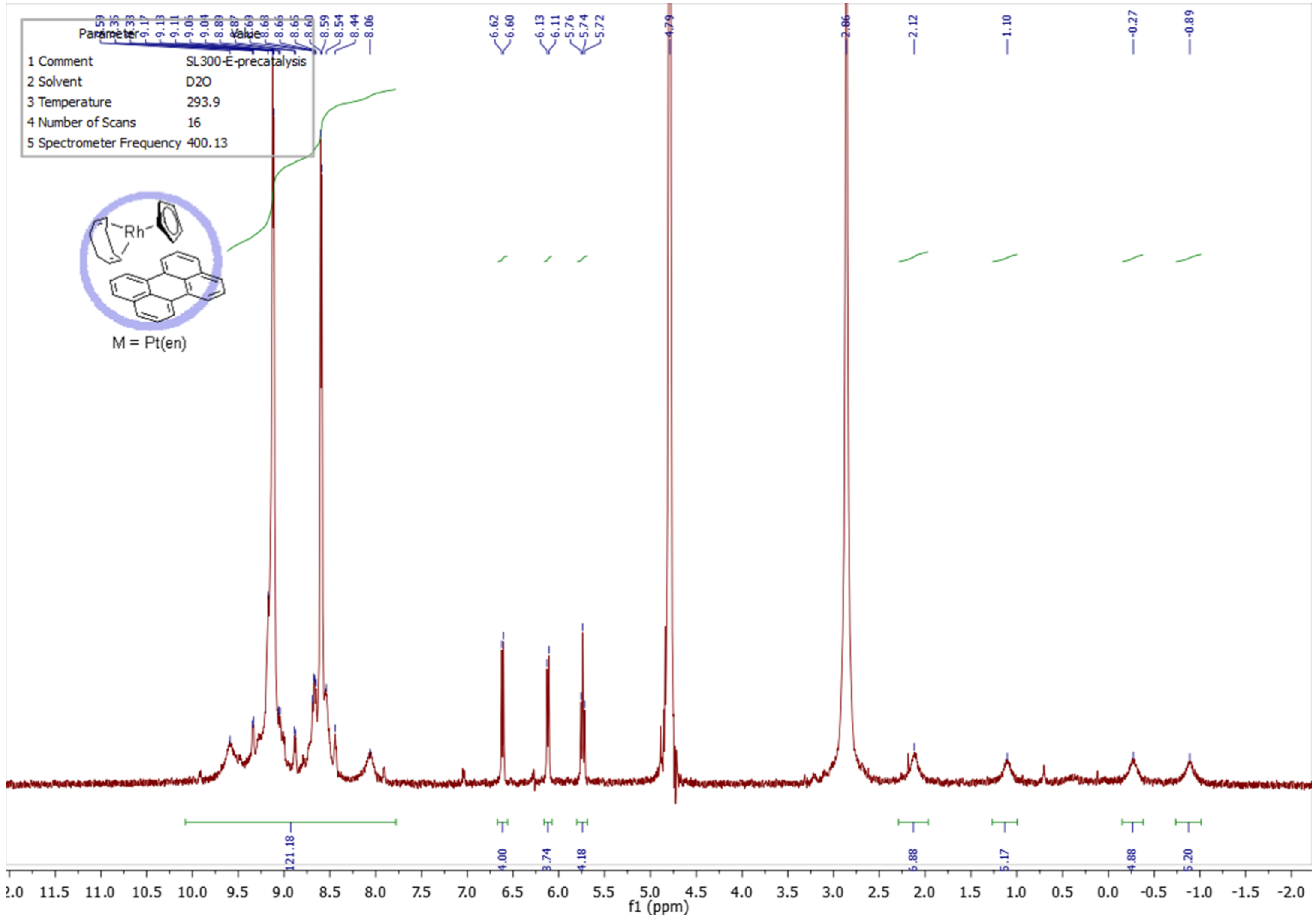

Figure 38: ^{1}H NMR spectrum (400 MHz) of **1a•3•8**

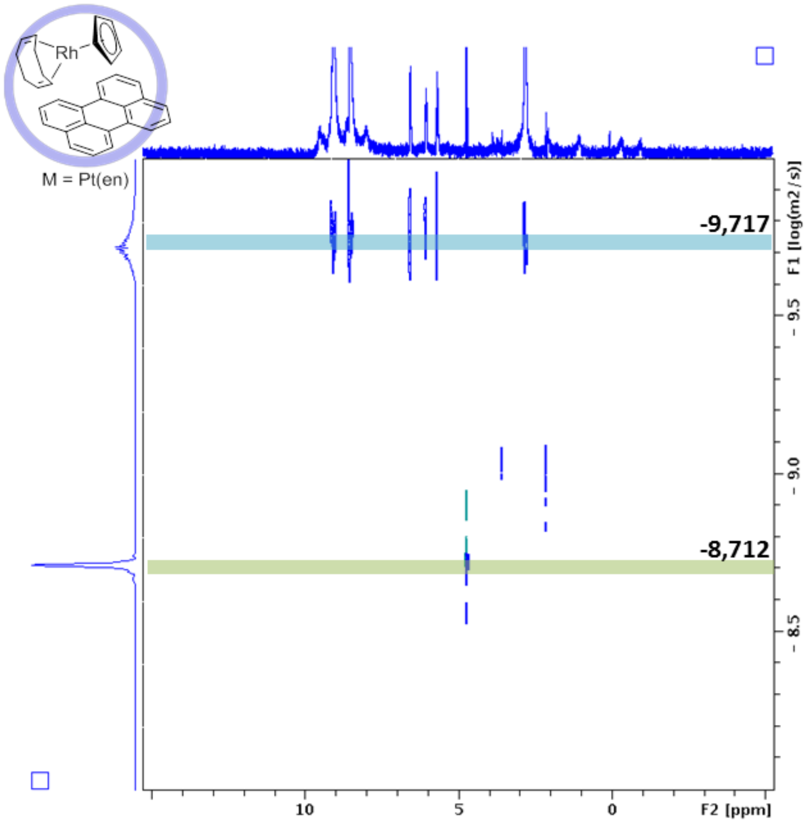

Figure 39: DOSY NMR of **1a•3•8** at 25 °C**.** Note that due to the low concentration, signals of the rhodium complex are missing.

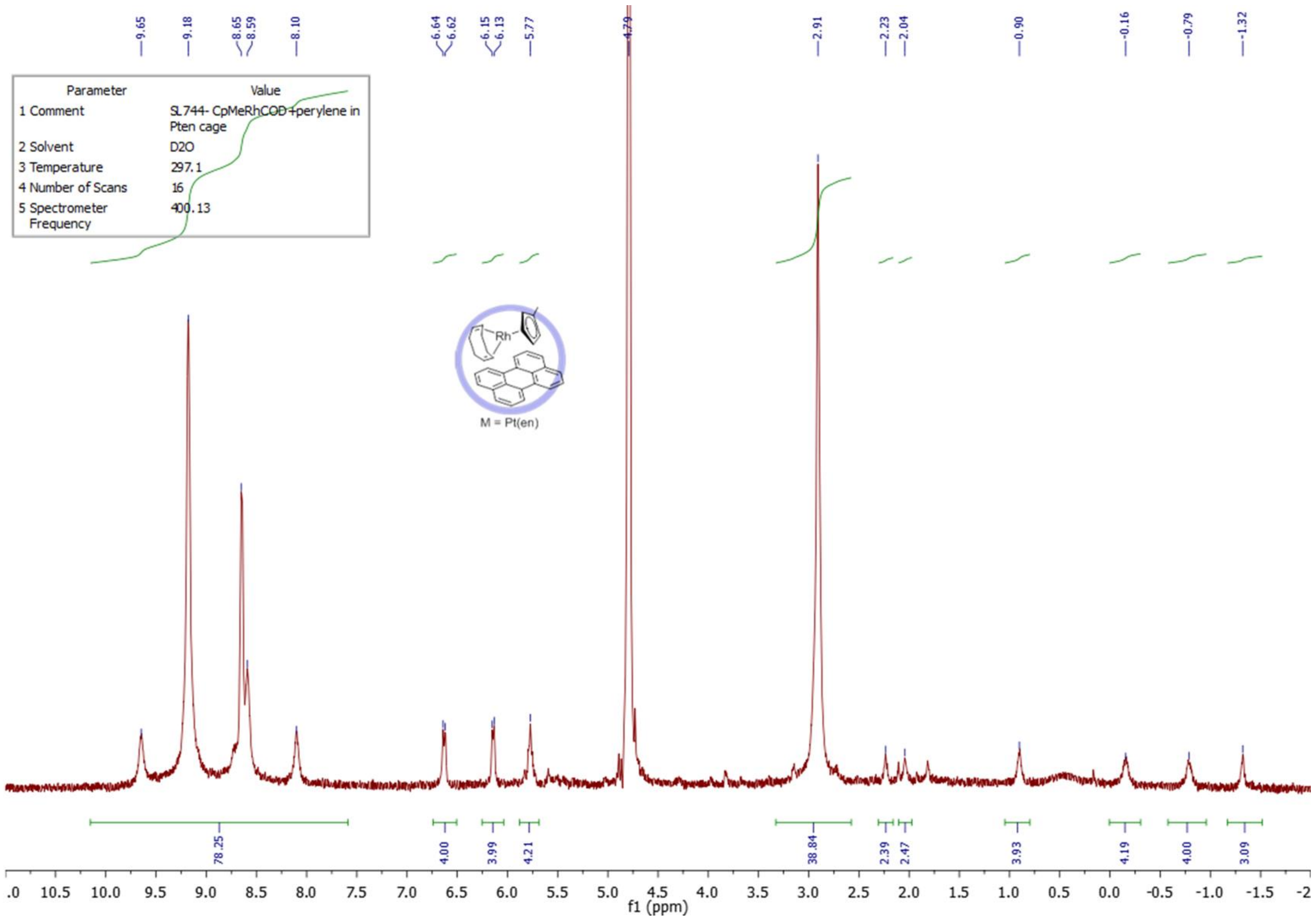

Figure 40: ^{1}H NMR spectrum (400 MHz) of **1a•4•8**

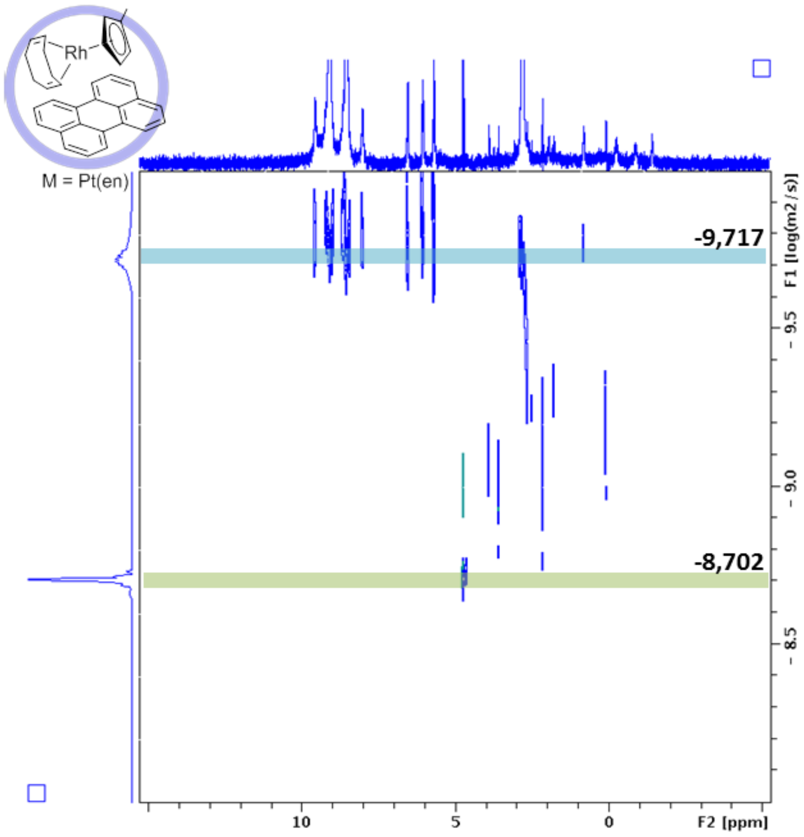

Figure 41: DOSY NMR of **1a•4•8** at 25 °C**.** Note that due to the low concentration, signals of the rhodium complex are missing.

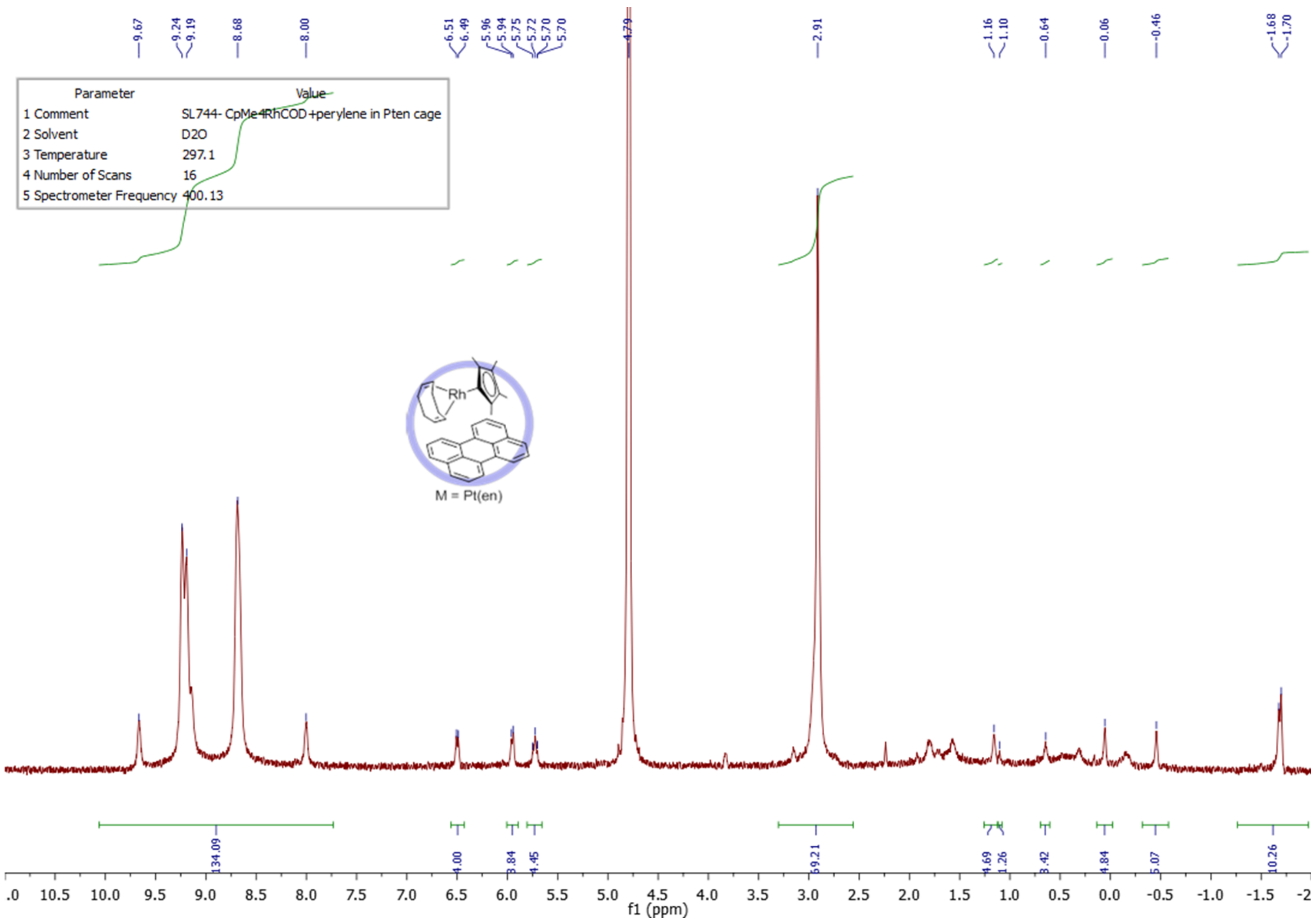

Figure 42: ^{1}H NMR spectrum (400 MHz) of **1a•5•8**

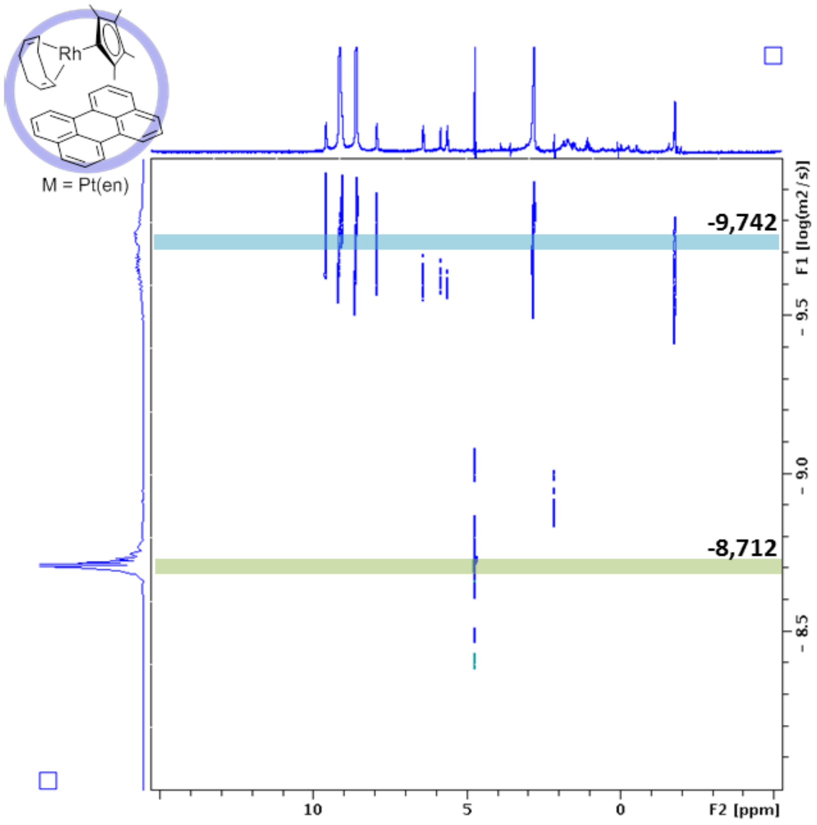

Figure 43: DOSY NMR of **1a•5•8** at 25 °C. Note that due to the low concentration, signals of the rhodium complex are missing.

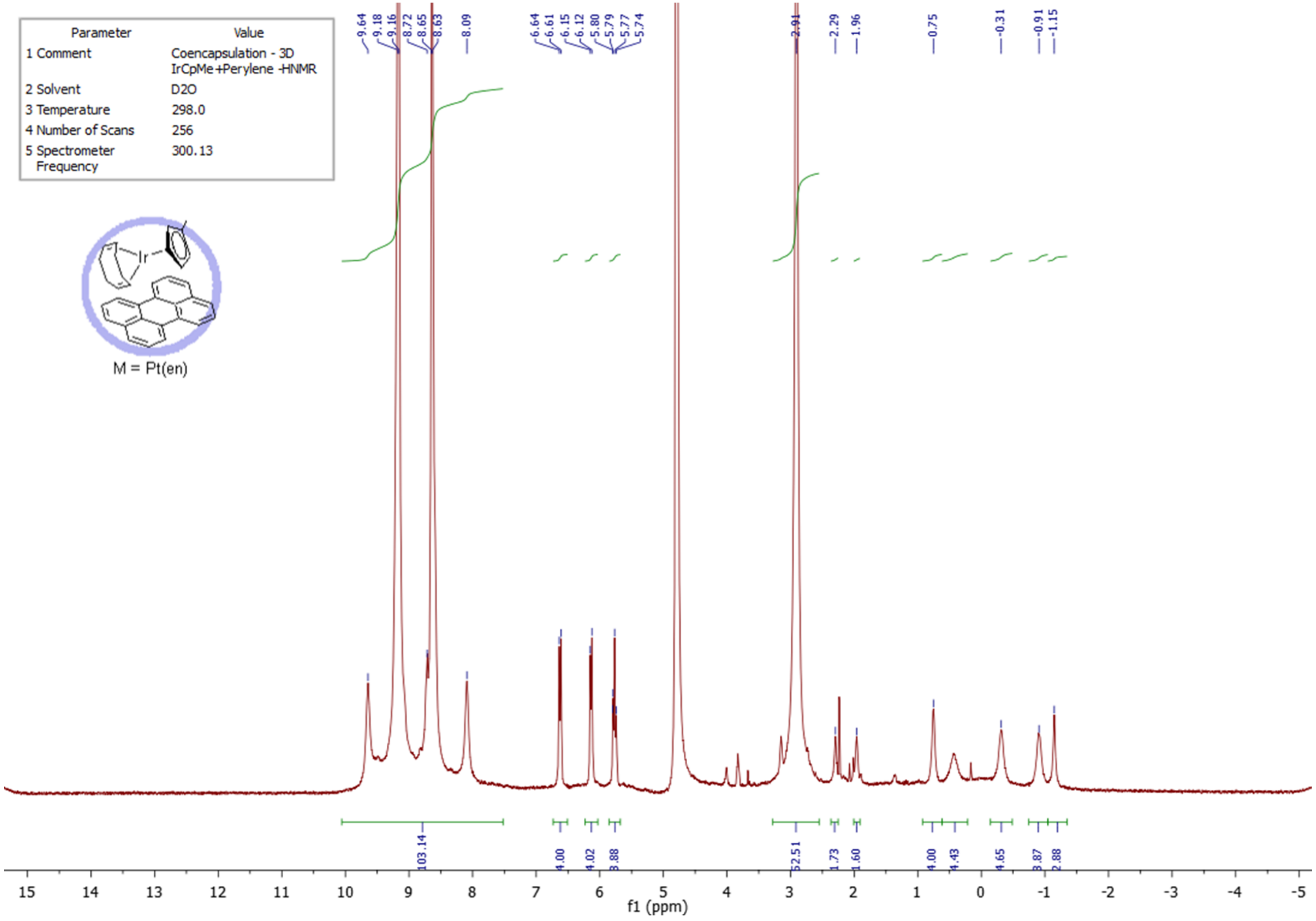

Figure 44: ^{1}H NMR spectrum (300 MHz) of **1a•2•8**

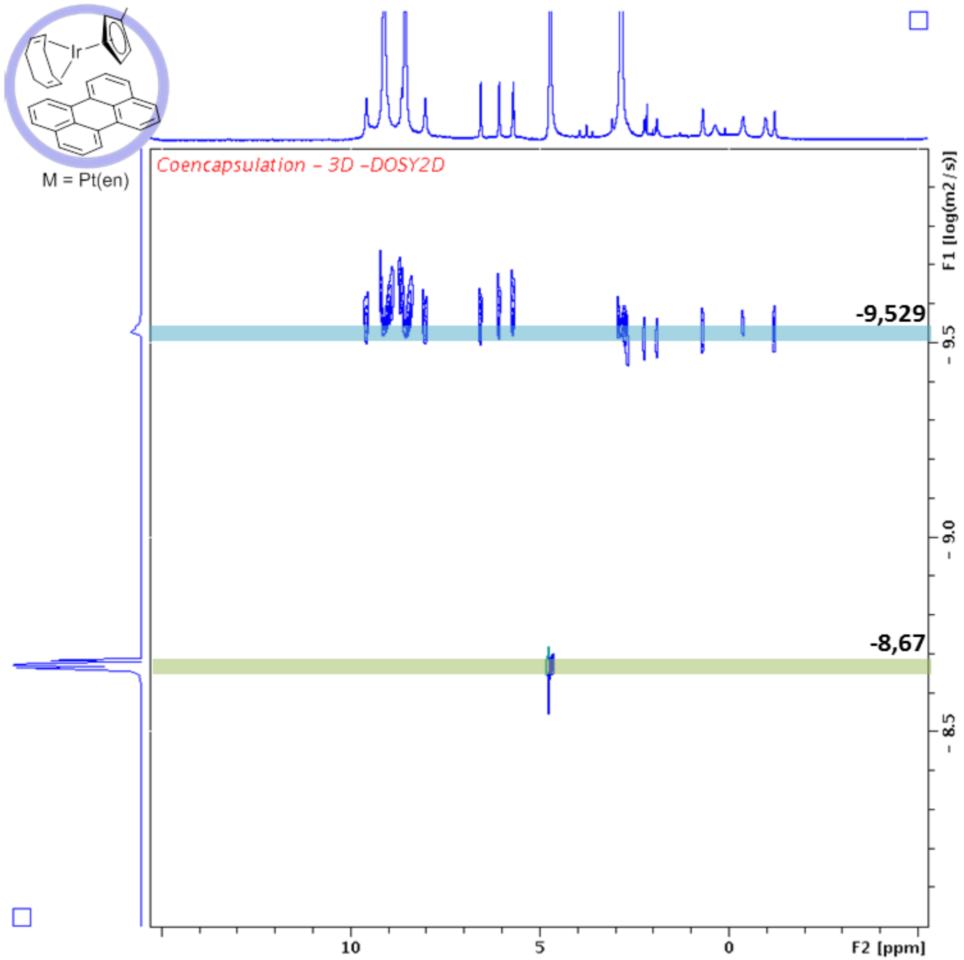

Figure 45: DOSY NMR of **1a•2•8** at 25 °C

S9. References

[1] M. Holz, X. Mao, D. Seiferling, A. Sacco, *J. Chem. Phys.*, 1996, **104**, 669.

[2] M. D. Živković, D. P. Ašanin, S. Rajković, M. I. Djuran, *Polyhedron* 2011, **30**, 947–52.

[3] D. Whang, K. Park, J. Heo, P. Ashton, K. Kim, *J. Am. Chem. Soc.,* 1998, **120**, 4899–900.

[4] J. Vicente, J. Abad, M. Chicote, M.-D. Abrisqueta, J.-A. Lorca, M. C. R. Arellano de, *Organometallics*, 1998, **17**, 1564–8.

[5] H. L. Anderson, S. Anderson, J. K. M. Sanders, *J. Chem. Soc., Perkin Trans. 1*, 1995, 2231–45.

[6] F. Ibukuro, T. Kusukawa, M. Fujita, *J. Am. Chem. Soc.*, 1998, **120**, 8561–2.

[7] E. M. López-Vidal, V. Blanco, M. D. García, C. Peinador, J. M. Quintela, *Org. Lett.,* 2012, **14**, 580–3.

[8] M. Fujita, D. Oguro, M. Miyazawa, H. Oka, K. Yamaguchi, K. Ogura, *Nature* 1995, **378**, 469–71.

[9] J. W. Kang, K. Moseley, P. M. Maitlis, *J. Am. Chem. Soc.*, 1969, **91**, 5970–7.

[10] D. Selent, M. Ramm, *Journal of Organometallic Chemistry*, 1995, **485**, 135–40.

[11] a) Y. Kobayashi, M. Kawano, and M. Fujita, *Chem. Commun.*, 2006, 4377–9. b) S. Horiuchi, T. Murase, and M. Fujita, *Chem. Asian J.*, 2011, **6**, 1839–47.

[12] a) D. B. Gil, A. M. de la Peña, J. A. Arancibia, G. M. Escandar, A. C. Olivieri, *Anal. Chem.,* 2006, **78**, 8051–8. b) E. C. Constable, M. Neuburger, P. Rösel, G. E. Schneider, J. A. Zampese, C. E. Housecroft, Monti, N. Armaroli, R. D. Costa, E. Ortí, *Inorg. Chem.,* 2013, **52**, 885–97.
